# Comparative dynamics of coffee–tea cultural spaces in two Chinese cities: Evidence from Qingdao and Jinan, 2018–2024

**DOI:** 10.1371/journal.pone.0355398

**Published:** 2026-08-03

**Authors:** Xulan Li, Ying Zhang, Ying Wang, Xinwen Zhang, Yihan Wang, Yihan Fu, Fengyi Li

**Affiliations:** 1 College of Landscape Architecture and Forestry, Qingdao Agricultural University, Qingdao, Shandong, China; 2 School of Civil Engineering and Transportation, Weifang University, Weifang, Shandong, China; 3 College of Landscape Architecture and Architecture, Zhejiang A&F University, Hangzhou, Zhejiang, China; 4 School of Accounting and Finance, Qingdao City University, Qingdao, Shandong, China; University of Jinan, CHINA

## Abstract

Coffee shops and teahouses are important everyday consumption venues through which changes in urban consumption-space organization can be observed. Existing studies have often examined them separately, leaving limited comparative evidence on how their coexistence, relative balance, directional change, and functional embedding differ across contrasting urban contexts. This study compares Jinan and Qingdao, two major cities in Shandong Province with different historical and functional settings, using annual point-of-interest (POI) data from 2018 to 2024 within a unified 500 m × 500 m grid framework. Coffee Ratio (CR), Hybridization Index (HI), and Cultural Transition Intensity (CTI) were combined with long-run cultural zones, temporal trajectory types, functional-zone context, and supplementary robustness checks to examine POI-observed coffee–tea venue patterns. The results show that coffee–tea venue change cannot be understood as a simple process in which coffee replaces tea. Jinan showed substantial overall expansion in coffee-shop and teahouse POIs, with total counts increasing by 64.6% from 2018 to 2024, while coffee share changed only slightly from 42.6% to 43.1%. By contrast, Qingdao showed little overall growth in total counts (+0.9%) but a marked compositional shift toward coffee, with coffee share increasing from 49.3% to 59.1%. Grid-level results further indicate that Qingdao had clearer coffee-oriented restructuring, a higher coffee-dominant share than Jinan, and more stable coffee trajectories, whereas Jinan retained a stronger tea-oriented base, higher coexistence balance, and more bidirectional adjustment. Functional-zone analyses showed significant contextual associations in both cities, with clearer functional differentiation in Qingdao and stronger links between mixed-use settings and cultural hybridity in Jinan. Overall, the findings suggest that POI-observed coffee–tea venue restructuring is better understood as differentiated reorganization characterized by coexistence, selective concentration, and uneven directional change rather than as a single linear process of substitution.

## 1 Introduction

In recent years, consumption and leisure spaces in Chinese cities have undergone continuous restructuring in response to consumption upgrading, urban renewal, and changes in everyday lifestyles [1]. Among these spaces, coffee shops and teahouses are especially relevant because they function not only as retail venues but also as everyday settings for social interaction, cultural practice, and identity expression [2,3]. Coffee shops are commonly associated with modern urban life, youth-oriented consumption, and experiences linked to external cultural influences, whereas teahouses are more closely connected to local everyday life, the continuity of traditional culture, and place-based social relations [2,4]. In contemporary Chinese cities, these two types of venues should not be understood simply as substitutes for one another. Rather, their coexistence, competition, and uneven spatial concentration provide an informative lens through which to examine the reorganization of urban cultural space [2–4]. Studying the spatiotemporal evolution of coffee–tea cultural spaces therefore offers a useful micro-scale perspective on broader processes of urban cultural change in China [1–4].

At the same time, the formation and evolution of coffee–tea cultural spaces do not occur in a homogeneous urban environment [1,2]. Cities differ in their historical development paths, degree of openness, industrial structure, tourism functions, educational resources, and local cultural traditions, and these differences may be reflected in the locational choices, spatial concentration, coexistence patterns, and directional changes observed in coffee shops and teahouses [1,4–6]. Within the Chinese urban system, coastal cities characterized by stronger external openness and tourism-oriented consumption may differ from inland cities marked by stronger local cultural continuity and everyday place-based social relations in the organization of consumption space and the evolution of coffee–tea spatial patterns [1,4,6,7]. A comparative perspective across contrasting urban contexts is therefore useful for identifying how different city settings are associated with different forms of cultural-space organization, coexistence, and transition [1,4–7].

The period from 2018 to 2024 also provides a useful temporal window for examining change around the COVID-19 period [8,9]. Existing research has shown that this period was associated with changes in offline consumption, mobility, commercial activity, and urban public life [8–11]. Because coffee shops and teahouses differ in consumption setting, customer base, locational dependence, and social function, their observed spatial trajectories may also have differed across this period [8,10,11]. In this study, the COVID-19 period is treated as an important temporal context rather than as an independently identified causal mechanism. This framing helps situate changes in coffee–tea cultural spaces within a broader period of disruption, adjustment, and recovery across contrasting urban settings [8–11].

Existing studies have examined coffee shops, teahouses, and other consumption and leisure spaces from the perspectives of social and cultural meaning, spatial distribution, and public life [1–7]. On the one hand, a growing body of work has addressed the agglomeration of consumption spaces, locational differences across coffee retail models, the social role of coffee shops as public and third places, and spatial inequalities in the availability of third places [1,2,5,6,12,13]. On the other hand, studies of teahouses, local public life, everyday social relations, and spatial organization have provided an important basis for understanding tea-related spaces in Chinese cities [4,7]. Research has also accumulated on commercial vulnerability, urban vibrancy recovery, changes in third places, and the wider urban implications of the COVID-19 period [8–11,14]. However, several gaps remain. First, coffee shops and teahouses have mostly been examined as separate business types, with limited attention to how their coexistence and relative spatial balance jointly structure coffee–tea cultural spaces [2,4–7,12,13]. Second, much of the existing literature focuses on a single city, a single business type, or a single time period, while comparative analyses across cities with contrasting historical and functional contexts remain limited [1,5–7,12,13]. Third, although the COVID-19 period has been widely recognized as an important context for changing urban consumption and public life, less attention has been paid to how coffee–tea cultural spaces followed different temporal pathways across cities before, during, and after this period [8–11,14]. A unified comparative framework based on long-term annual observations is therefore still needed to examine the evolution, coexistence patterns, and directional transitions of coffee–tea cultural spaces across different urban contexts [1–14].

Against this background, this study compares Qingdao and Jinan as two contrasting urban cases. Qingdao is more strongly associated with coastal openness, tourism-oriented consumption, and external cultural influences, whereas Jinan is more closely associated with local cultural continuity and the everyday social structure of an inland provincial capital. Using annual POI data on coffee shops and teahouses from 2018 to 2024, this study applies a unified 500 m × 500 m grid-based analytical framework to examine POI-observed coffee–tea cultural spaces in the two cities. The analysis combines Coffee Ratio (CR), Hybridization Index (HI), and Cultural Transition Intensity (CTI) with cultural zones, trajectory types, and functional-zone context to compare long-run composition, coexistence balance, and directional change. Specifically, the study addresses three questions: (1) how did coffee–tea cultural spaces in the two cities differ in overall temporal dynamics and long-run spatial structure during 2018–2024; (2) how did the two cities differ in directional change and temporal pathway structure across the study period, including the years overlapping with the COVID-19 period; and (3) how were long-run cultural zones and temporal trajectory types embedded in different urban functional contexts in the two cities?

## 2 Methods

### 2.1 Comparative study design and analytical framework

**Fig 1:**
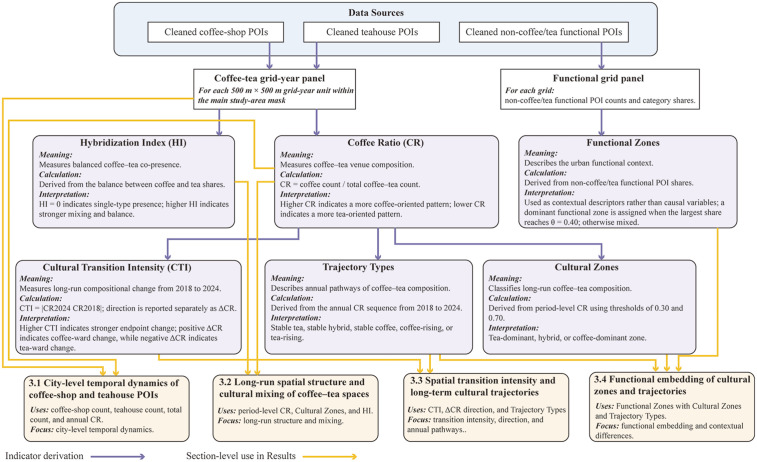
Research framework for the grid-based analysis of coffee–tea cultural spaces.

This study compared the spatial organization and temporal change of coffee shops and teahouses in Jinan and Qingdao under a unified analytical framework. The two cities were selected because they are major cities within the same provincial context, which improves baseline comparability in data source, policy environment, and regional setting. At the same time, the two cities differ in urban morphology, historical development, and functional orientation. Jinan represents a traditional inland historical city, whereas Qingdao reflects a coastal city with a modern colonial-port development history, a more tourism-oriented spatial structure, and stronger links to external exchange. Coffee shops and teahouses were treated as everyday consumption venues with relevance to third-place and everyday public-space research [15,16]. Annual POI data for 2018–2024 were aggregated to a fixed 500 m × 500 m grid, from which grid-year counts, long-run spatial indicators, endpoint-based change metrics, and temporal trajectory types were derived. The overall analytical workflow is summarized in Fig 1. Detailed implementation rules for data preprocessing, indicator construction, trajectory classification, and functional zoning are provided in S1–S4 Appendices, and sensitivity and supplementary robustness analyses are reported in S5 Appendix. Key indicators and analytical concepts used in the comparative analysis are summarized in Table 1.

**Table 1:** Key indicators and analytical concepts used in the comparative analysis of POI-observed coffee–tea cultural spaces in Jinan and Qingdao.

Indicator / concept	Cultural meaning (what it captures)	Operationalization in this study
**POI-observed coffee–tea cultural spaces**	The overarching analytical object of the study, referring to urban cultural spaces associated with coffee- and tea-related everyday consumption practices as observed through annual POI records.	In each study city, coffee shops and teahouses were identified from annual POI data and analyzed within a harmonized grid-based framework to examine their spatial distribution, coexistence balance, and temporal change.
**Coffee Ratio (CR)**	The relative dominance of coffee-oriented versus tea-oriented establishments within a grid cell.	For each grid-year, CR was calculated as the proportion of coffee shops among all coffee and tea establishments. Annual CR was used for temporal analyses, whereas period-aggregated CR (pooling counts across 2018–2024) was used for long-term spatial classification.
**Hybridization Index (HI)**	The degree of coexistence balance between coffee- and tea-related establishments within the same grid cell.	HI was calculated as a Shannon entropy-based index derived from the period-aggregated coffee–tea composition in each grid. Higher values show more balanced co-presence, whereas lower values show stronger dominance by one category.
**Cultural Transition Intensity (CTI)**	The magnitude of endpoint-based net change in coffee–tea composition between 2018 and 2024, with transition direction indicated by signed CR change.	For each grid with valid CR values in both 2018 and 2024, signed endpoint change was calculated as ΔCR = CR2024 − CR2018. Positive ΔCR indicates a coffee-ward transition, negative ΔCR indicates a tea-ward transition, and CTI was calculated as |ΔCR| to represent the magnitude of net change.
**Cultural zone**	The long-term spatial orientation of coffee–tea composition within a grid cell.	Based on period-aggregated CR, each grid was classified as tea-dominant, hybrid, or coffee-dominant using predefined threshold rules.
**Trajectory type**	The temporal pathway through which coffee–tea composition evolved within a grid cell during 2018–2024.	Using observed annual CR sequences from 2018 to 2024, eligible grids were assigned to one of five trajectory types using a slope-based rule. Annual CR slope was used to identify coffee-rising and tea-rising trajectories, whereas grids within the stable slope band were classified as stable tea, stable hybrid, or stable coffee according to mean annual CR.
**Functional zone**	The broader urban functional context associated with the distribution of coffee–tea cultural spaces.	Amap functional POIs, excluding coffee/tea and beverage-related categories, were aggregated into harmonized functional categories within each grid-year. Annual functional-zone labels were assigned using the dominant-share rule, and the period-level functional zone used in contextual comparisons was derived as the modal annual label among grids with sufficient valid functional-zone observations. Functional zones were used as contextual descriptors rather than causal explanatory variables.

Note: All indicators were implemented separately but identically in Jinan and Qingdao using the same harmonized POI classification system and fixed 500 m × 500 m grid. Cultural zones and trajectory types were derived from different temporal aggregation schemes to avoid circular definitions.

### 2.2 Study areas, POI data, and preprocessing

This study used two POI sources for different analytical purposes. Dianping POIs were used to identify coffee-shop and teahouse venues, whereas Amap (Gaode Map) POIs were used to construct the grid-level functional-zone context. The two sources were not treated as equivalent POI systems for direct comparison; rather, they served separate analytical roles within the same 500 m × 500 m grid framework.

Annual POI snapshots for 2018–2024 were organized by observation year. Dianping records provided the primary basis for measuring the observed presence of coffee-shop and teahouse venues, and each record contained a stable platform venue identifier, establishment name, address, platform category label, geographic coordinates, and observation year. Amap POIs were used to describe broader urban functional context and were not used to define the main coffee–tea venue indicators.

Because platform category taxonomies and labeling rules may vary across years, raw platform categories were harmonized year by year into stable analytical classifications. Dianping records were harmonized into two target venue classes: coffee shops, defined as venues whose primary business identity was coffee consumption, and teahouses, defined as venues centered on on-site tea drinking, tea-room consumption, traditional teahouses, tea clubs, tea ceremony spaces, and comparable Chinese categories such as 茶馆, 茶楼, 茶室, and 茶社. Milk-tea shops, fruit-tea shops, general beverage shops, dessert drink shops, restaurants with only incidental tea or coffee service, and ambiguous cafés without coffee-specific classification were excluded. Amap functional POIs were recoded into harmonized functional categories. Data cleaning was conducted primarily by POI identifier. Within-year duplicate screening used a 50–100 m proximity rule, and records with unresolved ID–address inconsistency were excluded from longitudinal linkage. All spatial data were transformed to WGS84 / UTM Zone 50N (EPSG:32650). Detailed data harmonization, ID cleaning, and spatial preprocessing procedures are provided in S1 Appendix.

### 2.3 Fixed grid framework and construction of city-specific study-area masks

**Fig 2:**
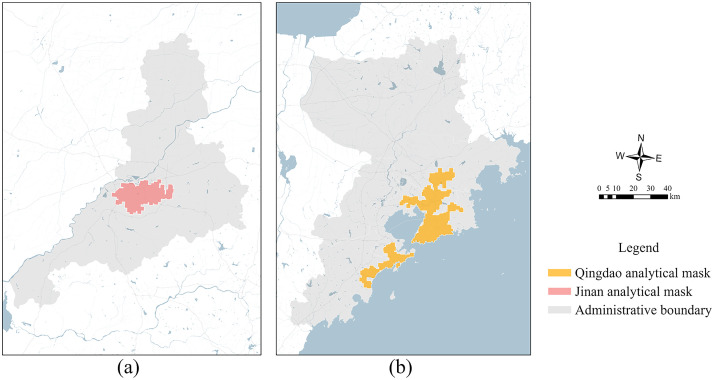
Final grid-based study-area masks for Jinan and Qingdao. (a) Final study-area mask for Jinan. (b) Final study-area mask for Qingdao. Note: Administrative boundaries and final analytical masks are shown for the two study cities. The masks were constructed using the rule-based grid-retention procedure described in the Methods and S1 Appendix.

A regular 500 m × 500 m grid in WGS84 / UTM Zone 50N (EPSG:32650) was used as the basic spatial unit in both cities. City-specific study-area masks were delineated using the same rule-based procedure. A grid was retained as a core activity grid if it had a cumulative coffee + tea count of at least 4 during 2018–2024 and observed activity in at least 4 distinct years. Core grids were then expanded by two rounds of queen adjacency. Expanded grids were grouped into contiguous clusters using edge-based connectivity only, excluding corner-only contact. Clusters smaller than 120 km^2^ were removed, and enclosed interior holes were filled when their area was 10 km^2^ or less. The resulting final grid-based study-area masks for Jinan and Qingdao are shown in Fig 2. Detailed procedures for grid construction, activity filtering, adjacency expansion, cluster refinement, and hole filling are provided in S1 Appendix. Because the main masks were derived from observed coffee–tea venue activity, alternative mask definitions were evaluated in the sensitivity analysis described in Section 2.8 and S5 Appendix.

### 2.4 Grid-year dataset construction

Within each final study-area mask, coffee-shop and teahouse POIs were assigned to grid cells and aggregated by grid and year for 2018–2024. The resulting panel dataset recorded, at minimum, grid ID, year, coffee-shop count, and teahouse count. No temporal interpolation was applied. Years without valid observations were retained as non-observed rather than imputed. For long-run analyses, annual counts were pooled across 2018–2024 to obtain period-aggregated coffee and tea counts for each grid. Detailed rules for grid-year panel construction are provided in S1 Appendix, and subsequent indicator calculations based on this panel are described in S2 Appendix.

### 2.5 Cultural indicators and spatial cultural zoning

Three grid-level indicators were constructed from the harmonized coffee and tea counts: Coffee Ratio (CR), Hybridization Index (HI), and Cultural Transition Intensity (CTI). Annual CR was calculated as the proportion of coffee shops among all coffee and tea establishments in a grid-year. Period-aggregated CR was calculated from pooled counts over 2018–2024 and was used for long-run spatial classification. HI was calculated as a Shannon entropy–based measure of coffee–tea balance [17]. For endpoint-based change analysis, signed endpoint change was first calculated as ΔCR = CR2024 − CR2018 for grids with valid annual CR values in both endpoint years. Positive ΔCR indicates coffee-ward change, whereas negative ΔCR indicates tea-ward change. CTI was then defined as |ΔCR| and used to represent the magnitude of endpoint-based net change. Based on period-aggregated CR, each grid was classified as tea-dominant (CR ≤ 0.30), hybrid (0.30 < CR < 0.70), or coffee-dominant (CR ≥ 0.70). Full formulas, thresholds, and implementation rules are provided in S2 Appendix. Alternative CR threshold settings and endpoint-window diagnostics for signed endpoint change were examined as robustness checks, as described in Section 2.8 and S5 Appendix.

### 2.6 Cultural trajectory identification

Temporal trajectory types were identified from observed annual CR sequences during 2018–2024. A grid was eligible for trajectory classification only if it had valid coffee + tea observations in at least four years and a cumulative coffee + tea count of at least four across the study period. No temporal interpolation was applied to missing grid-year observations.

For each eligible grid, a simple linear slope of annual CR against year was calculated using valid observed annual CR values. The annual CR slope was evaluated first to identify directional trajectories. A grid was classified as coffee rising when the slope was greater than 0.05 and as tea rising when the slope was less than −0.05. Grids with slopes within the stable band from −0.05 to 0.05 were then classified using mean annual CR. Within this stable band, grids with mean annual CR at or below 0.30 were classified as stable tea, grids with mean annual CR at or above 0.70 were classified as stable coffee, and the remaining grids were classified as stable hybrid. Detailed eligibility criteria, slope calculation, stable-band rules, and interpretation boundaries are provided in S3 Appendix.

### 2.7 Functional zoning and contextual analyses

POI-based functional zoning has been widely used to represent urban activity context and mixed-use structure [18–24]. In this study, functional zones were used as contextual descriptors rather than causal explanatory variables. A separate functional-context layer was constructed from harmonized Amap functional POIs aggregated to the same 500 m × 500 m grid within the final study-area masks. Coffee-shop, teahouse, milk-tea, fruit-tea, general beverage, and dessert drink categories were excluded from the Amap-based functional-zone classification to reduce mechanical overlap with the Dianping-based coffee–tea indicators.

For each grid-year, category-specific functional POI counts were converted to within-grid proportions. A dominant functional-zone label was assigned when the largest functional-category share reached the main dominant-share threshold of 0.40; otherwise, the grid was classified as Mixed. For period-level contextual comparisons, a grid was retained only when it had at least four non-missing annual functional-zone labels during 2018–2024, and its final period-level functional zone was assigned as the modal annual functional-zone label across valid years. Grids with insufficient functional information were excluded from functional-zone comparisons. Detailed functional-category definitions, dominant-share rules, annual-to-period aggregation procedures, and sensitivity analyses are provided in S4 Appendix.

### 2.8 Sensitivity and supplementary robustness analyses

First, because the main study-area masks were derived from observed coffee–tea venue activity, we compared the main analytical mask with two alternative mask definitions. The expanded active mask included grids with coffee-shop or teahouse activity in any year during 2018–2024, whereas the one-ring expanded mask was constructed by expanding the main analytical mask outward by one Queen-contiguity grid ring. This check was used to evaluate whether the main city-level temporal contrasts and spatial patterns were sensitive to the study-area delineation procedure.

Second, because the classification of cultural zones and stable trajectory categories depends on CR thresholds, we tested alternative threshold pairs of 0.25/0.75 and 0.35/0.65 against the main 0.30/0.70 specification. These checks evaluated whether the cultural-zone composition and five-category trajectory classifications were robust to reasonable changes in threshold settings.

Third, because CTI is an endpoint-based magnitude indicator derived from signed endpoint change, we supplemented the main 2018–2024 endpoint comparison with annual dynamics, adjacent-year cultural-zone transition matrices, short-term reversal checks, and alternative endpoint-window diagnostics. These analyses were used to clarify whether endpoint-based CTI patterns were consistent with broader year-by-year temporal evidence.

Fourth, because grid-level observations may be spatially structured, we conducted global Moran’s I tests for the main cultural indicators using Queen contiguity weights. These tests were used to assess spatial autocorrelation in annual CR and HI, period-level CR and HI, and endpoint CTI.

Finally, because functional-zone classification depends on the dominant-share threshold, we tested alternative functional-zone thresholds of 0.35 and 0.45 against the main 0.40 specification. The threshold-specific functional-zone layers were then compared with cultural zones and trajectory types to evaluate whether the functional-context associations remained stable under alternative classification rules. Detailed procedures and results for these sensitivity and robustness analyses are reported in S5 Appendix.

## 3 Results

### 3.1 City-level temporal dynamics of coffee-shop and teahouse POIs in Jinan and Qingdao

**Fig 3:**
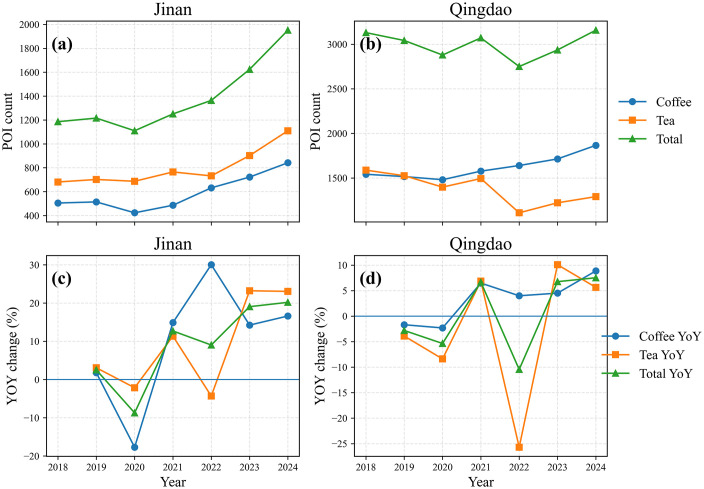
Annual totals and year-on-year changes in coffee-shop and teahouse POIs within the final study-area masks of Jinan and Qingdao, 2018–2024. (a) Annual numbers of coffee-shop, teahouse, and total POIs in Jinan. (b) Annual numbers of coffee-shop, teahouse, and total POIs in Qingdao. (c) Year-on-year changes in coffee-shop, teahouse, and total POIs in Jinan. (d) Year-on-year changes in coffee-shop, teahouse, and total POIs in Qingdao. Note: All counts were calculated within the final study-area mask of each city and should not be interpreted as municipality-wide totals. “Total” refers to the combined number of coffee-shop and teahouse POIs observed in a given year. Year-on-year (YoY) change was calculated as the percentage change relative to the previous year; therefore, YoY values are shown for 2019–2024 only. The figure is based on the city-level annual summary data reported in S2 Table.

Fig 3 and S2 Table show different city-level temporal patterns in Jinan and Qingdao during 2018–2024. In Jinan, the total number of coffee-shop and teahouse POIs increased from 1,186 in 2018–1,952 in 2024, an overall increase of 64.6% (Fig 3a; S2 Table). Coffee-shop POIs increased from 505 to 842 (+66.7%), and teahouse POIs increased from 681 to 1,110 (+63.0%), with teahouse POIs remaining more numerous throughout the study period. A temporary decline occurred in 2020, when the total fell from 1,216 in 2019–1,110 (–8.7%). This decline was larger for coffee-shop POIs, which fell from 514 to 423 (–17.7%), than for teahouse POIs, which decreased from 702 to 687 (–2.1%) (Fig 3c; S2 Table). After 2020, the total number of POIs increased by 12.7% in 2021, 9.0% in 2022, 19.1% in 2023, and 20.2% in 2024 (Fig 3c; S2 Table). The number of active grids also increased from 426 in 2018–585 in 2024 (+37.3%) (S2 Table). Over the same period, coffee share changed only slightly, from 42.6% in 2018 to 43.1% in 2024, although it reached 46.3% in 2022 (S2 Table).

Qingdao showed a different pattern. The total number of coffee-shop and teahouse POIs changed only slightly, from 3,130 in 2018–3,157 in 2024 (+0.9%) (Fig 3b; S2 Table). Within this near-stable total, coffee-shop POIs increased from 1,542–1,866 (+21.0%), whereas teahouse POIs declined from 1,588–1,291 (–18.7%). Coffee share increased from 49.3% in 2018 to 59.1% in 2024, while tea share declined from 50.7% to 40.9% (S2 Table). From 2018 to 2020, both categories declined and the total fell from 3,130–2,879 (–8.0%). In 2020, coffee-shop POIs decreased by 2.3% year on year and teahouse POIs by 8.4% (Fig 3d; S2 Table). In 2022, coffee-shop POIs increased from 1,577–1,640 (+4.0%), whereas teahouse POIs dropped from 1,494–1,110 (–25.7%), bringing the total down to 2,750, the lowest value in the series (Fig 3b, 3d; S2 Table). In 2023–2024, both categories increased again, but coffee-shop POIs remained more numerous than teahouse POIs.

Across the two cities, Jinan showed larger net growth in total POIs and active grids, whereas Qingdao showed a larger shift in internal composition. Between 2018 and 2024, Jinan increased by 766 POIs and 159 active grids, compared with 27 POIs and 61 active grids in Qingdao (S2 Table). Because the final study-area masks differ in spatial extent, these totals refer to the retained study areas rather than municipality-wide counts.

### 3.2 Long-run spatial structure and cultural mixing of coffee–tea spaces

**Fig 4:**
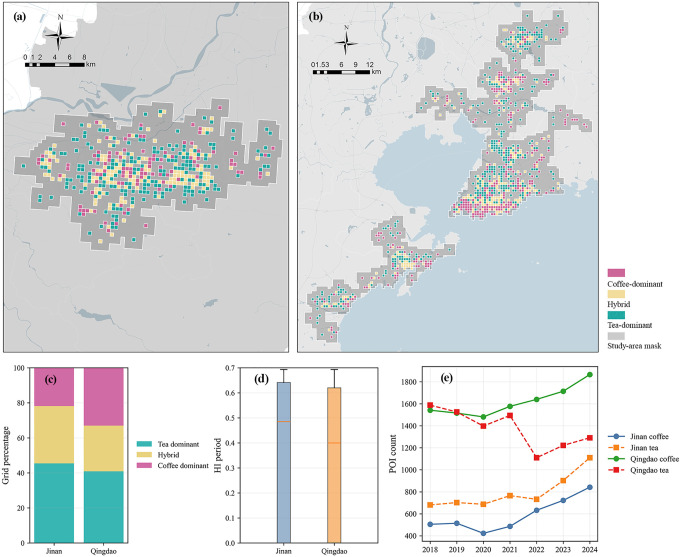
Long-run spatial structure and cultural mixing of coffee–tea spaces in Jinan and Qingdao. (a) Long-run CR-based cultural zones in Jinan. (b) Long-run CR-based cultural zones in Qingdao. (c) Composition of long-run CR-based cultural zones in Jinan and Qingdao. (d) Distribution of the long-run hybridization index (HI) in Jinan and Qingdao. (e) Annual numbers of coffee-shop and teahouse POIs within the final study-area masks of Jinan and Qingdao, 2018–2024. Note: Panel (e) is included as city-level structural context for the long-run spatial comparisons shown in panels (a–d). Long-run cultural zones were derived from period-aggregated coffee and tea counts over 2018–2024 using the long-run coffee ratio (CR). Tea-dominant grids were defined as CR ≤ 0.30, hybrid grids as 0.30 < CR < 0.70, and coffee-dominant grids as CR ≥ 0.70. HI was calculated from the same period-aggregated coffee and tea counts. Panels (a–d) were restricted to valid grids retained for the long-run CR/HI analysis.

Fig 4 shows clear intercity differences in long-run CR-based zoning and HI distribution. As structural context, Fig 4e summarizes the annual coffee-shop and teahouse POI counts described in Section 3.1. In Jinan, 458 valid grids were identified for the long-run CR/HI analysis, including 208 tea-dominant grids (45.41%), 150 hybrid grids (32.75%), and 100 coffee-dominant grids (21.83%) (Fig 4a, 4c). In Qingdao, 964 valid grids were identified, including 394 tea-dominant grids (40.87%), 252 hybrid grids (26.14%), and 318 coffee-dominant grids (32.99%) (Fig 4b, 4c). Thus, tea-dominant grids accounted for the largest share in both cities, but Qingdao had a substantially higher share of coffee-dominant grids than Jinan (32.99% vs. 21.83%) (Fig 4a–4c).

The mapped CR patterns also differed between the two cities. In Jinan, tea-dominant grids formed a broad background in the core built-up area, with hybrid and coffee-dominant grids appearing as clustered embedded patches (Fig 4a). In Qingdao, coffee-dominant grids formed multiple prominent clusters, especially along the coastal urban belt and major coastal activity areas, with tea-dominant and hybrid grids distributed between or around them (Fig 4b).

The long-run HI distribution also differed between the two cities (Fig 4d). The median HI was 0.485 in Jinan and 0.400 in Qingdao, and the mean HI was 0.370 in Jinan and 0.329 in Qingdao. Grids with HI = 0 accounted for 34.06% of valid grids in Jinan and 39.63% in Qingdao. Highly mixed grids with HI ≥ 0.60 accounted for 34.93% in Jinan and 27.90% in Qingdao. Spatially, hybrid zones in Fig 4a, 4b and high-HI grids in S1a, S1b Fig were concentrated mainly in urban cores or major activity clusters in both cities, whereas peripheral areas more often showed lower mixing or single-dominant structures.

The supplementary stratified analysis by period-aggregated activity volume showed the same direction of difference (S1c, S1d Fig). In the lowest-activity stratum (1–4), the median HI was 0 in both cities, and grids with HI = 0 accounted for 80.0% in Jinan and 91.5% in Qingdao. The share of highly mixed grids (HI ≥ 0.60) increased across activity strata, from 12.5%, 27.0%, and 44.6% in Jinan and from 3.2%, 21.3%, and 36.6% in Qingdao. In the highest-activity stratum (>10), the median HI remained slightly higher in Jinan than in Qingdao (0.572 vs. 0.540) (S1c, S1d Fig).

Overall, Qingdao had a larger number of valid grids and a higher coffee-dominant share than Jinan, whereas Jinan had a larger share of highly mixed grids (Fig 4; S1 Fig).

### 3.3 Spatial transition intensity and long-term cultural trajectories

**Fig 5:**
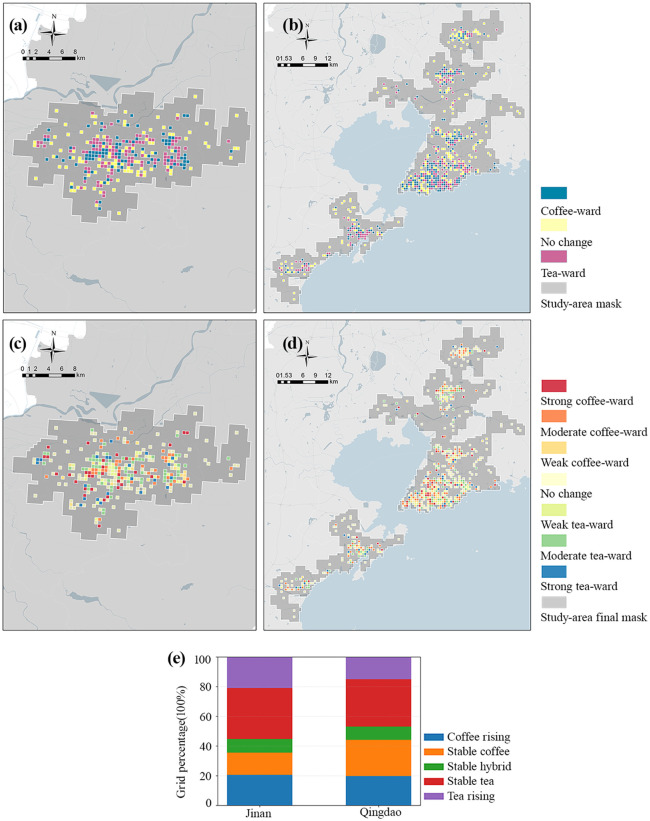
Spatial patterns of coffee–tea transition direction, CTI classes, and long-term trajectory composition in Jinan and Qingdao, 2018–2024. (a) Spatial distribution of transition direction in Jinan. (b) Spatial distribution of transition direction in Qingdao. (c) Spatial distribution of directional CTI classes in Jinan. (d) Spatial distribution of directional CTI classes in Qingdao. (e) Composition of long-term cultural trajectory types in Jinan and Qingdao. Note: Panels (a) and (b) show whether each grid included in the CTI analysis shifted in a coffee-ward direction, remained unchanged, or shifted in a tea-ward direction between 2018 and 2024. Panels (c) and (d) classify the magnitude of endpoint-based net change into directional CTI classes. Panel (e) summarizes the percentage composition of the five trajectory types derived from annual CR sequences during 2018–2024. CTI and trajectory type were derived from different temporal constructions and should be interpreted as complementary rather than interchangeable measures of change. Panels (a–d) and panel (e) therefore use different eligible grid samples.

Fig 5 and S3–S6 Tables show intercity differences in transition direction, transition intensity, cultural-zone conversion, and long-term trajectory composition. In the CTI analysis, Jinan included 299 comparable grids, of which 107 (35.8%) showed a tea-ward transition, 98 (32.8%) showed no net transition, and 94 (31.4%) showed a coffee-ward transition (Fig 5a; S3 Table). Qingdao included 650 comparable grids, of which 247 (38.0%) showed no net transition, 217 (33.4%) showed a coffee-ward transition, and 186 (28.6%) showed a tea-ward transition (Fig 5b; S3 Table).

Directional CTI classes showed that no-change grids were the largest single class in both cities, while the non-zero transition classes differed between the two cities (Fig 5c, 5d; S4 Table). In Jinan, strong coffee-ward transition accounted for 48 grids (16.1%), slightly exceeding strong tea-ward transition at 47 grids (15.7%). Moderate tea-ward transition accounted for 35 grids (11.7%), followed by weak tea-ward transition at 25 grids (8.4%), moderate coffee-ward transition at 24 grids (8.0%), and weak coffee-ward transition at 22 grids (7.4%) (Fig 5c; S4 Table). In Qingdao, strong coffee-ward transition was the largest non-zero class, accounting for 91 grids (14.0%), followed by strong tea-ward transition at 69 grids (10.6%), moderate coffee-ward transition at 67 grids (10.3%), weak tea-ward transition at 64 grids (9.8%), weak coffee-ward transition at 59 grids (9.1%), and moderate tea-ward transition at 53 grids (8.2%) (Fig 5d; S4 Table). Spatially, transition grids in Jinan were concentrated mainly in the contiguous built-up core, whereas in Qingdao they were distributed more strongly along the southern coastal belt and major urban corridors (Fig 5a–5d).

The cultural-zone transition matrix also differed between the two cities (S5 Table; S2 Fig). In Jinan, 150 grids remained in the same cultural zone between 2018 and 2024, accounting for 50.2% of comparable grids. In Qingdao, 383 grids remained in the same zone, accounting for 58.9% (S5 Table; S2 Fig). In Jinan, among tea-dominant grids in 2018, 58.8% remained tea-dominant in 2024, 20.6% shifted to hybrid, and 20.6% shifted to coffee-dominant. Among coffee-dominant grids, 45.1% remained coffee-dominant, 34.1% shifted to hybrid, and 20.9% shifted to tea-dominant. Among hybrid grids, 45.8% shifted to tea-dominant, 40.3% remained hybrid, and 13.9% shifted to coffee-dominant. In Qingdao, 65.2% of tea-dominant grids remained tea-dominant and 64.9% of coffee-dominant grids remained coffee-dominant. Among hybrid grids, 41.3% shifted to coffee-dominant, 37.1% remained hybrid, and 21.7% shifted to tea-dominant (S5 Table; S2 Fig).

Trajectory composition showed further differences (Fig 5e; S6 Table). In the trajectory analysis, Jinan included 458 grids and Qingdao 964. In both cities, stable tea was the largest category, accounting for 157 grids (34.3%) in Jinan and 307 grids (31.8%) in Qingdao. Stable coffee accounted for 69 grids (15.1%) in Jinan and 236 grids (24.5%) in Qingdao. Tea rising accounted for 96 grids (21.0%) in Jinan and 145 grids (15.0%) in Qingdao. Coffee rising accounted for 94 grids (20.5%) in Jinan and 190 grids (19.7%) in Qingdao. Stable hybrid accounted for 9.2% in Jinan and 8.9% in Qingdao (Fig 5e; S6 Table).

The supplementary endpoint-sensitivity analysis showed broad robustness of the main signed endpoint-change patterns (S3 Fig; S9 Table). Using 2023 as the alternative endpoint, Pearson correlations between signed endpoint changes for 2018 → 2024 and 2018 → 2023 were 0.947 in Jinan and 0.891 in Qingdao, with sign agreement of 90.2% and 86.7%, respectively. Using 2022 as the alternative endpoint, the corresponding correlations were 0.842 and 0.757, with sign agreement of 78.0% and 78.6%. Direction-reversal rates ranged from 3.5% to 7.8%, and hotspot overlap was higher for 2023 than for 2022 (S3 Fig; S9 Table).

### 3.4 Functional embedding of cultural zones and trajectories

**Fig 6:**
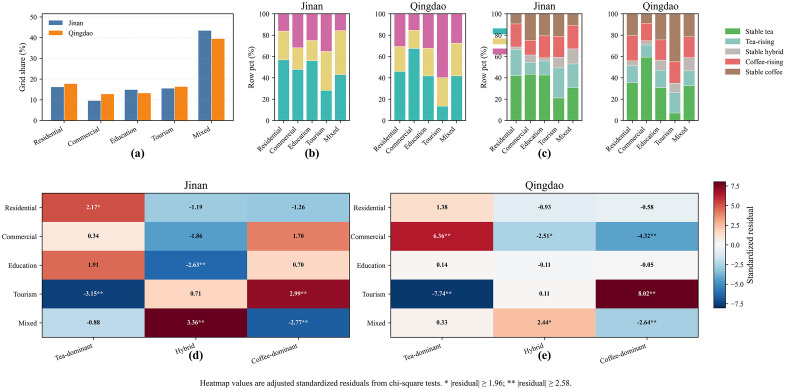
Functional-zone composition and its associations with cultural zones and trajectory types in Jinan and Qingdao. (a) Functional-zone composition in Jinan and Qingdao. (b) Functional zone × cultural zone in Jinan and Qingdao. (c) Functional zone × trajectory type in Jinan and Qingdao. (d) Adjusted standardized residuals for functional zone × cultural zone in Jinan. (e) Adjusted standardized residuals for functional zone × cultural zone in Qingdao. Note: Panel (a) shows the percentage share of grids in each functional-zone category in Jinan and Qingdao. Panels (b) and (c) show row percentages, indicating the proportional composition of cultural zones and trajectory types within each functional-zone category. Panels (d) and (e) show adjusted standardized residuals from chi-square tests for the association between functional zones and cultural zones. Positive residuals indicate combinations occurring more frequently than expected under independence, whereas negative residuals indicate combinations occurring less frequently than expected. * |residual| ≥ 1.96; ** |residual| ≥ 2.58. Trajectory-type residuals are shown separately in S4 Fig. All panels were restricted to grids with valid functional-zone classification; therefore, the sample size may differ slightly from the full trajectory-classification sample reported in S6 Table.

Fig 6 and S7–S8 Tables show significant associations between functional zones and both cultural zones and trajectory types in the two cities. At the aggregate level, both study areas were dominated by Mixed grids, but their functional-zone compositions differed (Fig 6a; S7 Table). In Jinan, Mixed was the largest category (43.5%), followed by Residential (16.3%), Tourism (15.6%), Education (14.9%), and Commercial (9.7%). In Qingdao, Mixed was also the largest category (39.6%), followed by Residential (17.8%), Tourism (16.4%), Education (13.3%), and Commercial (12.9%) (Fig 6a; S7 Table).

For cultural zones, the functional zone × cultural zone association was significant in both cities (S8 Table). In Jinan, the association was weaker (χ² = 32.857, df = 8, p < 0.001, Cramér’s V = 0.190). Residential, Commercial, and Education were all dominated by tea-dominant grids, at 56.8%, 47.7%, and 55.9%, respectively. Mixed had the highest share of hybrid grids among the functional zones (41.4%). Tourism showed relatively high shares of hybrid grids (36.6%) and coffee-dominant grids (35.2%), compared with 28.2% tea-dominant grids (Fig 6b; S7 Table). Adjusted standardized residuals showed positive deviations for Mixed × hybrid (3.36) and Tourism × coffee-dominant (2.99), and negative deviations for Tourism × tea-dominant (–3.15), Education × hybrid (–2.63), and Mixed × coffee-dominant (–2.77) (Fig 6d).

In Qingdao, the association was stronger (χ² = 108.707, df = 8, p < 0.001, Cramér’s V = 0.239; S8 Table). Tourism was strongly coffee-dominant, with 59.9% coffee-dominant grids and 13.4% tea-dominant grids. Commercial was strongly tea-dominant, with 67.5% tea-dominant grids and 15.4% coffee-dominant grids (Fig 6b; S7 Table). Adjusted standardized residuals were highest for Tourism × coffee-dominant (8.02) and Commercial × tea-dominant (6.36), and lowest for Tourism × tea-dominant (–7.74) and Commercial × coffee-dominant (–4.32) (Fig 6e).

For trajectory types, the functional zone × trajectory type association was also significant in both cities (S8 Table). In Jinan, the association was weaker (χ² = 37.499, df = 16, p = 0.002, Cramér’s V = 0.144). Stable tea was the most common trajectory in Residential (41.9%), Commercial (43.2%), and Education (42.6%). Mixed had the highest share of stable hybrid trajectories among the functional zones (14.1%). Tourism showed relatively similar shares of tea-rising (28.2%), stable tea (21.1%), and stable coffee (21.1%) trajectories (Fig 6c; S7 Table). Adjusted residuals in S4a Fig showed the strongest positive deviation for Mixed × stable hybrid (3.18), together with negative deviations for Tourism × stable tea (–2.50) and Mixed × stable coffee (–2.28).

In Qingdao, the association was stronger (χ² = 118.565, df = 16, p < 0.001, Cramér’s V = 0.176; S8 Table). Tourism had the highest share of stable coffee trajectories (44.6%) and a low share of stable tea trajectories (7.0%), whereas Commercial had the highest share of stable tea trajectories (59.3%) and a low share of stable coffee trajectories (8.9%) (Fig 6c; S7 Table). Adjusted residuals in S4b Fig showed the strongest positive deviations for Tourism × stable coffee (6.66), Commercial × stable tea (6.95), and Mixed × stable hybrid (3.00), together with strong negative deviations for Tourism × stable tea (–7.35), Commercial × stable coffee (–4.16), Residential × stable hybrid (–2.16), and Commercial × stable hybrid (–2.05) (S4b Fig).

### 3.5 Robustness checks

The supplementary robustness analyses showed that the main intercity contrasts were not driven solely by specific analytical choices. The study-area mask sensitivity analysis showed that the main differences between Jinan and Qingdao remained broadly consistent under alternative mask definitions, including the main analytical mask, the expanded active mask, and the one-ring expanded mask (S10 Table). The CR-threshold and trajectory-classification sensitivity analyses also showed that the relative contrast between Jinan’s stronger tea-dominant and mixed structure and Qingdao’s stronger coffee-dominant tendency remained similar under alternative cutoff settings S11–S12 Table). Functional-zone threshold sensitivity checks further showed that the main functional-embedding patterns remained consistent, although the size of the Mixed category changed with the dominant-share threshold (S15 Table; S5 Appendix).

The endpoint-sensitivity analysis indicated that signed endpoint-change patterns were more consistent when 2023 was used as the alternative endpoint than when 2022 was used, while direction-reversal rates remained limited (S3 Fig; S9 Table). Spatial autocorrelation checks showed that several indicators had significant spatial structure, supporting the need to interpret the results as spatially structured descriptive patterns rather than independent grid-level observations (S14 Table). Overall, these checks supported the robustness of the main descriptive conclusions while reinforcing the need for cautious interpretation of POI-based spatial associations. An overall summary of the robustness findings is provided in S13 Table.

## 4 Discussion

### 4.1 Beyond replacement: Differentiated restructuring of POI-observed coffee–tea venue patterns

In recent years, related studies have largely approached the issue from the perspective of coffee growth, emphasizing the links between coffee shops and urban vitality, concentration in core areas, innovation, and urban renewal [25–27]. Within this research orientation, coffee expansion is often more readily interpreted as a more dominant form of change in urban consumption space [25,26]. By contrast, the present study does not support understanding changes in POI-observed coffee–tea venue patterns as a simple linear substitution between coffee and tea. Jinan exhibited clear overall expansion during 2018–2024, yet its coffee share increased only slightly from 42.6% to 43.1%. Qingdao, by comparison, showed only limited overall growth, but experienced marked internal restructuring, with coffee share rising from 49.3% to 59.1%. At the same time, both cities retained a substantial proportion of hybrid grids, indicating that the growth of coffee spaces did not correspond to the synchronous retreat of tea spaces across the whole urban area. Taken together, the comparative results from the two cities therefore support interpreting this process as a form of restructuring characterized by local variation and spatial unevenness, rather than simple replacement.

Another strand of recent research has approached coffee shops and teahouses more from the perspective of “third places,” everyday sociality, and emotional attachment [2,28]. On the one hand, this literature highlights the social, leisure, and symbolic functions of coffee shops [2]. On the other hand, it also suggests that teahouses have not automatically lost their practical significance with the rise of new consumption spaces, but continue to support everyday interaction and locally embedded social ties [28]. However, these studies have mostly focused on a single business type or a single type of venue, and have less often asked what mixed zones actually mean when coffee and tea coexist within the same urban spatial system. In contrast, the results of this study show that hybrid zones are neither marginal remnants nor negligible transitional noise. In both cities, hybrid grids account for a substantial share, and grids with high levels of mixing are concentrated mainly in urban cores or major activity areas, indicating that the coexistence of coffee and tea constitutes a substantive organizational form of POI-observed everyday consumption venues. At the same time, the share of stable hybrid trajectories remains low in both cities, suggesting that highly balanced and long-term stable mixed states are not widespread. Hybrid zones are therefore better understood as a form of spatial co-presence under ongoing adjustment, rather than as a static, homogeneous, and ultimately stable intermediate state.

Overall, the present study does not support understanding coffee–tea venue change in the two cities as a one-line process in which the “new” replaces the “old.” Instead, the comparison between Jinan and Qingdao shows that this process simultaneously involves overall expansion, selective concentration, persistent coexistence, and uneven directional shifts. In this sense, differentiated restructuring provides a more accurate summary than simple replacement for the observed spatial-temporal organization of coffee–tea venues revealed in this study.

### 4.2 Divergent city-specific pathways in Jinan and Qingdao

Recent research on urban consumption-space change has, on the one hand, emphasized that different cities do not necessarily follow the same path of restructuring and should therefore be understood through the lens of place specificity and comparative urbanism [29]. On the other hand, a growing body of work has shown that coffee shops are more closely associated with urban vitality, innovation, and concentration in core urban areas [25,26]. Taken together, these studies suggest that the expansion of new consumption spaces is not merely a matter of numerical growth, but may take the form of different types of spatial restructuring in different cities. The results of the present study generally support this broader view, but further show that the difference between Jinan and Qingdao lies not primarily in whether change occurred, but in how it unfolded. Under the same analytical framework, Jinan was characterized more by internal adjustment on the basis of overall expansion, whereas Qingdao was characterized more by directional compositional restructuring under conditions of relative stability in total scale. From annual change to long-run spatial structure, and from transition direction to trajectory composition, this divergence is highly consistent. The comparative evidence therefore suggests that the two cities did not evolve along a single pathway of coffee–tea venue change, but rather exhibited different modes of restructuring under a shared analytical design.

In the case of Jinan, the observed pattern is better described as bidirectional adjustment and rebalancing. Recent studies of historic districts, everyday urban environments, and the third-place functions of teahouses suggest that, in urban contexts with stronger historical continuity and more routinized everyday social life, traditional consumption spaces and everyday interaction settings tend to show greater resilience [28,30]. This interpretation is broadly consistent with the present findings. During the study period, Jinan experienced more pronounced overall expansion: both the total number of coffee-shop and teahouse POIs and the number of active grids increased substantially, whereas the change in coffee share between the beginning and end of the period remained limited. At the same time, Jinan retained a stronger tea-oriented structural base, with tea-dominant grids accounting for the largest share, more continuous high-HI spaces in the core built-up area, and hybrid grids more often shifting back toward tea-dominant rather than moving primarily toward coffee-dominant. In other words, change in Jinan was not mainly expressed as sustained replacement in a single direction, but rather as incremental expansion, persistent mixing, and bidirectional adjustment on the basis of an existing tea-oriented structure. In addition, studies of urban consumption-space change before and after COVID-19 have shown that the pandemic often introduced phased disruption rather than uniform long-term reordering [31]. This broader background is consistent with the short-term disturbance around 2020 and subsequent recovery observed in Jinan, although the present study does not identify the independent causal effect of the pandemic. Overall, it is therefore more appropriate to characterize Jinan as a case of bidirectional adjustment and rebalancing within a more continuous everyday urban setting than as a straightforward coffee-oriented transition.

By contrast, Qingdao is better characterized as a clearer case of coffee-oriented restructuring. Recent research on coffee spaces, urban vitality, and catering-space change under public crises suggests that coffee-oriented consumption spaces are more likely to be concentrated in highly visible, high-vitality, and selectively reorganized urban environments [25,26], and that pandemic-related disruptions in catering space often unfold in phased and spatially uneven ways [31,32]. These perspectives are broadly consistent with the present findings for Qingdao. Between 2018 and 2024, Qingdao showed only limited change in total scale, but coffee share increased over the study period, indicating that the key process was not overall expansion itself, but directional restructuring of coffee–tea composition. At the same time, Qingdao had a higher proportion of coffee-dominant grids than Jinan, more prominent coffee clustering along the coastal urban belt and major activity corridors, more frequent transition of hybrid grids toward coffee dominance, and a substantially higher proportion of stable coffee trajectories. These patterns indicate that coffee-oriented change in Qingdao was not limited to a single-year fluctuation. Qingdao also experienced phased adjustment around 2020–2022, especially with the sharper decline of teahouses in 2022. However, rather than attributing these changes directly to tourism conditions, external exchange, or pandemic shocks alone, the present study interprets them more cautiously as restructuring patterns that are consistent with Qingdao’s broader urban context as a coastal city, shaped by a modern colonial-port development history, stronger tourism orientation, and a more concentrated coastal activity structure. In this sense, what Qingdao exhibits is not simple expansion, but clearer coffee-oriented restructuring under conditions of relative stability in total scale.

### 4.3 Functional embedding as contextual association

Recent studies increasingly suggest that urban consumption spaces do not diffuse evenly across the city, but are instead associated with commercial, residential, workplace, accessibility, and mixed-use conditions; likewise, third places and composite consumption settings are more likely to emerge in dense, highly accessible, and functionally mixed areas [33–35]. The present study generally supports this view and further shows that the observed coffee–tea venue patterns were not evenly distributed across the urban area, but were associated with different functional-zone contexts. Both the functional zone × cultural zone and functional zone × trajectory type associations were significant in the two cities, with stronger effect sizes in Qingdao. At the same time, high-HI mixed areas were concentrated mainly in urban cores or major activity areas, further indicating that intense coffee–tea coexistence was not a random overlay, but was more often observed in spatial settings characterized by greater functional complexity, stronger accessibility, and denser activity concentration. One possible interpretation is that different functional settings correspond to different activity flows, rhythms of stay, and consumption scenarios, which may help explain the observed differences in coffee–tea venue composition.

More specifically, existing studies also suggest that the relationship between consumption forms and functional settings is not a simple one-to-one correspondence. Even within the coffee sector, different retail models may display markedly different locational preferences, while some traditional cultural spaces are able to persist under conditions of modern consumption and tourism not because they stand outside contemporary urban change, but because they remain deeply embedded in local ways of life [5,26,36]. The present study both supports and further refines this general view. In Jinan, the relationship between mixed-use areas and cultural hybridity was more pronounced, suggesting that coffee–tea coexistence there was more likely to occur within composite everyday urban settings. By contrast, Qingdao showed a clearer correspondence between tourism and coffee-dominant or stable-coffee patterns, alongside a stronger correspondence between commercial areas and tea-dominant or stable-tea patterns, indicating that cultural differentiation there was more explicitly associated with the consumption scenarios represented by different functional zones. Taken together, these findings suggest that the spatial unevenness of POI-observed coffee–tea venue restructuring was not simply a surface expression of consumer preference differences, but also reflected associations between distinct cultural business forms and different urban functional settings.

This correspondence, however, should not be interpreted as evidence of a single deterministic land-use mechanism. In this study, functional zones were used as contextual descriptors rather than causal explanatory variables, and coffee-shop, teahouse, and beverage-related categories were excluded from the functional-zone classification to reduce mechanical overlap with the coffee–tea indicators. A more cautious interpretation is that differences in spatial structure, tourism concentration, historical context, and everyday sociality may condition how similar functional settings are associated with different coffee–tea venue patterns in the two cities.

### 4.4 Implications for interpreting everyday consumption-venue restructuring

From a broader theoretical perspective, this study suggests that changes in everyday consumption venues should not be understood as a single linear pathway in which the “modern” replaces the “traditional.” Previous research has shown that changes in urban consumption space are marked by place specificity and contextual variation, and that different cities do not evolve according to the same spatial logic [29,30]. At the same time, the relationship between coffee shops, teahouses, and other consumption spaces does not necessarily take the form of one-way substitution, but may instead involve coexistence, overlap, and restructuring [2,28]. The present study further shows that, even under the same data source, temporal window, and analytical framework, Jinan and Qingdao still followed different trajectories. This suggests that urban locality should be treated as an important context for interpreting POI-observed venue patterns, rather than merely as a background condition.

From a practical perspective, these findings also suggest that commercial development and urban governance should not treat coffee and tea as a simple zero-sum relationship. Existing studies show that the spatial provision and public-life significance of third places are themselves uneven [12,13], while traditional spaces such as teahouses can continue to support everyday sociality and the continuity of local culture in contemporary cities [28]. Mixed zones and persisting tea-related spaces, therefore, should not be seen merely as transitional states awaiting replacement, but may also constitute an important part of urban vitality. At the same time, the restructuring of consumption venues does not necessarily proceed through continuous one-way accumulation, but may instead be marked by temporality and fluctuation [31,32]. In this sense, coffee–tea venue restructuring in the two study cities is better understood as a process of ongoing adjustment, periodic fluctuation, and context-dependent reshaping.

### 4.5 Limitations

Several limitations should be noted. First, this study is based on platform-derived POI records and should therefore be interpreted as evidence of observed venue presence and spatial composition rather than as a complete representation of urban cultural practice itself. Although a unified data source, fixed annual observation point, and multiple rounds of data cleaning were used to improve comparability, the dataset may still be affected by platform update delays, record inconsistency, and shorter-term within-year or seasonal fluctuation. The results should therefore be understood as evidence of POI-observed cultural-space organization, not as a direct measure of lived cultural practice. Future research could strengthen this line of inquiry by combining POI data with review texts, social media traces, field observation, or interview-based evidence.

Second, the indicators used here capture related but non-equivalent dimensions. HI reflects compositional balance rather than market scale, interaction intensity, or cultural integration itself. CTI captures the magnitude of endpoint-based net change, whereas signed endpoint change indicates direction and trajectory types represent broader temporal pathways. These indicators are therefore complementary rather than interchangeable. The sensitivity analyses reduced the likelihood that the main findings were artifacts of a single mask definition, CR threshold, trajectory-classification rule, or endpoint choice, but they do not remove the general limitations of POI-based observational data.

Third, the present study remains comparative rather than causal. Trajectory types and functional zones help describe patterned temporal and spatial embedding, but they do not by themselves identify the independent effects of tourism, historical context, the COVID-19 period, or other background factors. In addition, because functional zones are empirically derived from POI composition and adjacent grids may exhibit spatial autocorrelation, the observed associations should be interpreted as contextual and descriptive rather than as strictly independent causal effects. Finally, although the unified framework improves comparability, the analysis is based on two cities only, and the findings should therefore be read as comparative evidence rather than as a complete typology of all Chinese cities.

## 5 Conclusion

This study examined changes in POI-observed coffee–tea venue patterns in Jinan and Qingdao from 2018 to 2024 using a unified 500 m × 500 m grid framework and platform-derived POI data. By combining city-level temporal comparison, grid-level cultural indicators, rule-based trajectory identification, functional-zone contextual analysis, and supplementary robustness checks, the study explored how coffee shops and teahouses coexisted, restructured, and shifted across urban space under a consistent comparative design.

The results do not support understanding coffee–tea venue change in the two cities as a simple one-way process in which coffee replaces tea. Instead, the two cities exhibited differentiated modes of restructuring. Jinan was characterized more by overall expansion, persistent coexistence, and bidirectional adjustment on the basis of a stronger tea-oriented structure, whereas Qingdao was characterized more by clearer coffee-oriented restructuring under relatively limited overall growth. At the same time, cultural mixing, directional transition, and temporal pathway types were unevenly distributed across urban space and were associated with different functional-zone contexts.

Taken together, these findings suggest that POI-observed coffee–tea venue restructuring is better understood as a differentiated process rather than as a single linear modernization pathway. More specifically, the observed patterns point to a process marked by coexistence, overlap, selective concentration, and uneven directional shifts. The comparison between Jinan and Qingdao further indicates that urban locality should be treated as an important context for interpreting how similar consumption venues are spatially reorganized in different cities.

At the same time, the findings should be interpreted within the boundaries of POI-observed venue presence and spatial composition rather than as a complete representation of lived cultural practice or a causal account of underlying drivers. Within these limits, the study provides comparative empirical evidence for understanding how emerging and established everyday consumption venues are reorganized, sustained, and unevenly embedded in different urban contexts.

## Supporting information

S1 Fig Spatial distribution and activity-volume-stratified patterns of the hybridization index (HI). (a) Spatial distribution of long-run HI in Jinan. (b) Spatial distribution of long-run HI in Qingdao. (c) HI distributions across period-aggregated coffee–tea activity-volume strata in Jinan and Qingdao. (d) Share of high-HI grids across period-aggregated coffee–tea activity-volume strata in Jinan and Qingdao. Note: HI was calculated from period-aggregated coffee-shop and teahouse counts over 2018–2024. Activity-volume strata were defined by the period-aggregated total number of coffee-shop and teahouse POIs in each grid. High-HI grids were defined as grids with HI ≥ 0.60.
(TIF)

S2 Fig Cultural-zone transition matrices between 2018 and 2024 in Jinan and Qingdao. (a) Cultural-zone transition matrix for Jinan. (b) Cultural-zone transition matrix for Qingdao. Note: Rows indicate the cultural-zone type in 2018, and columns indicate the cultural-zone type in 2024. Cell percentages are row percentages, and grid counts are shown in parentheses. Cultural zones were classified using the CR thresholds defined in the Methods.
(TIF)

S3 Fig Endpoint-sensitivity analysis of signed coffee–tea endpoint change. (a) Jinan: comparison between 2018 → 2024 and 2018 → 2022 signed endpoint changes. (b) Jinan: comparison between 2018 → 2024 and 2018 → 2023 signed endpoint changes. (c) Qingdao: comparison between 2018 → 2024 and 2018 → 2022 signed endpoint changes. (d) Qingdao: comparison between 2018 → 2024 and 2018 → 2023 signed endpoint changes. (e) Directional sign agreement under alternative CTI endpoints. Note: Signed endpoint change was calculated as the difference in CR between the base year and the endpoint year, preserving the direction of change. Positive values indicate coffee-ward change, and negative values indicate tea-ward change. Panel (e) reports sign agreement between the main endpoint comparison, 2018 → 2024, and the alternative endpoint comparisons, 2018 → 2022 and 2018 → 2023. “All grids” includes all paired valid grids, whereas “Nonzero CTI only” excludes grids with no endpoint change. The numerical summaries are reported in S9 Table.
(TIF)

S4 Fig Adjusted standardized residuals for functional zone × trajectory type in Jinan and Qingdao. (a) Adjusted standardized residuals for functional zone × trajectory type in Jinan. (b) Adjusted standardized residuals for functional zone × trajectory type in Qingdao. Note: Heatmap values are adjusted standardized residuals from chi-square tests. Positive residuals indicate combinations occurring more frequently than expected under independence, whereas negative residuals indicate combinations occurring less frequently than expected. * |residual| ≥ 1.96; ** |residual| ≥ 2.58.
(TIF)

S1 Appendix Data preprocessing and spatial framework. (DOCX)

S2 Appendix Cultural indicators and cultural zoning. (DOCX)

S3 Appendix Cultural trajectory identification. (DOCX)

S4 Appendix Functional zoning and contextual analyses. (DOCX)

S5 Appendix Sensitivity and supplementary robustness analyses. (DOCX)

S1 Table Stepwise construction of the study-area masks in Jinan and Qingdao. (XLSX)

S2 Table City-level annual totals, active grids, and year-on-year changes of coffee-shop and teahouse POIs in Jinan and Qingdao, 2018–2024. (XLSX)

S3 Table Grid counts and percentages by cultural transition direction in Jinan and Qingdao, 2018–2024. (XLSX)

S4 Table Grid counts and percentages by CTI class in Jinan and Qingdao. (XLSX)

S5 Table Cultural-zone transition matrix from 2018 to 2024 in Jinan and Qingdao. (XLSX)

S6 Table Composition of long-term cultural trajectory types in Jinan and Qingdao. (XLSX)

S7 Table Full cross-tabulations of functional zones with cultural zones and trajectory types in Jinan and Qingdao. (XLSX)

S8 Table Chi-square test summary for the associations between functional zones, cultural zones, and trajectory types in Jinan and Qingdao. (XLSX)

S9 Table Sensitivity summary for alternative CTI endpoint definitions in Jinan and Qingdao. (XLSX)

S10 Table Study-area mask sensitivity analysis. (XLSX)

S11 Table CR-threshold sensitivity analysis. (XLSX)

S12 Table Trajectory-classification sensitivity analysis. (XLSX)

S13 Table Summary of robustness findings. (XLSX)

S14 Table Spatial autocorrelation results. (XLSX)

S15 Table Functional-zone threshold sensitivity analysis for functional-zone associations with cultural zones and trajectory types. (XLSX)
