# Supplementary figures and images for: Comparative dynamics of coffee–tea cultural spaces in two Chinese cities: Evidence from Qingdao and Jinan, 2018–2024
Source: PLoS One. 2026 Aug 3;21(8):e0355398. doi: 10.1371/journal.pone.0355398 (PMC13432132; doi:10.1371/journal.pone.0355398)

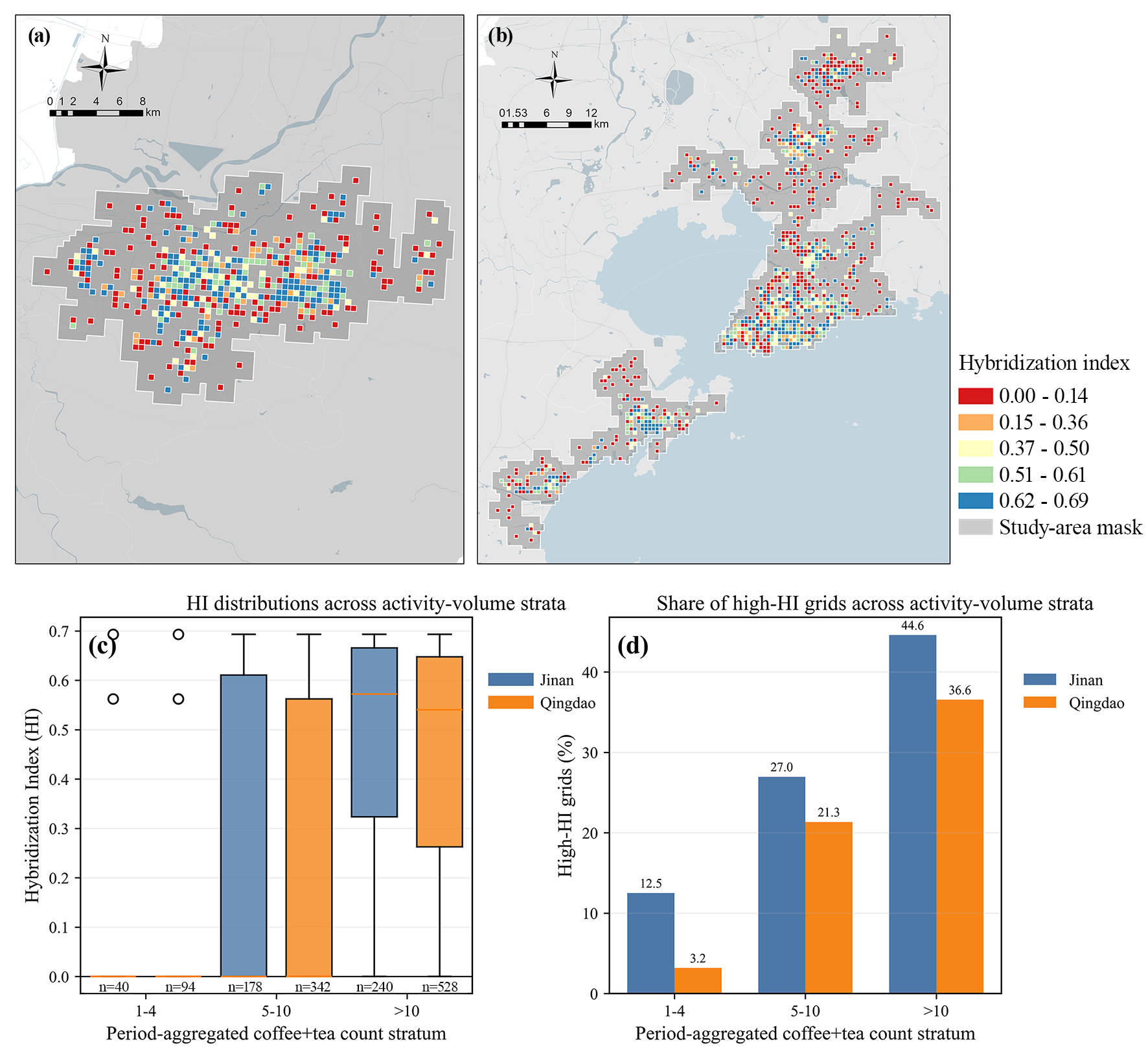

Supplement: S1 Fig — (a) Spatial distribution of long-run HI in Jinan. (b) Spatial distribution of long-run HI in Qingdao. (c) HI distributions across period-aggregated coffee–tea activity-volume strata in Jinan and Qingdao. (d) Share of high-HI grids across period-aggregated coffee–tea activity-volume strata in Jinan and Qingdao. Note: HI was calculated from period-aggregated coffee-shop and teahouse counts over 2018–2024. Activity-volume strata were defined by the period-aggregated total number of coffee-shop and teahouse POIs in each grid. High-HI grids were defined as grids with HI ≥ 0.60. (TIF) [file pone.0355398.s001.tif]

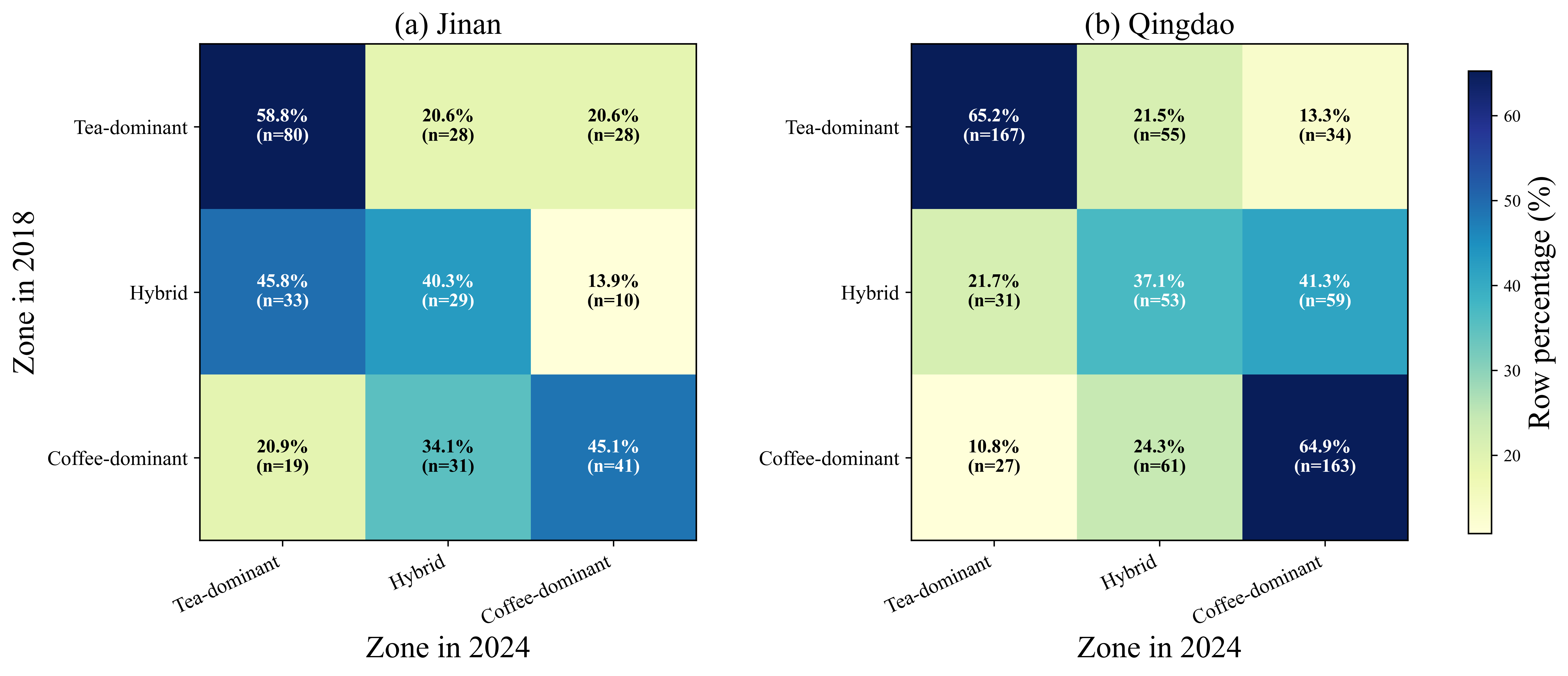

Supplement: S2 Fig — (a) Cultural-zone transition matrix for Jinan. (b) Cultural-zone transition matrix for Qingdao. Note: Rows indicate the cultural-zone type in 2018, and columns indicate the cultural-zone type in 2024. Cell percentages are row percentages, and grid counts are shown in parentheses. Cultural zones were classified using the CR thresholds defined in the Methods. (TIF) [file pone.0355398.s002.tif]

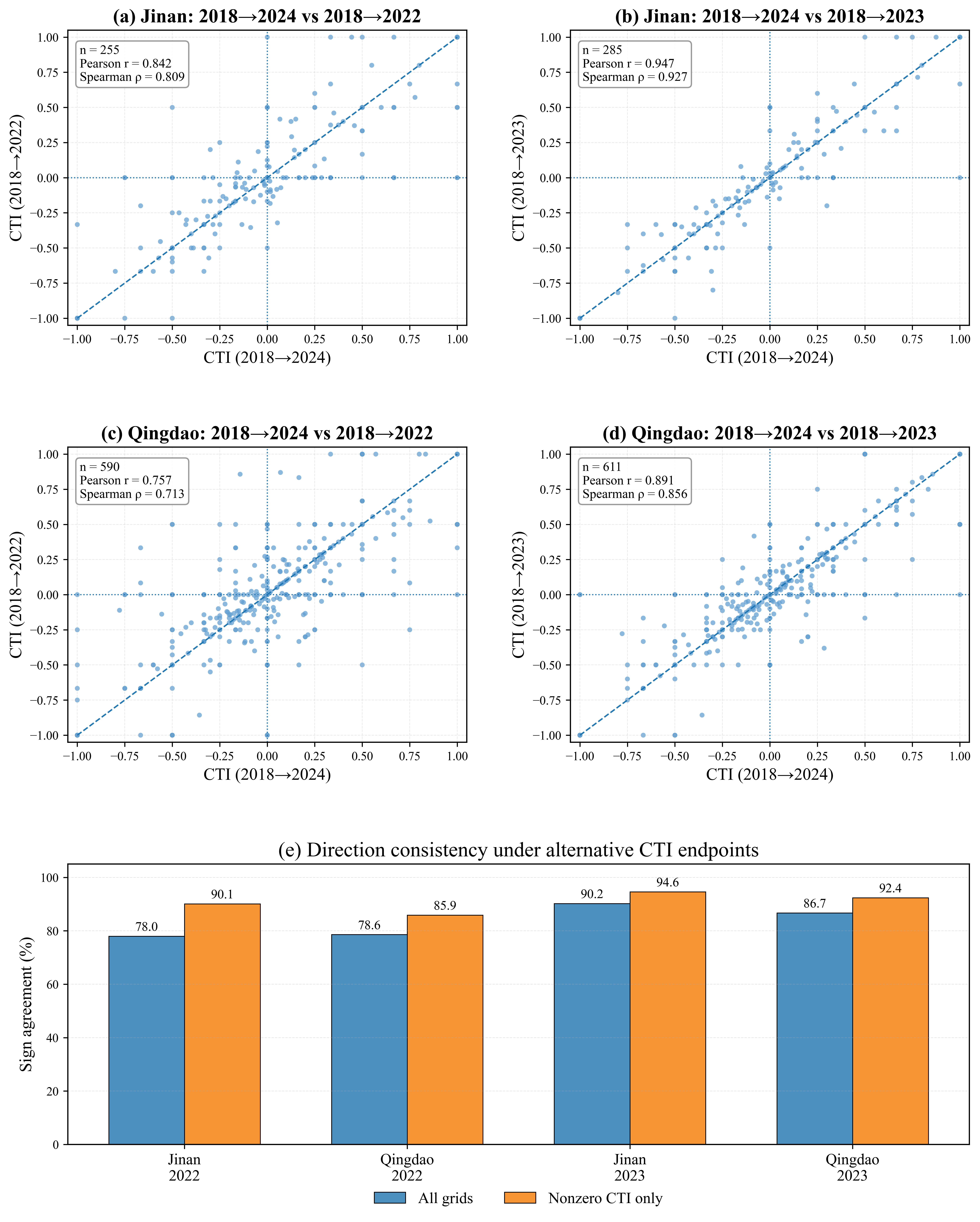

Supplement: S3 Fig — (a) Jinan: comparison between 2018 → 2024 and 2018 → 2022 signed endpoint changes. (b) Jinan: comparison between 2018 → 2024 and 2018 → 2023 signed endpoint changes. (c) Qingdao: comparison between 2018 → 2024 and 2018 → 2022 signed endpoint changes. (d) Qingdao: comparison between 2018 → 2024 and 2018 → 2023 signed endpoint changes. (e) Directional sign agreement under alternative CTI endpoints. Note: Signed endpoint change was calculated as the difference in CR between the base year and the endpoint year, preserving the direction of change. Positive values indicate coffee-ward change, and negative values indicate tea-ward change. Panel (e) reports sign agreement between the main endpoint comparison, 2018 → 2024, and the alternative endpoint comparisons, 2018 → 2022 and 2018 → 2023. “All grids” includes all paired valid grids, whereas “Nonzero CTI only” excludes grids with no endpoint change. The numerical summaries are reported in S9 Table. (TIF) [file pone.0355398.s003.tif]

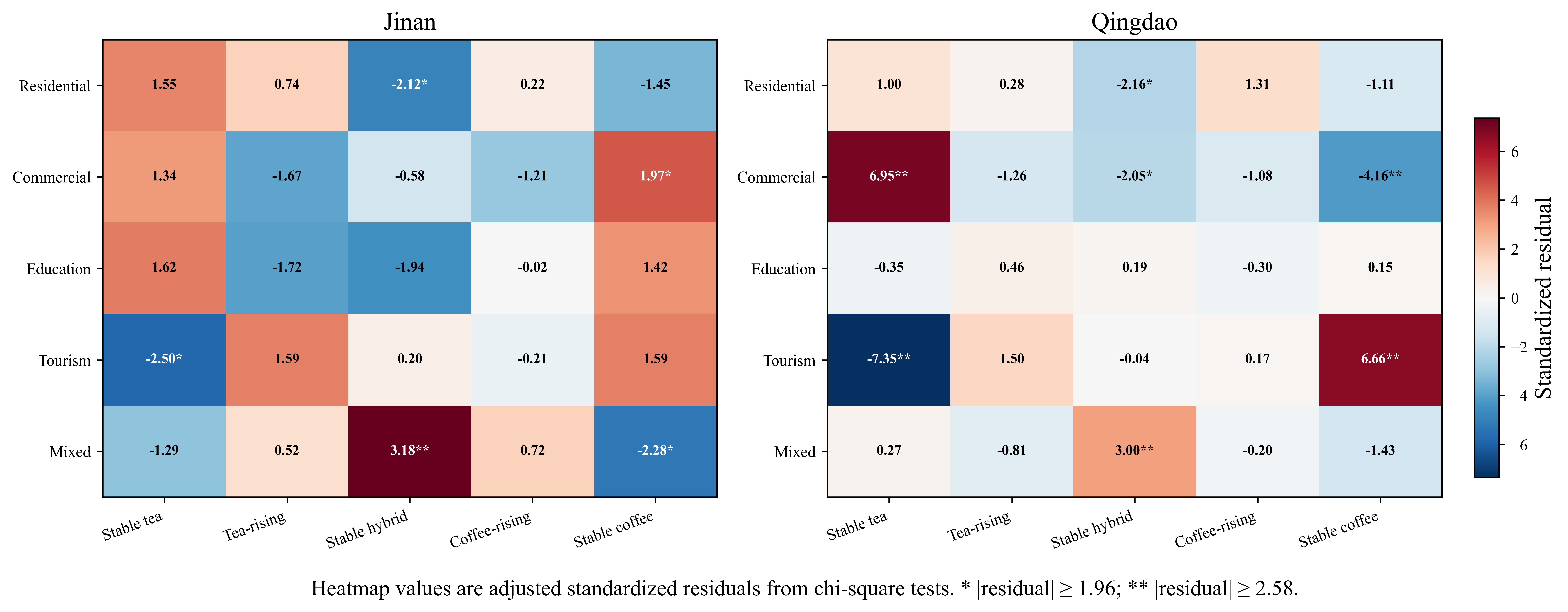

Supplement: S4 Fig — (a) Adjusted standardized residuals for functional zone × trajectory type in Jinan. (b) Adjusted standardized residuals for functional zone × trajectory type in Qingdao. Note: Heatmap values are adjusted standardized residuals from chi-square tests. Positive residuals indicate combinations occurring more frequently than expected under independence, whereas negative residuals indicate combinations occurring less frequently than expected. * |residual| ≥ 1.96; ** |residual| ≥ 2.58. (TIF) [file pone.0355398.s004.tif]
