# Supplementary material for: Comparative dynamics of coffee–tea cultural spaces in two Chinese cities: Evidence from Qingdao and Jinan, 2018–2024
Source: PLoS One. 2026 Aug 3;21(8):e0355398. doi: 10.1371/journal.pone.0355398 (PMC13432132; doi:10.1371/journal.pone.0355398)
Supplement: S1 Appendix — (DOCX) [file pone.0355398.s005.docx]

**S1 Appendix. Data preprocessing and spatial framework**

This appendix documents the upstream data-processing workflow and spatial framework used in the comparative analysis of POI-observed coffee–tea cultural-space patterns in Jinan and Qingdao. The study-wide indicators and analytical concepts are summarized in Table 1 in the main text. The present appendix focuses specifically on data acquisition, record cleaning, category harmonization, coordinate standardization, fixed-grid construction, POI-to-grid assignment, and the stepwise delineation of the main study-area masks. Stepwise retention counts for mask construction are reported in Table S1.

# **S1.1 POI data sources**

This study used two POI sources for different analytical purposes. Dianping POIs were used to identify coffee-shop and teahouse venues, whereas Amap (Gaode Map) POIs were used to construct grid-level functional-zone context. The two sources were not treated as equivalent POI systems for direct comparison; rather, they served separate analytical roles within the same 500 m × 500 m grid framework.

Dianping POIs were used for coffee-shop and teahouse identification because Dianping is a consumption-oriented local-service platform with detailed venue classifications for food, beverage, and leisure consumption. In this study, Dianping records provided the primary basis for measuring the observed presence of coffee-shop and teahouse venues from 2018 to 2024. Each Dianping record contained a stable platform venue identifier, establishment name, address, platform category label, geographic coordinates, and observation year.

Amap POIs were used to construct functional-zone context because Amap provides broader coverage of urban facilities and services. These records were used to describe the functional context of each grid cell and were not used to define the main coffee–tea venue indicators.

Because platform category taxonomies and labeling rules may change over time, raw platform categories were not assumed to be temporally invariant. Year-specific category-harmonization procedures were therefore implemented separately for Dianping coffee–tea venue classification and Amap functional-zone recoding.

Annual POI snapshots were organized by observation year to maintain a consistent temporal framework for cross-year comparison during 2018–2024.

# **S1.2 Dianping coffee-shop and teahouse POI data cleaning and classification**

## **S1.2.1 ID-based deduplication and proximity screening**

Dianping POI records were deduplicated using the stable platform venue identifier as the primary key rather than relying on establishment names alone. To identify likely duplicate records and positional jitter within the same year, all records were first screened in EPSG:32650 using a distance-based rule.

Records located within 50 m of one another and sharing the same Dianping venue ID were treated as likely positional jitter or near-duplicate entries and were flagged for consolidation review. Records located within 50–100 m were not automatically treated as duplicates but were flagged for targeted manual checking. This two-level screen reduced false merging while preserving sensitivity to platform-based coordinate drift.

## **S1.2.2 Targeted manual verification for inconsistent ID–address pairs**

Targeted manual checks were conducted for cases in which the same Dianping venue ID appeared with multiple addresses within the same year. These cases were reviewed to determine whether they represented the same establishment with relocation or renaming, or distinct establishments incorrectly sharing an identifier.

Records with unresolved ID–address inconsistency were excluded from ID-based longitudinal linkage in order to reduce downstream measurement uncertainty.

## **S1.2.3 Year-specific category harmonization for coffee-shop and teahouse venues**

Dianping’s platform categories were used as the initial operational basis for identifying coffee-shop and teahouse venues. Because platform categories may change over time and some beverage-related venues have ambiguous business identities, raw Dianping category labels were harmonized into two stable analytical categories: Coffee shop and Teahouse.

Coffee shops were defined as venues whose primary business identity was coffee consumption, including coffee shops, cafés, coffee houses, and specialty coffee stores.

Teahouses were defined as venues whose primary business identity was on-site tea drinking, tea-room consumption, or traditional tea-related social consumption. This definition includes tea houses, tea rooms, traditional teahouses, tea clubs, tea ceremony spaces, and comparable Chinese categories such as 茶馆, 茶楼, 茶室, and 茶社 when the venue primarily functioned as an on-site tea consumption space.

Milk-tea shops, fruit-tea shops, general beverage shops, dessert drink shops, restaurants with incidental tea service, non-coffee-oriented cafés, and ambiguous mixed beverage venues were excluded unless their primary business identity clearly matched the operational definition of coffee shop or teahouse.

For each year, raw Dianping category labels were mapped to the target analytical classes using a consistent rule-based procedure. The procedure followed a fixed priority order.

**Rule 1:** Direct category mapping. If a raw category label clearly corresponded to coffee shop or teahouse in that year, it was mapped directly.

**Rule 2:** Cross-year reconciliation. If a category was renamed, split, or merged in later years, the revised label was mapped back to the same analytical class as its nearest conceptual equivalent in earlier years.

**Rule 3:** Keyword-assisted validation. For coffee and tea identification, category mapping was cross-checked using name or label keywords to reduce misclassification when platform categories were ambiguous.

**Rule 4:** Manual review of ambiguous cases. Ambiguous cases were reviewed based on venue name, platform category label, and available descriptive information. Records that could not be confidently assigned to either coffee shop or teahouse were excluded from the analytical coffee–tea dataset.

The category-harmonization rules are documented in this appendix. Annual city-level summaries of coffee-shop and teahouse POI counts are provided in Table S2.

This harmonization ensured that coffee-shop and teahouse indicators were derived from a conceptually stable classification system while explicitly acknowledging annual taxonomy adjustments at the source-data level. Although keyword-assisted validation and manual review were used to reduce misclassification, some residual classification error may remain, especially for venues with ambiguous labels, multifunctional menus, or changing business identities.

## **S1.2.4 Coordinate reference system**

All POI records and spatial layers were transformed into a unified projected coordinate system, WGS84 / UTM Zone 50N (EPSG:32650). All spatial operations, including distance screening, spatial joins, clustering, and area-based thresholding, were conducted in this CRS.

# **S1.3 Amap functional POI preprocessing and recoding**

Amap POIs were used to construct grid-level functional-zone context. Unlike Dianping coffee–tea POIs, which were used to identify the main study objects, Amap POIs were used to describe the broader urban functional environment of each grid cell.

Amap’s raw platform categories were harmonized into a stable set of urban functional categories, including Commercial, Residential, Education, Tourism, and other relevant functional groups used for grid-level functional-zone classification. Because Amap’s taxonomy may change across years through category splitting, merging, renaming, or code reassignment, a year-specific recoding strategy was implemented to ensure temporal comparability.

To reduce mechanical overlap with the Dianping-based coffee–tea venue indicators, coffee-shop, teahouse, milk-tea, fruit-tea, general beverage, and dessert drink categories were excluded from the Amap-based functional-zone classification. This exclusion was applied before calculating functional-category counts and proportions. The resulting functional-zone variables were used as contextual descriptors rather than causal explanatory variables.

# **S1.4 Construction of the study-area mask**

To focus the analysis on effective urban consumption space while reducing noise from sparse peripheral observations, city-specific study-area masks were delineated for Jinan and Qingdao using the same stepwise grid-based filtering and spatial refinement procedure. Stepwise retention counts are reported in Table S1.

**Step 1. Activity presence**

Dianping coffee-shop and teahouse POIs observed during 2018–2024 were assigned to the fixed grid using the deterministic assignment rule described in Section S1.6. Grid cells were initially retained if at least one Dianping-listed coffee-shop or teahouse POI was observed in any year during the study period.

**Step 2. Minimum cumulative activity intensity**

To remove sporadic observations, retained grid cells were further required to have a cumulative coffee-shop + teahouse count of at least 4 across 2018–2024.

**Step 3. Temporal persistence of observed activity**

To ensure that retained cells reflected repeated rather than incidental activity, grid cells meeting Step 2 were further required to show coffee–tea activity in at least 4 distinct years during 2018–2024. Cells satisfying both Step 2 and Step 3 were treated as core activity grids.

**Step 4. Queen-adjacency-based spatial expansion**

To preserve neighborhood-scale spatial continuity, the core activity grids were expanded through two rounds of queen adjacency, allowing both edge-touching and corner-touching neighboring cells to be included at this expansion stage.

**Step 5. Cluster-level refinement**

Expanded cells were grouped into contiguous clusters using a stricter connectivity rule that excluded corner-only contacts. In other words, two cells were treated as connected only when they shared a boundary segment of non-zero length. To improve cross-city comparability and reduce residual fragmentation, only clusters with a total area of at least 120 km² were retained.

**Step 6. Filling enclosed interior holes**

To reduce artificial fragmentation caused by internal gaps, retained clusters were dissolved into polygons and enclosed interior holes were extracted. Holes with an area of 10 km² or less were filled by adding all grid cells whose centroids fell within the hole polygons.

The resulting grid set in each city constituted the main study-area mask used in all subsequent grid-based analyses. These masks represent stable and spatially interpretable urban consumption areas defined by the long-term observed presence of Dianping-listed coffee-shop and teahouse venues, rather than administrative built-up areas or complete municipal extents.

Because the main study-area masks were derived from observed coffee–tea venue activity, supplementary sensitivity checks were conducted using alternative mask definitions, including an expanded active mask and a one-ring expanded mask based on Queen contiguity. These checks were used to assess whether the main city-level contrasts and spatial patterns were sensitive to the mask construction procedure. The sensitivity results are reported in the Supporting Information.

# **S1.5 Grid generation**

A regular 500 m × 500 m grid was generated in EPSG:32650 as the fixed spatial framework for both cities. The same grid definition was used consistently across all study years for spatial joins, annual aggregation, and all subsequent grid-based analyses. Each grid cell was assigned a unique grid identifier.

# **S1.6 POI-to-grid assignment and grid–year dataset construction**

POIs were assigned to grid cells using a deterministic two-step spatial rule under EPSG:32650.

First, a point-in-polygon spatial join with the predicate *within* was applied.

Second, for unmatched points located on or near grid boundaries, a constrained nearest-neighbor assignment was applied, assigning the point to the nearest grid cell within 50 m. If the nearest distance exceeded 50 m, the record remained unassigned.

This rule ensured deterministic treatment of boundary-adjacent points while limiting unreliable matches.

Using the resulting grid assignments, Dianping coffee-shop and teahouse POIs were aggregated to calculate annual coffee-shop counts, teahouse counts, and total coffee–tea venue counts for each grid–year combination from 2018 to 2024, producing a standardized grid-by-year panel dataset. These counts formed the basis for calculating the main coffee–tea indicators, including CR, HI, CTI, cultural-zone classification, and trajectory types.

Separately, Amap functional POIs were assigned to the same grid framework and summarized by harmonized functional categories within each grid to generate category-wise counts and proportions. These Amap-derived functional summaries were used only for functional-zone context and were not used to define coffee-shop or teahouse venue counts.

# **S1.7 Reproducibility notes and interpretation boundaries**

The spatial framework was established through two POI sources: Dianping records for coffee-shop and teahouse venue identification and Amap records for functional-zone context. The workflow included annual POI snapshots, year-specific category harmonization, ID-based cleaning with proximity screening and targeted manual verification, consistent projection to EPSG:32650, a fixed 500 m × 500 m grid, and an explicitly parameterized study-area-mask construction procedure.

All interpretations are restricted to observed spatial patterns represented by POI records. Because POI data may be affected by platform update delays, identifier changes, relocation, renaming, category revision, and duplicate or ambiguous records, results should be interpreted conservatively as patterns of observed presence and spatial change rather than as administrative records of opening, closure, or legal land-use designation.

More fundamentally, the POI-based framework used in this study captures the observed presence and spatial composition of platform-listed venues rather than cultural meaning, interaction intensity, or consumption practice itself. A higher coffee or tea share in a grid therefore indicates a stronger observed venue presence of that category in the POI record, not a direct measure of how strongly that grid functions as a coffee- or tea-centered cultural setting in lived social terms. In addition, platform visibility may vary across venue types, business sizes, and operating styles, which means that some traditional or less digitized establishments may be underrepresented relative to more standardized or platform-visible venues.

Because the coffee–tea venue indicators and functional-zone context were derived from different POI platforms, platform-specific coverage and classification differences may remain. Functional-zone results were therefore interpreted as contextual associations rather than causal effects.

The fixed 500 m × 500 m grid provided a common and reproducible spatial framework for both cities and all study years, but it also shapes the degree to which fine-grained local heterogeneity can be detected. The present results should therefore be interpreted as evidence of POI-observed cultural-space organization within a fixed comparative grid framework rather than as a complete representation of urban cultural practice.
