# Supplementary material for: Comparative dynamics of coffee–tea cultural spaces in two Chinese cities: Evidence from Qingdao and Jinan, 2018–2024
Source: PLoS One. 2026 Aug 3;21(8):e0355398. doi: 10.1371/journal.pone.0355398 (PMC13432132; doi:10.1371/journal.pone.0355398)
Supplement: S2 Appendix — (DOCX) [file pone.0355398.s006.docx]

**S2 Appendix. Cultural indicators and cultural zoning**

This appendix defines the POI-based cultural indicators used in the study and specifies the operational rules for grid-level cultural zoning. All indicators were computed within the fixed 500 m × 500 m grid framework described in S1 Appendix. The purpose of this appendix is to make explicit how coffee–tea composition was translated into three complementary cultural indicators—Coffee Ratio (CR), Hybridization Index (HI), and Cultural Transition Intensity (CTI)—and how these indicators were used to define spatial cultural zones and transition hotspots. Because spatial cultural zones and temporal trajectory types were constructed from different temporal aggregation schemes, they were treated as analytically related but methodologically distinct.

# **S2.1 Coffee Ratio (CR)**

Coffee Ratio (CR) was used to represent the relative dominance of coffee-related establishments within each grid cell. For grid g and year t, annual CR was defined as:

CR(g,t) = C(g,t) / [C(g,t) + T(g,t)]

where C(g,t) and T(g,t) denote the observed numbers of Dianping-listed coffee-shop and teahouse venues, respectively, in grid g during year t.

CR ranges from 0 to 1, where:

CR = 0 indicates complete tea dominance;
CR = 1 indicates complete coffee dominance.

CR was selected because it provides a simple and comparable measure of relative coffee–tea venue composition while remaining interpretable across grids with different activity volumes.

# **S2.2 Temporal aggregation strategies of CR**

CR was computed using two temporal aggregation strategies for different analytical purposes.

First, annual CR was calculated separately for each year from 2018 to 2024. Annual CR was used for time-sensitive analyses, including endpoint-based transition measurement and trajectory identification based on observed annual sequences.

Second, period-aggregated CR was calculated by pooling coffee and tea counts across the full study period:

CR(g, 2018–2024) = [Σ C(g,t)] / [Σ C(g,t) + Σ T(g,t)], for t = 2018, …, 2024

This period-aggregated CR was used to identify spatial cultural zones. The annual and period-aggregated CR measures were derived from the same underlying grid–year panel dataset but served different analytical purposes. This separation was adopted to avoid circular definitions and to preserve the distinction between period-aggregated spatial orientation and interannual temporal change.

# **S2.3 Hybridization Index (HI)**

To quantify the balance of coffee–tea coexistence within each grid, we used a Shannon entropy–based Hybridization Index (HI). Let p(g) denote the period-aggregated coffee share in grid g, such that:

p(g) = [Σ C(g,t)] / [Σ C(g,t) + Σ T(g,t)], for t = 2018, …, 2024

and the corresponding tea share is:

1 − p(g)

HI was defined as:

HI(g) = −[p(g) × ln p(g) + (1 − p(g)) × ln(1 − p(g))]

HI was not normalized. Accordingly, HI ranged from 0 to ln(2) (approximately 0.693). Higher HI values indicate more balanced co-presence between coffee-shop and teahouse venue categories, whereas lower HI values indicate stronger dominance by one category.

For numerical stability, terms of the form 0 × ln(0) were defined as 0 using the standard limit. Thus, when p(g) = 0 or p(g) = 1, the corresponding entropy term was set to 0.

Because HI was derived from period-aggregated composition, it was interpreted as a long-run measure of balance in coexistence rather than a year-specific fluctuation metric. Importantly, HI captures compositional balance rather than absolute activity volume. It should therefore be interpreted as a measure of balance in coexistence, not as a direct indicator of the scale of commercial activity.

# **S2.4 Descriptive support for HI across activity-volume strata**

Because HI does not incorporate absolute activity volume, we additionally summarized HI distributions across strata of period-aggregated coffee + tea counts as a descriptive support analysis. This comparison was intended only as an interpretive aid and did not alter the formal definition of HI. For each grid, the period-aggregated total count was defined as:

N(g) = Σ C(g,t) + Σ T(g,t), for t = 2018, …, 2024

The comparison was restricted to grids included in the long-run HI analysis. Period-aggregated activity volume was grouped into three strata (1-4, 5–10, and >10 total coffee-shop + teahouse observations across 2018–2024). Within each stratum and city, we summarized the median HI, the interquartile range, the share of grids with HI = 0, and the share of highly mixed grids defined as HI ≥ 0.60.

The stratified comparison showed that very high HI values were not concentrated primarily in the lowest-activity grids. In the 1–4 stratum, the median HI was 0 in both cities, while grids with HI = 0 accounted for 80.0% of the stratum in Jinan and 91.5% in Qingdao. As activity volume increased, the share of highly mixed grids (HI ≥ 0.60) also increased, rising from 12.5%, 27.0%, and 44.6% across the three strata in Jinan and from 3.2%, 21.3%, and 36.6% in Qingdao. In the highest-activity stratum (>10), Jinan still showed a slightly higher median HI than Qingdao (0.572 vs. 0.540), together with a larger share of highly mixed grids and a smaller share of HI = 0 grids (14.2% vs. 19.1%). These results suggest that the stronger long-run mixing observed in Jinan cannot be reduced solely to sparse low-count balanced grids, although the present comparison remains descriptive rather than inferential (Fig. S1c,d).

# **S2.5 Cultural Transition Intensity (CTI) as endpoint-based net directional change**

To capture directional net cultural transition over time, we defined Cultural Transition Intensity (CTI) as the endpoint-based change in annual CR between 2018 and 2024:

CTI (g, 2018→2024) = CR (g, 2024) – CR (g, 2018)

CTI is directional. Positive values indicate a shift toward coffee dominance (coffee-ward transition), whereas negative values indicate a shift toward tea dominance (tea-ward transition). The absolute value |CTI| represents the magnitude of net transition between the two endpoints.

CTI was computed only for grids with valid annual CR values in both 2018 and 2024. In other words, both endpoint years were required to have non-zero combined coffee + tea observations so that CR could be validly computed.

CTI was interpreted as complementary to, rather than interchangeable with, trajectory types. CTI summarizes net endpoint displacement between two fixed years, whereas trajectory types summarize broader temporal pathways using all observed annual CR values.

Because CTI is endpoint-based, it was supplemented by annual transition and trajectory analyses reported in S3 Appendix and related Supporting Information tables. Endpoint transition directions and directional CTI classes are summarized in Tables S3 and S4.

# **S2.6 Identification of cultural zones based on period-aggregated CR**

Grid-level cultural zones were classified using threshold rules applied to period-aggregated CR:

Tea-dominant zone: CR (g, 2018–2024) ≤ 0.30

Hybrid zone: 0.30 < CR (g, 2018–2024) < 0.70

Coffee-dominant zone: CR (g, 2018–2024) ≥ 0.70

These thresholds define a hybrid band centered on balanced coexistence and two dominance regimes at the extremes. Because these cultural zones were derived from period-aggregated CR rather than annual sequences, they were interpreted as descriptors of period-aggregated spatial orientation rather than any single-year state.

In later analyses, cultural zones served as contextual spatial labels and were interpreted jointly with, but analytically separately from, temporal trajectory types. Endpoint cultural-zone transitions are summarized in Table S5.

# **S2.7 Identification of strong endpoint-transition grids for sensitivity diagnostics**

For endpoint-window sensitivity diagnostics, strong endpoint-transition grids were identified using an absolute CTI threshold. A grid was labeled as a strong endpoint-transition grid when |CTI| ≥ 0.80. This rule identifies grids with large endpoint displacement in coffee–tea composition, regardless of whether the direction was coffee-ward or tea-ward. Direction was retained separately using the sign of CTI: positive CTI indicates coffee-ward change, whereas negative CTI indicates tea-ward change.

This diagnostic definition was used for endpoint-window overlap checks and was not intended to replace the main CTI definition or to imply a spatial-cluster statistic. It provides a simple reproducible rule for comparing whether grids with large endpoint changes remain similar under alternative endpoint-year specifications.

# **S2.8 Interpretation boundaries**

The three cultural indicators used in this study capture related but distinct analytical dimensions:

CR captures relative coffee–tea venue dominance; HI captures balance in co-presence based on period-aggregated composition; CTI captures endpoint-based directional net transition.

These indicators were used jointly to characterize period-aggregated spatial orientation and temporal change, but they should not be interpreted as interchangeable measures. In particular, HI should not be interpreted as a measure of absolute commercial activity volume, and CTI should not be interpreted as a full-sequence temporal descriptor. Temporal trajectory types, defined separately in S3 Appendix, remain necessary for summarizing longer-term pathway structure beyond endpoint change.

More broadly, all three indicators should be interpreted as descriptors of observed coffee–tea composition derived from POI records rather than as direct measures of cultural meaning, social interaction intensity, or symbolic identity. Because the indicators are based on platform-recorded venue presence, they remain sensitive to classification error, category revision, platform update delays, and unequal visibility of different venue types. The resulting cultural zones and transition measures should therefore be read as representations of POI-observed spatial organization and temporal change within the fixed grid framework, rather than as complete representations of lived cultural practice.
