# Supplementary material for: Comparative dynamics of coffee–tea cultural spaces in two Chinese cities: Evidence from Qingdao and Jinan, 2018–2024
Source: PLoS One. 2026 Aug 3;21(8):e0355398. doi: 10.1371/journal.pone.0355398 (PMC13432132; doi:10.1371/journal.pone.0355398)
Supplement: S3 Appendix — (DOCX) [file pone.0355398.s007.docx]

**S3 Appendix. Cultural trajectory identification**

This appendix specifies how grid-level cultural trajectory types were identified to summarize temporal pathways in POI-observed coffee–tea venue composition. Unlike spatial cultural zones, which were derived from period-aggregated Coffee Ratio (CR) and describe period-aggregated spatial orientation, trajectory types were constructed from annual CR sequences and were used to characterize temporal stability and directional change within each grid across the study period. The purpose of this appendix is to provide a reproducible rule-based framework for assigning each eligible grid to one of five trajectory types: stable tea, stable hybrid, stable coffee, tea rising, or coffee rising.

# **S3.1 Overview and analytical purpose**

Trajectory types were designed to summarize how annual coffee–tea composition evolved over time within each grid cell from 2018 to 2024. They were intended to capture whether a grid remained persistently tea-oriented, persistently hybrid, persistently coffee-oriented, or instead showed an overall directional shift toward tea or coffee over the study period. Trajectory types were treated as temporal descriptors rather than as direct records of business history or administrative land-use transformation.

Trajectory types were not treated as interchangeable with either spatial cultural zones or Cultural Transition Intensity (CTI). Spatial cultural zones were derived from period-aggregated CR and represent period-aggregated spatial orientation, whereas CTI captures endpoint-based net directional change between 2018 and 2024. By contrast, trajectory types summarize the broader temporal pathway represented by the observed annual CR sequence. This separation was maintained in order to preserve the distinction between long-run spatial structure, endpoint-based net change, and full-sequence temporal pathway structure.

# **S3.2 Eligibility criteria for trajectory assignment**

Trajectory assignment was restricted to grids with sufficient temporal information. A grid cell was considered eligible only if it had valid coffee + tea observations in at least four years during 2018–2024 and a cumulative coffee + tea count of at least four across the study period. Only eligible grids were used for trajectory classification. These criteria were adopted to reduce instability caused by sparse observations and to ensure that assigned trajectory labels reflected sustained observed temporal patterns rather than isolated annual records.

No temporal interpolation was applied to missing grid–year observations. Years without valid coffee or tea observations were retained as non-observed rather than imputed, and classification was based only on observed annual values. This rule was especially important for temporal analyses because trajectory labels were intended to reflect observed dynamics rather than interpolated change.

# **S3.3 Annual CR slope and mean annual CR**

For each eligible grid, trajectory classification was based on the observed annual CR sequence during 2018–2024. No temporal interpolation was applied to missing grid–year observations. Years without valid coffee–tea observations were treated as non-observed and were not imputed.

Two quantities were calculated for each eligible grid. First, a simple linear slope of annual CR against year was calculated using the observed valid annual CR values. This slope summarizes the overall direction of change in annual coffee–tea composition across the observed sequence. Second, the mean annual CR was calculated across all valid observed years. The mean annual CR was used to distinguish stable tea-oriented, stable hybrid, and stable coffee-oriented trajectories when the slope did not indicate a clear directional trend.

The main trajectory rule used a stable-band threshold of 0.05 for the annual CR slope. This threshold was used to distinguish directional trajectories from broadly stable trajectories. The CR thresholds of 0.30 and 0.70 were then used to classify the mean annual CR of non-directional trajectories.

# **S3.4 Five-category slope-based trajectory assignment**

Trajectory labels were assigned using a deterministic rule set. For each eligible grid, the annual CR slope was evaluated first. If the slope was greater than 0.05, the grid was classified as coffee rising. If the slope was less than −0.05, the grid was classified as tea rising.

If the slope fell within the stable band from −0.05 to 0.05, the grid was classified using its mean annual CR. Grids with mean annual CR at or below 0.30 were classified as stable tea. Grids with mean annual CR at or above 0.70 were classified as stable coffee. The remaining grids were classified as stable hybrid.

This procedure ensured that directional trajectories were identified from the overall annual CR trend, whereas stable trajectory categories were assigned only after excluding grids with a clear positive or negative annual CR slope.

# **S3.5 Five-category rule-based trajectory assignment**

## **S3.5.1 Stable tea**

A grid was classified as stable tea when its annual CR slope fell within the stable band and its mean annual CR was at or below 0.30. In substantive terms, stable tea represents grids whose observed annual coffee–tea composition remained broadly tea-oriented across the study period without a clear directional trend toward coffee or tea.

## **S3.5.2 Stable hybrid**

A grid was classified as stable hybrid when its annual CR slope fell within the stable band and its mean annual CR was greater than 0.30 and less than 0.70. Stable hybrid therefore represents grids whose observed annual coffee–tea composition remained broadly mixed across the study period without a clear directional trend toward either coffee dominance or tea dominance. It differs from the hybrid cultural zone in S2 Appendix: the hybrid cultural zone is a period-aggregated spatial classification, whereas stable hybrid is a temporal pathway label derived from the observed annual CR sequence.

## **S3.5.3 Stable coffee**

A grid was classified as stable coffee when its annual CR slope fell within the stable band and its mean annual CR was at or above 0.70. Stable coffee therefore captures grids whose observed annual coffee–tea composition remained broadly coffee-oriented across the study period without a clear directional trend toward coffee or tea.

## **S3.5.4 Tea rising**

A grid was classified as tea rising when its annual CR slope was less than −0.05. This category identifies grids whose observed annual CR sequence showed a clear negative trend over the study period, indicating an overall shift toward a higher relative presence of teahouses. Tea-rising trajectories were defined by the slope of the observed annual CR sequence rather than by the endpoint difference alone.

## **S3.5.5 Coffee rising**

A grid was classified as coffee rising when its annual CR slope was greater than 0.05. This category identifies grids whose observed annual CR sequence showed a clear positive trend over the study period, indicating an overall shift toward a higher relative presence of coffee shops. Coffee-rising trajectories were defined by the slope of the observed annual CR sequence rather than by the endpoint difference alone.

# **S3.6 Decision order and reproducibility**

To ensure deterministic and reproducible assignment, trajectory labels were assigned in a fixed order. The annual CR slope was evaluated first to identify coffee-rising and tea-rising trajectories. Only grids whose slope fell within the stable band were then classified into stable tea, stable hybrid, or stable coffee according to mean annual CR. Because the classification rule was based on continuous slope and mean CR values, no separate endpoint-direction tie-handling rule was used. Given the same annual grid-level CR sequence, the same stable-band threshold, and the same CR thresholds, the resulting trajectory label is deterministic.

# **S3.7 Interpretation boundaries**

Trajectory types should be interpreted as rule-based summary labels of observed temporal pathways rather than as direct evidence of pandemic causality or administrative land-use transformation. They do not identify opening and closure histories, nor do they provide a complete account of all short-term variation occurring between observed years. Instead, they summarize broad temporal pathway structure within the limits of the available annual POI observations.

Trajectory types are complementary to, rather than interchangeable with, either cultural zones or CTI. Cultural zones describe period-aggregated spatial orientation, CTI summarizes endpoint-based net displacement between 2018 and 2024, and trajectory types summarize broader temporal pathways using all observed annual CR values. Accordingly, trajectory types were used to support comparative interpretation of broad temporal patterns across Jinan and Qingdao rather than to replace either endpoint-based transition measures or period-aggregated spatial labels.

Because trajectory assignment was based only on observed annual values, the resulting labels remain sensitive to sparse observations, missing years, and residual classification uncertainty in the underlying POI records. They should therefore be interpreted as reproducible summaries of observed sequence structure within the fixed grid framework, not as complete representations of all temporal variation in everyday cultural practice.

Short-term reversals were examined separately as a supplementary check and were not used to replace the main five-category trajectory classification. The resulting city-level composition of the five trajectory types is reported in Table S6.
