# Supplementary material for: Comparative dynamics of coffee–tea cultural spaces in two Chinese cities: Evidence from Qingdao and Jinan, 2018–2024
Source: PLoS One. 2026 Aug 3;21(8):e0355398. doi: 10.1371/journal.pone.0355398 (PMC13432132; doi:10.1371/journal.pone.0355398)
Supplement: S4 Appendix — (DOCX) [file pone.0355398.s008.docx]

**S4 Appendix. Functional zoning and contextual analyses**

This appendix describes the grid-level functional-zoning procedure used to characterize the broader urban context associated with the distribution of POI-observed coffee–tea venue patterns. Consistent with the main text, functional zoning was constructed as an empirical urban-context descriptor and was treated as analytically separate from the cultural indicators and trajectory types. It was used only to contextualize subsequent comparisons of cultural zones, trajectory types, and related spatial patterns.

# **S4.1 Purpose and scope**

The purpose of functional zoning in this study was to represent the prevailing urban activity context of each 500 m × 500 m grid cell within the main study-area mask. Functional zoning was not intended to reproduce formal land-use designation or statutory zoning. Instead, it was used to derive an empirical contextual layer from the composition of harmonized functional POI categories aggregated within each grid cell.

This appendix should be read together with S1 Appendix, which documents category harmonization, grid cell construction, and study-area mask delineation; S2 Appendix, which defines the cultural indicators and cultural zones; and S3 Appendix, which defines the five-category trajectory framework. Functional zones were used only to interpret how long-run cultural zones and temporal trajectory types were situated across residential, commercial, educational, tourism-oriented, and mixed-use urban settings.

# **S4.2 Grid-level functional-zoning procedure**

Using the harmonized urban functional categories described in S1 Appendix, all relevant functional POIs within the main study-area mask during 2018–2024 were aggregated to the fixed 500 m × 500 m grid. For each grid cell, category-wise POI counts were summarized and converted into within-grid proportions across the retained functional categories: Commercial, Residential, Education, and Tourism.

As described in S1 Appendix, the functional-zone layer was constructed from Amap POIs rather than from the Dianping coffee–tea venue dataset. Coffee-shop, teahouse, milk-tea, fruit-tea, general beverage, and dessert drink categories were excluded from the Amap-based functional-zone classification before functional-category proportions were calculated. This exclusion was used to reduce mechanical overlap between the functional context and the Dianping-based coffee–tea venue indicators.

A grid cell was assigned to a dominant functional zone when the largest category share was at least 0.40. The dominant label was assigned to the category with the largest within-grid proportion. If no category reached 0.40, the grid cell was classified as Mixed-use (hereafter Mixed). Grids with insufficient functional information were treated as missing and were excluded from functional-zone comparisons.

For the main contextual comparisons, annual functional-zone labels were first generated under the main FICI dominant-share threshold of 0.40. The period-level functional-zone label used in the main cross-tabulated analyses was then derived from the annual labels. A grid was considered valid for functional-zone analysis only when it had at least four non-missing annual functional-zone labels during 2018–2024. For such grids, the final period-level functional zone was assigned as the modal annual functional-zone label across valid years. This procedure follows the same period-level functional-zone construction used for the main functional-zone contextual comparisons reported in Fig. 6 and Tables S7–S8, and for the functional-zone threshold sensitivity analysis reported in Table S15.

The same procedure was applied identically in Jinan and Qingdao to ensure direct comparability between the two cities. Because the functional layer was constructed from a separate POI source and excluded coffee/tea/beverage-related categories, it was not used to define CR, HI, CTI, cultural zones, or trajectory types.

# **S4.3 Interpretation of the dominant-share rule**

The 0.40 threshold was used as an operational rule to distinguish grids with a relatively clear prevailing function from grids with more heterogeneous functional composition. It does not imply that a grid becomes purely commercial, residential, educational, or tourism-oriented once one category exceeds 40 percent. Rather, it identifies the prevailing functional context of a grid while still allowing substantial minority shares from other categories.

Likewise, the Mixed category should not be interpreted as residual noise. It represents multifunctional urban settings in which no single function is sufficiently dominant under the operational rule. In substantive terms, Mixed grids capture more heterogeneous urban activity environments in which coffee–tea cultural spaces may be embedded.

The 0.40 threshold was retained in the main analysis because it provides a middle position between a more permissive rule, which would classify a larger number of borderline grids as single-function dominant, and a stricter rule, which would move a larger number of such grids into the Mixed-use category. The threshold should therefore be understood as an analytical classification rule designed to support interpretable contextual comparison rather than as a natural boundary in urban function.

# **S4.4 Threshold-sensitivity analysis under alternative dominant-share settings**

Because the dominant-share threshold is an operational analytical rule rather than a natural constant, a threshold-sensitivity analysis was conducted under alternative dominant-share settings. In addition to the main threshold of 0.40, alternative thresholds of 0.35 and 0.45 were examined.

Using the same harmonized category counts and the same main study-area masks, all eligible grids were reclassified under these alternative settings. The purpose of this analysis was not to identify an “optimal” threshold statistically, but to assess whether the substantive interpretation of functional embedding remained stable under slightly weaker or slightly stricter dominance rules.

The sensitivity analysis evaluated whether changing the threshold materially altered: first, the overall proportional distribution of functional zones in each city; and second, the main contextual relationships between functional zones and cultural zones, as well as between functional zones and five-category trajectory types.

## **S4.4.1 Sensitivity-analysis procedure**

For each threshold setting, the analysis used the corresponding annual FICI-based functional-zone fields generated in the final reproducibility workflow: func_zone_t035, func_zone_t040, and func_zone_t045. The period-level functional-zone label was then derived separately for each threshold using the same rule as the main functional-zone analysis: grids were required to have at least four non-missing annual functional-zone labels, and the final period-level functional zone was assigned as the modal annual label across valid years. Thus, the sensitivity analysis changed only the FICI dominant-share threshold while keeping the annual-to-period aggregation rule, study-area mask, fixed grid framework, and downstream cross-tabulation procedure consistent.

The resulting threshold-specific functional-zone layers were then used in three steps. First, the proportional distribution of Commercial, Residential, Education, Tourism, and Mixed-use grids was recalculated separately for Jinan and Qingdao. Second, the threshold-specific functional-zone layer was cross-tabulated with period-aggregated cultural zones. Third, the threshold-specific functional-zone layer was cross-tabulated with five-category trajectory types.

These repeated comparisons were used to assess whether the major contextual embedding patterns remained substantively similar across thresholds. The FICI threshold-sensitivity results are reported in Table S15a–S15e.

## **S4.4.2 Interpretation of sensitivity results**

If the main conclusions remained similar under the 0.35, 0.40, and 0.45 threshold settings, the functional-zoning framework was interpreted as substantively robust. If some marginal category shares changed but the dominant contextual relationships remained similar, these differences were interpreted as threshold-sensitive variation in classification detail rather than as a change in the substantive pattern of functional embedding.

In the present study, the main effect of varying the threshold was to change how borderline grids were partitioned between single dominant categories and Mixed-use. Lower thresholds classified more grids as dominant Commercial, Residential, Education, or Tourism, whereas higher thresholds increased the size of the Mixed-use category. Although exact functional-zone labels were moderately sensitive to the threshold, the associations between functional zones and cultural zones, as well as between functional zones and trajectory types, remained statistically significant across the 0.35, 0.40, and 0.45 settings. On this basis, 0.40 was retained as the main threshold setting because it provided an analytically balanced and substantively interpretable classification, while the sensitivity test supports an association-robust rather than label-invariant interpretation.

# **S4.5 Functional zoning in contextual comparisons**

Functional zones were used only in grid-level contextual comparisons. Specifically, the functional-zoning layer was compared with cultural zones, defined from period-aggregated CR in S2 Appendix, and with five-category trajectory types, defined from annual CR sequences in S3 Appendix.

These comparisons were used to assess whether period-aggregated spatial cultural orientation and temporal pathway types were differentially embedded across urban functional contexts. Functional zones were not used to define cultural indicators, trajectory types, or study-area masks. Nor were they treated as direct causal predictors. Instead, they served as empirical contextual descriptors for interpreting how coffee–tea venue-pattern change was situated across residential, commercial, educational, tourism-oriented, and mixed-use urban settings.

All contextual comparisons were conducted at the grid level within the main study-area mask. Comparisons between functional zones and cultural zones were restricted to grids with both a valid functional-zone label and a valid period-aggregated cultural-zone label. Comparisons between functional zones and trajectory types were restricted to grids with both a valid functional-zone label and an eligible trajectory label.

## **S4.5.1 Cross-tabulated summaries**

To facilitate comparison across functional contexts, cross-tabulations were summarized primarily using row percentages. In the functional zone by cultural zone analysis, row percentages show the proportional composition of tea-dominant, hybrid, and coffee-dominant grids within each functional-zone category. In the functional zone by trajectory-type analysis, row percentages show the proportional composition of stable tea, stable hybrid, stable coffee, tea rising, and coffee rising grids within each functional-zone category.

This row-standardized presentation allows direct comparison of contextual embedding patterns across functional categories even when the underlying numbers of grids differ between categories or between cities. The main functional-zone cross-tabulated summaries are reported in Table S7.

## **S4.5.2 Statistical tests, residuals, and effect sizes**

Associations between functional zones and cultural zones, and between functional zones and trajectory types, were assessed using chi-square tests of independence. These tests were used to evaluate whether the observed distributions differed from what would be expected under statistical independence. Formal chi-square tests and Cramér’s V effect sizes are reported in Table S8.

To identify which specific category combinations contributed most strongly to the overall association, adjusted standardized residuals were examined. Positive residuals indicate combinations that occurred more frequently than expected under independence, whereas negative residuals indicate combinations that occurred less frequently than expected. These residuals were used to support the interpretation of the heatmaps presented in the main text and supplementary figures.

Because chi-square significance can be influenced by sample size, association strength was summarized in parallel using Cramér’s V. In the present study, effect sizes were interpreted primarily as descriptive measures of the relative strength of contextual embedding rather than as evidence of causal determination.

Because grid cells are spatially structured rather than fully independent observations, these inferential statistics were interpreted cautiously and used primarily as descriptive evidence of association, together with Cramér’s V, standardized residuals, and mapped spatial patterns.

# **S4.6 Interpretation boundaries**

The resulting functional layer was interpreted as representing prevailing urban activity context rather than formal land-use designation or statutory zoning. Functional zones in this study therefore represent empirical contextual descriptors derived from POI composition, not formal administrative land-use categories.

Several limitations should be noted. First, functional classification depends on platform-based POI data and is therefore subject to uncertainty arising from category updates, coding changes, delayed platform updates, and residual recording error. Second, threshold-based dominant-zone assignment necessarily simplifies more complex within-grid mixtures. Even when a grid is classified as dominant Commercial or dominant Tourism, other functions may still be substantially present. Third, contextual comparisons based on functional zones are descriptive and comparative; they support interpretation of spatial embedding patterns but do not by themselves establish causal effects of urban function on coffee–tea cultural change.

In addition, because adjacent grid cells may exhibit spatial autocorrelation, inferential statistics based on grid counts should be interpreted cautiously and read together with effect sizes and mapped spatial patterns. The use of a fixed 500 m × 500 m grid also shapes the degree to which fine-grained functional heterogeneity can be detected. For these reasons, the results derived from functional zoning should be interpreted conservatively as evidence of patterned contextual embedding rather than formal land-use determination or causal mechanism.

# **S4.7 Relationship to the main analytical constructs**

Functional zones were analytically separate from the cultural indicators and trajectory types. CR, HI, and CTI describe coffee–tea composition and change. Cultural zones summarize long-run spatial orientation based on period-aggregated CR. Trajectory types summarize broader temporal pathways based on annual CR sequences. Functional zones, by contrast, describe the broader urban activity context in which those cultural patterns were situated.

This distinction is important because a grid’s prevailing functional context does not mechanically determine its coffee–tea cultural orientation or its temporal pathway. A tourism-oriented grid may be coffee-dominant, hybrid, or tea-dominant. Likewise, a mixed-use grid may show stable coexistence, directional transition, or stable dominance by one category. Functional zoning therefore supports interpretation of where coffee–tea cultural patterns are embedded, but it should not be treated as interchangeable with the cultural indicators themselves.

# **S4.8 Reproducibility notes**

The functional-zoning framework is reproducible given the Amap-derived harmonized functional categories defined in S1 Appendix, the exclusion of coffee-shop, teahouse, milk-tea, fruit-tea, general beverage, and dessert drink categories from the functional-zone calculation, the main city-specific study-area masks defined in S1 Appendix, the fixed 500 m × 500 m grid framework, the main dominant-share rule based on the 0.40 threshold, and the alternative threshold settings of 0.35 and 0.45 used in the sensitivity analysis.

Given the same Amap-derived grid-level functional-category counts, the same exclusion rules, the same study-area masks, and the same threshold rules, the resulting functional-zone labels are deterministic and reproducible. Their substantive interpretation, however, remains subject to the general limitations of POI-based classification, cross-platform data differences, and fixed-grid analysis described above.
