# Supplementary material for: Comparative dynamics of coffee–tea cultural spaces in two Chinese cities: Evidence from Qingdao and Jinan, 2018–2024
Source: PLoS One. 2026 Aug 3;21(8):e0355398. doi: 10.1371/journal.pone.0355398 (PMC13432132; doi:10.1371/journal.pone.0355398)
Supplement: S5 Appendix — (DOCX) [file pone.0355398.s009.docx]

**S5 Appendix. Sensitivity and supplementary robustness analyses**

# **S5.1 Study-area mask sensitivity analysis**

## **S5.1.1 Purpose**

The main study-area mask was constructed using POI-based urban activity information. This procedure helps exclude sparse peripheral areas with little relevance to the coffee–tea cultural analysis, but it may also exclude low-activity or newly emerging coffee–tea grids. To address this potential concern, we conducted a mask-sensitivity analysis using two alternative study-area definitions in addition to the main analytical mask.

This sensitivity test focuses on the coffee–tea cultural indicators. The definitions of Coffee Ratio (CR), Hybridization Index (HI), cultural zones, Cultural Transition Intensity (CTI), and trajectory types follow the procedures described in the main Methods and in S2–S4 Appendices. Functional-zone labels were not recalculated under the alternative masks because they were used as contextual descriptors in the main analysis and were derived from a separate functional-POI classification procedure.

## **S5.1.2 Alternative mask definitions**

Three study-area masks were compared. Mask A is the main analytical mask used in the main analysis and serves as the reference mask. Mask B is an expanded active mask that includes all 500 m × 500 m grids that contained at least one coffee-shop or teahouse POI in any year from 2018 to 2024. Mask C is a one-ring expanded mask generated by expanding the main analytical mask outward by one neighboring grid ring using Queen contiguity.

## **S5.1.3 Analytical procedure**

For each mask, we recalculated coffee-shop counts, teahouse counts, total coffee–tea POI counts, CR, HI, CTI, cultural-zone composition, and trajectory-type composition using the same rules as in the main analysis. All alternative-mask results were compared against Mask A within each city. The input file for this analysis was all_grid_year_panel_ABC_idfixed_with_func_zone_fici.gpkg. The main-mask subset was checked against the original public grid-year panel and used only to confirm consistency with the main analytical mask.

## **S5.1.4 Results**

The results are summarized in Table S10. Overall, the main temporal contrasts were generally robust to the alternative mask definitions. Across Mask A, Mask B, and Mask C, Jinan showed substantial growth in total coffee–tea POIs with only a small change in Coffee Ratio, whereas Qingdao showed limited total-count change but a clear increase in Coffee Ratio.

Under Mask A, Jinan total coffee–tea POIs increased from 1,186 in 2018 to 1,952 in 2024 (+64.59%), while CR changed only from 0.426 to 0.431. Mask B and Mask C produced the same substantive direction. In Qingdao, Mask A total coffee–tea POIs changed only from 3,130 to 3,157 (+0.86%), while CR increased from 0.493 to 0.591; this pattern was also retained under Mask B and Mask C.

Mask B increased coverage of activity-observed coffee–tea grids and therefore produced more valid grids under the activity rule, although its total number of grid cells was smaller than Mask A because Mask A also includes adjacency-expanded continuity cells. These results indicate that the main conclusions are not driven solely by the specific POI-based study-area mask. The main findings should therefore be interpreted as robust in temporal direction and overall comparative pattern, while absolute sample size and period-level composition may vary under broader activity-based mask definitions.

Table S10. Mask-sensitivity results for coffee–tea cultural indicators under alternative study-area definitions.

| **City** | **Study-area mask** | **Unique grids in mask (n)** | **Valid grids under activity rule (n)** | **Coffee POIs in 2018 (n)** | **Teahouse POIs in 2018 (n)** | **Total coffee–tea POIs in 2018 (n)** | **Coffee Ratio in 2018** | **Coffee POIs in 2024 (n)** | **Teahouse POIs in 2024 (n)** | **Total coffee–tea POIs in 2024 (n)** | **Coffee Ratio in 2024** | **Total POI change from 2018 to 2024 (%)** | **Period Coffee Ratio** | **Period Hybridization Index** | **CTI comparable grids (n)** | **Mean absolute CTI** | **Coffee-ward grids (%)** | **Tea-ward grids (%)** | **Stable grids (%)** |
| --- | --- | --- | --- | --- | --- | --- | --- | --- | --- | --- | --- | --- | --- | --- | --- | --- | --- | --- | --- |
| Jinan | Mask A | 1,742 | 458 | 505 | 681 | 1,186 | 0.426 | 842 | 1,110 | 1,952 | 0.431 | 64.59 | 0.393 | 0.370 | 299 | 0.311 | 31.44 | 35.79 | 32.78 |
|  | Mask B | 1,189 | 613 | 617 | 863 | 1,480 | 0.417 | 1,006 | 1,358 | 2,364 | 0.426 | 59.73 | 0.385 | 0.315 | 378 | 0.294 | 29.63 | 32.01 | 38.36 |
|  | Mask C | 2,051 | 458 | 506 | 681 | 1,187 | 0.426 | 846 | 1,116 | 1,962 | 0.431 | 65.29 | 0.393 | 0.370 | 299 | 0.311 | 31.44 | 35.79 | 32.78 |
| Qingdao | Mask A | 3,656 | 964 | 1,542 | 1,588 | 3,130 | 0.493 | 1,866 | 1,291 | 3,157 | 0.591 | 0.86 | 0.461 | 0.329 | 650 | 0.246 | 33.38 | 28.62 | 38 |
|  | Mask B | 2,338 | 1,365 | 1,681 | 2,207 | 3,888 | 0.432 | 2,161 | 1,723 | 3,884 | 0.556 | -0.10 | 0.389 | 0.283 | 821 | 0.241 | 31.79 | 26.80 | 41.41 |
|  | Mask C | 4,365 | 964 | 1,546 | 1,592 | 3,138 | 0.493 | 1,875 | 1,302 | 3,177 | 0.590 | 1.24 | 0.461 | 0.329 | 650 | 0.246 | 33.38 | 28.62 | 38 |

Note. Mask A refers to the main analytical mask. Mask B refers to the expanded active mask, which includes grids with coffee-shop or teahouse activity during 2018–2024. Mask C refers to the one-ring expanded mask based on Queen contiguity. Mask B is expanded in terms of coffee–tea activity coverage rather than total spatial-mask size; because Mask A includes adjacency-expanded continuity cells, its total number of grid cells may be larger than Mask B. CR is Coffee Ratio. HI is Hybridization Index. CTI is Cultural Transition Intensity. Functional-zone labels were not recalculated in the mask-sensitivity analysis.

# **S5.2 CR-threshold sensitivity analysis**

The primary analysis classified coffee–tea cultural zones using the Coffee Ratio (CR). In the main specification, grids with CR values at or below 0.30 were classified as tea-dominant, grids with CR values at or above 0.70 were classified as coffee-dominant, and grids between these thresholds were classified as hybrid. Because this threshold choice may involve a degree of arbitrariness, we conducted a CR-threshold sensitivity analysis to evaluate whether the main cultural-zone findings were dependent on this specific cutoff.

We tested three threshold specifications: 0.25/0.75, 0.30/0.70, and 0.35/0.65. The analysis used the same valid grid set as the main cultural-zone analysis, including 458 valid grids in Jinan and 964 valid grids in Qingdao. The results are reported in Table S11.

The results show that the absolute proportions of cultural-zone types changed as expected when the threshold pair was adjusted. When the hybrid interval was widened from 0.30/0.70 to 0.25/0.75, the hybrid-grid share increased from 32.75% to 40.17% in Jinan and from 26.14% to 32.26% in Qingdao. When the hybrid interval was narrowed from 0.30/0.70 to 0.35/0.65, the hybrid-grid share decreased to 24.45% in Jinan and 18.46% in Qingdao.

Although the absolute proportions varied with the threshold setting, the main cross-city pattern remained stable across all tested specifications. Under the main 0.30/0.70 specification, Jinan had 208 tea-dominant grids (45.41%), 150 hybrid grids (32.75%), and 100 coffee-dominant grids (21.83%). Qingdao had 394 tea-dominant grids (40.87%), 252 hybrid grids (26.14%), and 318 coffee-dominant grids (32.99%). Across all threshold specifications, Jinan retained higher tea-dominant and hybrid shares, whereas Qingdao retained a higher coffee-dominant share. These results indicate that the main comparative conclusion regarding the different coffee–tea spatial structures of the two cities is not driven by the specific 0.30/0.70 threshold pair used in the primary analysis.

Table S11. CR-threshold sensitivity of cultural-zone classification.

| **City** | **CR threshold setting** | **Lower threshold** | **Upper threshold** | **Cultural zone** | **Grid count (n)** | **Total valid grids (n)** | **Grid percentage (%)** | **Main specification grid count (n)** | **Main specification grid percentage (%)** | **Difference from main specification (percentage points)** |
| --- | --- | --- | --- | --- | --- | --- | --- | --- | --- | --- |
| Jinan | 0.25/0.75 | 0.250 | 0.750 | Tea-dominant | 187 | 458 | 40.83 | 208 | 45.41 | -4.59 |
|  | 0.25/0.75 | 0.250 | 0.750 | Hybrid | 184 | 458 | 40.17 | 150 | 32.75 | 7.42 |
|  | 0.25/0.75 | 0.250 | 0.750 | Coffee-dominant | 87 | 458 | 19 | 100 | 21.83 | -2.84 |
|  | 0.30/0.70 | 0.300 | 0.700 | Tea-dominant | 208 | 458 | 45.41 | 208 | 45.41 | 0 |
|  | 0.30/0.70 | 0.300 | 0.700 | Hybrid | 150 | 458 | 32.75 | 150 | 32.75 | 0 |
|  | 0.30/0.70 | 0.300 | 0.700 | Coffee-dominant | 100 | 458 | 21.83 | 100 | 21.83 | 0 |
|  | 0.35/0.65 | 0.350 | 0.650 | Tea-dominant | 227 | 458 | 49.56 | 208 | 45.41 | 4.15 |
|  | 0.35/0.65 | 0.350 | 0.650 | Hybrid | 112 | 458 | 24.45 | 150 | 32.75 | -8.30 |
|  | 0.35/0.65 | 0.350 | 0.650 | Coffee-dominant | 119 | 458 | 25.98 | 100 | 21.83 | 4.15 |
| Qingdao | 0.25/0.75 | 0.250 | 0.750 | Tea-dominant | 362 | 964 | 37.55 | 394 | 40.87 | -3.32 |
|  | 0.25/0.75 | 0.250 | 0.750 | Hybrid | 311 | 964 | 32.26 | 252 | 26.14 | 6.12 |
|  | 0.25/0.75 | 0.250 | 0.750 | Coffee-dominant | 291 | 964 | 30.19 | 318 | 32.99 | -2.80 |
|  | 0.30/0.70 | 0.300 | 0.700 | Tea-dominant | 394 | 964 | 40.87 | 394 | 40.87 | 0 |
|  | 0.30/0.70 | 0.300 | 0.700 | Hybrid | 252 | 964 | 26.14 | 252 | 26.14 | 0 |
|  | 0.30/0.70 | 0.300 | 0.700 | Coffee-dominant | 318 | 964 | 32.99 | 318 | 32.99 | 0 |
|  | 0.35/0.65 | 0.350 | 0.650 | Tea-dominant | 428 | 964 | 44.40 | 394 | 40.87 | 3.53 |
|  | 0.35/0.65 | 0.350 | 0.650 | Hybrid | 178 | 964 | 18.46 | 252 | 26.14 | -7.68 |
|  | 0.35/0.65 | 0.350 | 0.650 | Coffee-dominant | 358 | 964 | 37.14 | 318 | 32.99 | 4.15 |

Note. Percentages were calculated using the same valid grid set as the main cultural-zone analysis. The main specification used the 0.30/0.70 threshold pair. Grids with CR values at or below the lower threshold were classified as tea-dominant, grids with CR values at or above the upper threshold were classified as coffee-dominant, and intermediate grids were classified as hybrid. The source CR variable was period-level CR.

# **S5.3 Trajectory-classification sensitivity analysis**

We further examined whether the trajectory-type classification was sensitive to alternative CR threshold settings. The main analysis classified grid-level coffee–tea trajectories into five types: stable tea, stable hybrid, stable coffee, tea rising, and coffee rising. Because the stable trajectory categories partly depend on the CR thresholds used to distinguish tea-dominant, hybrid, and coffee-dominant states, it is necessary to test whether the trajectory results remain stable under alternative threshold specifications.

Using the same three threshold pairs as in the CR-threshold sensitivity analysis, namely 0.25/0.75, 0.30/0.70, and 0.35/0.65, we recalculated the trajectory type for each valid grid. The analysis used the same valid grid set as the main trajectory analysis, including 458 valid grids in Jinan and 964 valid grids in Qingdao. The 0.30/0.70 specification was treated as the main reference classification. The stability rate was defined as the percentage of grids retaining the same trajectory type as in the main specification. The results are reported in Table S12.

The trajectory-classification results were highly robust to the alternative CR threshold settings. In Jinan, 97.82% of grids retained the same trajectory type under both the 0.25/0.75 and 0.35/0.65 specifications. In Qingdao, 98.24% of grids retained the same trajectory type under the 0.25/0.75 specification, and 97.20% retained the same trajectory type under the 0.35/0.65 specification. This means that only 10 grids in Jinan and 17 to 27 grids in Qingdao changed trajectory category when the thresholds were adjusted.

The changes mainly occurred among the stable trajectory categories. The directional rising categories remained unchanged across all tested threshold specifications. In Jinan, tea-rising grids remained at 96 and coffee-rising grids remained at 94 under all three threshold settings. In Qingdao, tea-rising grids remained at 145 and coffee-rising grids remained at 190 under all three threshold settings. This pattern is consistent with the trajectory-classification rule, because the tested threshold pairs mainly affected the subdivision of stable tea, stable hybrid, and stable coffee trajectories. Overall, the trajectory sensitivity analysis shows that the main trajectory findings are not driven by the specific 0.30/0.70 threshold pair used in the primary analysis.

Table S12. Trajectory-type sensitivity under alternative CR thresholds.

| **City** | **CR threshold setting** | **Lower threshold** | **Upper threshold** | **Trajectory type** | **Grid count (n)** | **Total valid grids (n)** | **Grid percentage (%)** | **Main specification grid count (n)** | **Main specification grid percentage (%)** | **Difference from main specification (percentage points)** | **Same as main specification (n)** | **Changed grids relative to main specification (n)** | **Trajectory stability relative to main specification (%)** |
| --- | --- | --- | --- | --- | --- | --- | --- | --- | --- | --- | --- | --- | --- |
| Jinan | 0.25/0.75 | 0.250 | 0.750 | Stable tea | 155 | 458 | 33.84 | 157 | 34.28 | -0.44 | 448 | 10 | 97.82 |
|  | 0.25/0.75 | 0.250 | 0.750 | Stable hybrid | 52 | 458 | 11.35 | 42 | 9.17 | 2.18 | 448 | 10 | 97.82 |
|  | 0.25/0.75 | 0.250 | 0.750 | Stable coffee | 61 | 458 | 13.32 | 69 | 15.07 | -1.75 | 448 | 10 | 97.82 |
|  | 0.25/0.75 | 0.250 | 0.750 | Tea-rising | 96 | 458 | 20.96 | 96 | 20.96 | 0 | 448 | 10 | 97.82 |
|  | 0.25/0.75 | 0.250 | 0.750 | Coffee-rising | 94 | 458 | 20.52 | 94 | 20.52 | 0 | 448 | 10 | 97.82 |
|  | 0.30/0.70 | 0.300 | 0.700 | Stable tea | 157 | 458 | 34.28 | 157 | 34.28 | 0 | 458 | 0 | 100 |
|  | 0.30/0.70 | 0.300 | 0.700 | Stable hybrid | 42 | 458 | 9.17 | 42 | 9.17 | 0 | 458 | 0 | 100 |
|  | 0.30/0.70 | 0.300 | 0.700 | Stable coffee | 69 | 458 | 15.07 | 69 | 15.07 | 0 | 458 | 0 | 100 |
|  | 0.30/0.70 | 0.300 | 0.700 | Tea-rising | 96 | 458 | 20.96 | 96 | 20.96 | 0 | 458 | 0 | 100 |
|  | 0.30/0.70 | 0.300 | 0.700 | Coffee-rising | 94 | 458 | 20.52 | 94 | 20.52 | 0 | 458 | 0 | 100 |
|  | 0.35/0.65 | 0.350 | 0.650 | Stable tea | 164 | 458 | 35.81 | 157 | 34.28 | 1.53 | 448 | 10 | 97.82 |
|  | 0.35/0.65 | 0.350 | 0.650 | Stable hybrid | 32 | 458 | 6.99 | 42 | 9.17 | -2.18 | 448 | 10 | 97.82 |
|  | 0.35/0.65 | 0.350 | 0.650 | Stable coffee | 72 | 458 | 15.72 | 69 | 15.07 | 0.66 | 448 | 10 | 97.82 |
|  | 0.35/0.65 | 0.350 | 0.650 | Tea-rising | 96 | 458 | 20.96 | 96 | 20.96 | 0 | 448 | 10 | 97.82 |
|  | 0.35/0.65 | 0.350 | 0.650 | Coffee-rising | 94 | 458 | 20.52 | 94 | 20.52 | 0 | 448 | 10 | 97.82 |
| Qingdao | 0.25/0.75 | 0.250 | 0.750 | Stable tea | 300 | 964 | 31.12 | 307 | 31.85 | -0.73 | 947 | 17 | 98.24 |
|  | 0.25/0.75 | 0.250 | 0.750 | Stable hybrid | 103 | 964 | 10.68 | 86 | 8.92 | 1.76 | 947 | 17 | 98.24 |
|  | 0.25/0.75 | 0.250 | 0.750 | Stable coffee | 226 | 964 | 23.44 | 236 | 24.48 | -1.04 | 947 | 17 | 98.24 |
|  | 0.25/0.75 | 0.250 | 0.750 | Tea-rising | 145 | 964 | 15.04 | 145 | 15.04 | 0 | 947 | 17 | 98.24 |
|  | 0.25/0.75 | 0.250 | 0.750 | Coffee-rising | 190 | 964 | 19.71 | 190 | 19.71 | 0 | 947 | 17 | 98.24 |
|  | 0.30/0.70 | 0.300 | 0.700 | Stable tea | 307 | 964 | 31.85 | 307 | 31.85 | 0 | 964 | 0 | 100 |
|  | 0.30/0.70 | 0.300 | 0.700 | Stable hybrid | 86 | 964 | 8.92 | 86 | 8.92 | 0 | 964 | 0 | 100 |
|  | 0.30/0.70 | 0.300 | 0.700 | Stable coffee | 236 | 964 | 24.48 | 236 | 24.48 | 0 | 964 | 0 | 100 |
|  | 0.30/0.70 | 0.300 | 0.700 | Tea-rising | 145 | 964 | 15.04 | 145 | 15.04 | 0 | 964 | 0 | 100 |
|  | 0.30/0.70 | 0.300 | 0.700 | Coffee-rising | 190 | 964 | 19.71 | 190 | 19.71 | 0 | 964 | 0 | 100 |
|  | 0.35/0.65 | 0.350 | 0.650 | Stable tea | 316 | 964 | 32.78 | 307 | 31.85 | 0.93 | 937 | 27 | 97.20 |
|  | 0.35/0.65 | 0.350 | 0.650 | Stable hybrid | 59 | 964 | 6.12 | 86 | 8.92 | -2.80 | 937 | 27 | 97.20 |
|  | 0.35/0.65 | 0.350 | 0.650 | Stable coffee | 254 | 964 | 26.35 | 236 | 24.48 | 1.87 | 937 | 27 | 97.20 |
|  | 0.35/0.65 | 0.350 | 0.650 | Tea-rising | 145 | 964 | 15.04 | 145 | 15.04 | 0 | 937 | 27 | 97.20 |
|  | 0.35/0.65 | 0.350 | 0.650 | Coffee-rising | 190 | 964 | 19.71 | 190 | 19.71 | 0 | 937 | 27 | 97.20 |

Note. This table reports the number and percentage of grid-level trajectory types under three CR threshold specifications and compares each alternative classification with the main 0.30/0.70 specification. The stability rate indicates the percentage of grids retaining the same trajectory type as in the main analysis.

# **S5.4 Annual dynamics and short-term fluctuation checks for CTI**

Because the Cultural Transition Intensity (CTI) indicator is calculated from endpoint differences, we further examined whether the CTI results were strongly affected by endpoint-year selection or by short-term annual fluctuations in cultural-zone classification. This check was designed to ensure that the main CTI interpretation did not depend only on a single baseline or terminal year.

First, Table S9 reports endpoint-sensitivity checks comparing the main 2018–2024 CTI window with alternative endpoint specifications of 2018–2022 and 2018–2023. These checks evaluate whether the main endpoint interpretation is sensitive to choosing 2024 as the terminal year. For the 2018–2022 comparison, Pearson correlations with the main 2018–2024 CTI were 0.842 in Jinan and 0.757 in Qingdao, with direction-reversal rates of 5.88% and 7.80%, respectively. For the 2018–2023 comparison, consistency was stronger: Pearson correlations increased to 0.947 in Jinan and 0.891 in Qingdao, while direction-reversal rates decreased to 3.51% and 4.42%, respectively.

In addition, the response-evidence files generated by notebook 08 provide supplementary endpoint-window checks under the main CTI-valid sample. These additional checks are summarized in Table S13 together with annual dynamics, adjacent-year transition matrices, and short-term reversal counts. Therefore, Table S9 provides the formal endpoint-sensitivity table, whereas Table S13 provides a concise robustness-summary table for the revised response package.

Second, we examined annual dynamics using year-by-year city-level indicators, including annual coffee-shop counts, teahouse counts, total coffee–tea POI counts, aggregate CR, aggregate HI, mean grid-level CR, mean grid-level HI, and year-to-year changes in aggregate CR and HI. This check provides a temporal context for interpreting the endpoint-based CTI results. The annual dynamic output is retained as RE_annual_city_dynamics_main_mask.csv in the reproducibility package and is used as response evidence rather than as a replacement for the original annual summary table in the manuscript.

Third, we constructed adjacent-year cultural-zone transition matrices to examine whether cultural-zone changes were gradual and persistent or dominated by unstable year-to-year switching. For each pair of adjacent years, grids with valid coffee–tea activity in both years were used to calculate transitions among tea-dominant, hybrid, and coffee-dominant zones. This check helps identify whether the long-term CTI pattern reflects repeated directional movement or isolated one-year changes. The adjacent-year transition output is retained as RE_adjacent_year_zone_transition_matrices.csv.

Finally, we identified short-term reversals in three-year windows. A short-term reversal was defined as a sequence in which the first and third years shared the same cultural-zone label, while the middle year temporarily shifted to another zone. Across the tested windows, the total reversal percentage remained limited, with the maximum city-window reversal rate equal to 6.13% (20 reversals among 326 valid three-year sequences in Jinan, 2021-2022-2023). The short-term reversal output is retained as RE_short_term_reversal_counts.csv.

Overall, these annual dynamics and short-term fluctuation checks support the robustness of the CTI-based interpretation. The endpoint-sensitivity results show moderate to strong consistency between the main 2018–2024 CTI and alternative endpoint specifications, with stronger consistency for the 2018–2023 comparison than for the 2018–2022 comparison. Therefore, the revised analysis avoids treating CTI as a complete substitute for annual dynamics and instead interprets CTI as a concise descriptive measure of endpoint directional change.

Table S13. Summary of robustness findings.

| **Robustness check** | **Main specification** | **Alternative specification(s)** | **Evidence table(s)** | **Key diagnostic/result** | **Robustness assessment** | **Implication for manuscript conclusion** |
| --- | --- | --- | --- | --- | --- | --- |
| Study-area mask sensitivity | Mask A: main analytical mask | Mask B: expanded activity-inclusive mask; Mask C: one-ring expanded mask | Table S10 | Main temporal contrasts were preserved across Mask A, Mask B, and Mask C: Jinan showed substantial total POI growth with little change in Coffee Ratio, whereas Qingdao showed a clear Coffee Ratio increase with limited total-count change. | Main city-level temporal conclusions were robust to alternative study-area masks. | Retain the main interpretation; note that mask choice affects sample size but not the direction of the main findings. |
| CR threshold sensitivity | Cultural-zone thresholds: CR ≤ 0.30, 0.30 < CR < 0.70, CR ≥ 0.70 | CR thresholds 0.25/0.75 and 0.35/0.65 | Table S11 | Jinan: maximum absolute zone-share difference = 8.30 percentage points; Qingdao: maximum absolute zone-share difference = 7.68 percentage points | Cultural-zone composition changed moderately but the main inter-city contrast remained stable. | Retain the conclusion that Jinan is more tea/hybrid-oriented, while Qingdao combines a city-level coffee shift with substantial tea-dominant grid presence. |
| Trajectory-classification sensitivity | Trajectory classification based on the main CR thresholds 0.30/0.70 | Trajectory classification under CR thresholds 0.25/0.75 and 0.35/0.65 | Table S12 | Jinan: minimum stability = 97.82%; Qingdao: minimum stability = 97.20% | Trajectory classifications were highly stable under alternative CR thresholds. | Retain the trajectory-based interpretation of stable, coffee-rising, and tea-rising grids. |
| CTI annual dynamics and endpoint-window evidence | CTI based on the 2018→2024 endpoint change in Coffee Ratio | Annual dynamics, adjacent-year changes, short-term reversals, and alternative endpoint windows | Response-evidence tables generated by notebook 08 | Generated evidence: annual city dynamics, short-term reversal counts, endpoint-window sensitivity. Jinan: alternative-window comparable grids = 285–292; Qingdao: alternative-window comparable grids = 611–635. Endpoint-window evidence was restricted to grids with the main CTI-eligible grid-summary sample; alternative windows additionally require valid CR at both alternative endpoint years. | Endpoint CTI was supplemented with annual and short-term evidence to clarify that endpoint change does not conceal the full temporal pattern. | Retain CTI as an endpoint-change indicator, with annual dynamics reported as supporting evidence rather than a replacement measure. |
| Spatial autocorrelation check | Grid-level CR, HI, and CTI statistics without spatial model correction | Global Moran's I using Queen contiguity weights | Table S14 | CR/HI significant cases = 31/32. Jinan CTI: Moran's I = 0.107, p = 0.017, n = 299, significant Qingdao CTI: Moran's I = 0.001, p = 0.459, n = 650, not significant Non-significant/weak status-indicator case(s): Jinan CR 2019 p = 0.072. | CR and HI mostly showed positive spatial clustering; CTI spatial autocorrelation was weak and city-specific, significant in Jinan but not in Qingdao under the main CTI sample. | Retain the descriptive interpretation while explicitly acknowledging spatial clustering in status indicators and city-specific spatial dependence in CTI. |
| Functional-zone Fi-Ci threshold sensitivity | Fi-Ci functional-zone threshold = 0.40 | Fi-Ci functional-zone thresholds = 0.35 and 0.45 | Table S15a–S15e | Jinan: minimum classification stability = 77.36%; Qingdao: minimum classification stability = 76.86%; All tested functional-zone associations remained statistically significant = True. | Exact functional-zone labels were moderately sensitive to the Fi-Ci threshold, mainly through shifts into or out of Mixed, but functional-cultural associations remained significant across thresholds. | Retain the functional-embedding interpretation, while describing the Fi-Ci threshold test as association-robust rather than label-invariant. |

Note. This table summarizes the main sensitivity and supplementary robustness checks added in the revision. The detailed formal outputs are provided in Tables S10–S15. Additional CTI annual-dynamics, adjacent-year transition, short-term reversal, and endpoint-window files are retained as response-evidence files in the revised reproducibility package.

# **S5.5 Spatial autocorrelation checks**

To examine whether the gridded coffee–tea indicators were spatially independent or spatially structured, we conducted global Moran’s I tests for the main cultural indicators. The tests were performed separately for Jinan and Qingdao using a Queen contiguity spatial weight matrix with row standardization. Annual CR and HI were tested for each year from 2018 to 2024. Period-level CR, period-level HI, and CTI were also tested to evaluate the spatial structure of long-run composition, long-run hybridization, and endpoint transition intensity. Only grids with valid indicator values were included in each test; therefore, the number of valid grids varies across cities, years, and indicators. The results are reported in Table S14.

The results show that long-run coffee–tea composition and hybridization were spatially structured in both cities. Period-level CR showed significant positive spatial autocorrelation in Jinan (Moran’s I = 0.1454, p = 0.001) and Qingdao (Moran’s I = 0.2508, p = 0.001). Period-level HI also showed significant positive spatial autocorrelation in Jinan (Moran’s I = 0.2065, p = 0.001) and Qingdao (Moran’s I = 0.1942, p = 0.001). These results support the interpretation that the long-run spatial composition and mixing of coffee–tea cultural spaces were not randomly distributed, but instead exhibited spatial clustering.

The annual results were broadly consistent with the period-level results. In Qingdao, annual CR and HI both showed significant positive spatial autocorrelation in all years from 2018 to 2024. In Jinan, annual HI was also significantly positively spatially autocorrelated in all years. Annual CR in Jinan was significant in most years, with the only exception being 2019, where the result was weak and not conventionally significant (Moran’s I = 0.0608, p = 0.072). Overall, these annual checks support the robustness of the long-run spatial-structure interpretation for CR and HI.

In contrast, CTI showed weaker and more city-specific spatial autocorrelation than CR and HI. In Jinan, CTI showed a small but statistically significant positive spatial autocorrelation (Moran’s I = 0.1072, p = 0.017), while in Qingdao it showed no significant spatial autocorrelation (Moran’s I = 0.0014, p = 0.459). This indicates that endpoint transition intensity was less consistently spatially clustered than long-run coffee–tea composition and hybridization. Therefore, CTI should be interpreted as a descriptive measure of directional change between 2018 and 2024, rather than as evidence of uniformly clustered transition intensity.

Taken together, the spatial autocorrelation checks support the main conclusion that long-run coffee–tea cultural composition and hybridization were spatially structured in both cities. At the same time, the CTI results suggest that long-run spatial structure and endpoint transition intensity reflect different aspects of coffee–tea cultural change, with CTI spatial dependence being small and city-specific. This distinction supports a more cautious interpretation of CTI and avoids overgeneralizing spatial clustering from composition and hybridization to all forms of cultural transition.

Table S14. Spatial autocorrelation results for the main cultural indicators.

| **City** | **Indicator** | **Year or period** | **Valid grids (n)** | **Moran’s I** | **p-value** | **Spatial weight matrix** | **Sample definition** | **Interpretation** |
| --- | --- | --- | --- | --- | --- | --- | --- | --- |
| Jinan | CR | 2018 | 426 | 0.1106 | 0.004 | Queen contiguity, row-standardized | Annual active grids with valid CR/HI | Significant positive spatial autocorrelation |
|  | CR | 2018-2024 | 767 | 0.1454 | 0.001 | Queen contiguity, row-standardized | Period-level grids with valid period CR/HI | Significant positive spatial autocorrelation |
|  | CR | 2019 | 434 | 0.0608 | 0.072 | Queen contiguity, row-standardized | Annual active grids with valid CR/HI | Weak positive spatial autocorrelation |
|  | CR | 2020 | 417 | 0.1294 | 0.001 | Queen contiguity, row-standardized | Annual active grids with valid CR/HI | Significant positive spatial autocorrelation |
|  | CR | 2021 | 458 | 0.1248 | 0.001 | Queen contiguity, row-standardized | Annual active grids with valid CR/HI | Significant positive spatial autocorrelation |
|  | CR | 2022 | 474 | 0.0994 | 0.004 | Queen contiguity, row-standardized | Annual active grids with valid CR/HI | Significant positive spatial autocorrelation |
|  | CR | 2023 | 522 | 0.1558 | 0.001 | Queen contiguity, row-standardized | Annual active grids with valid CR/HI | Significant positive spatial autocorrelation |
|  | CR | 2024 | 585 | 0.1453 | 0.001 | Queen contiguity, row-standardized | Annual active grids with valid CR/HI | Significant positive spatial autocorrelation |
|  | HI | 2018 | 426 | 0.1161 | 0.002 | Queen contiguity, row-standardized | Annual active grids with valid CR/HI | Significant positive spatial autocorrelation |
|  | HI | 2018-2024 | 767 | 0.2065 | 0.001 | Queen contiguity, row-standardized | Period-level grids with valid period CR/HI | Significant positive spatial autocorrelation |
|  | HI | 2019 | 434 | 0.0807 | 0.037 | Queen contiguity, row-standardized | Annual active grids with valid CR/HI | Significant positive spatial autocorrelation |
|  | HI | 2020 | 417 | 0.0813 | 0.037 | Queen contiguity, row-standardized | Annual active grids with valid CR/HI | Significant positive spatial autocorrelation |
|  | HI | 2021 | 458 | 0.1122 | 0.005 | Queen contiguity, row-standardized | Annual active grids with valid CR/HI | Significant positive spatial autocorrelation |
|  | HI | 2022 | 474 | 0.1416 | 0.003 | Queen contiguity, row-standardized | Annual active grids with valid CR/HI | Significant positive spatial autocorrelation |
|  | HI | 2023 | 522 | 0.1607 | 0.001 | Queen contiguity, row-standardized | Annual active grids with valid CR/HI | Significant positive spatial autocorrelation |
|  | HI | 2024 | 585 | 0.1638 | 0.001 | Queen contiguity, row-standardized | Annual active grids with valid CR/HI | Significant positive spatial autocorrelation |
|  | CTI | 2018-2024 endpoint | 299 | 0.1072 | 0.017 | Queen contiguity, row-standardized | Main CTI analysis sample: the main CTI-eligible grid-summary sample | Significant positive spatial autocorrelation |
| Qingdao | CR | 2018 | 969 | 0.2943 | 0.001 | Queen contiguity, row-standardized | Annual active grids with valid CR/HI | Significant positive spatial autocorrelation |
|  | CR | 2018-2024 | 1,477 | 0.2508 | 0.001 | Queen contiguity, row-standardized | Period-level grids with valid period CR/HI | Significant positive spatial autocorrelation |
|  | CR | 2019 | 970 | 0.3071 | 0.001 | Queen contiguity, row-standardized | Annual active grids with valid CR/HI | Significant positive spatial autocorrelation |
|  | CR | 2020 | 980 | 0.2908 | 0.001 | Queen contiguity, row-standardized | Annual active grids with valid CR/HI | Significant positive spatial autocorrelation |
|  | CR | 2021 | 1,008 | 0.2381 | 0.001 | Queen contiguity, row-standardized | Annual active grids with valid CR/HI | Significant positive spatial autocorrelation |
|  | CR | 2022 | 926 | 0.1369 | 0.001 | Queen contiguity, row-standardized | Annual active grids with valid CR/HI | Significant positive spatial autocorrelation |
|  | CR | 2023 | 969 | 0.1875 | 0.001 | Queen contiguity, row-standardized | Annual active grids with valid CR/HI | Significant positive spatial autocorrelation |
|  | CR | 2024 | 1,030 | 0.2108 | 0.001 | Queen contiguity, row-standardized | Annual active grids with valid CR/HI | Significant positive spatial autocorrelation |
|  | HI | 2018 | 969 | 0.1431 | 0.001 | Queen contiguity, row-standardized | Annual active grids with valid CR/HI | Significant positive spatial autocorrelation |
|  | HI | 2018-2024 | 1,477 | 0.1942 | 0.001 | Queen contiguity, row-standardized | Period-level grids with valid period CR/HI | Significant positive spatial autocorrelation |
|  | HI | 2019 | 970 | 0.1817 | 0.001 | Queen contiguity, row-standardized | Annual active grids with valid CR/HI | Significant positive spatial autocorrelation |
|  | HI | 2020 | 980 | 0.1723 | 0.001 | Queen contiguity, row-standardized | Annual active grids with valid CR/HI | Significant positive spatial autocorrelation |
|  | HI | 2021 | 1,008 | 0.2368 | 0.001 | Queen contiguity, row-standardized | Annual active grids with valid CR/HI | Significant positive spatial autocorrelation |
|  | HI | 2022 | 926 | 0.2044 | 0.001 | Queen contiguity, row-standardized | Annual active grids with valid CR/HI | Significant positive spatial autocorrelation |
|  | HI | 2023 | 969 | 0.1875 | 0.001 | Queen contiguity, row-standardized | Annual active grids with valid CR/HI | Significant positive spatial autocorrelation |
|  | HI | 2024 | 1,030 | 0.1452 | 0.001 | Queen contiguity, row-standardized | Annual active grids with valid CR/HI | Significant positive spatial autocorrelation |
|  | CTI | 2018-2024 endpoint | 650 | 0.0014 | 0.459 | Queen contiguity, row-standardized | Main CTI analysis sample: the main CTI-eligible grid-summary sample | Not significant spatial autocorrelation |

Note. Global Moran’s I tests were conducted separately by city and indicator. Annual CR and HI were tested for each year from 2018 to 2024. Period-level CR and HI were tested for 2018–2024, and CTI was tested for the endpoint change from 2018 to 2024. The spatial weight matrix was based on Queen contiguity and row standardization. Only grids with valid indicator values were included in each test.

Table S15. Functional-zone FICI threshold-sensitivity results.

Table S15a. Functional-zone composition under alternative FICI thresholds.

| **City** | **FICI threshold** | **Functional zone** | **Grid count (n)** | **Grid percentage (%)** | **Total valid grids (n)** | **Main specification percentage (%)** | **Difference from main specification (percentage points)** |
| --- | --- | --- | --- | --- | --- | --- | --- |
| Jinan | 0.350 | Commercial | 61 | 13.41 | 455 | 9.67 | 3.74 |
|  | 0.350 | Education | 98 | 21.54 | 455 | 14.95 | 6.59 |
|  | 0.350 | Mixed | 97 | 21.32 | 455 | 43.52 | -22.20 |
|  | 0.350 | Residential | 109 | 23.96 | 455 | 16.26 | 7.69 |
|  | 0.350 | Tourism | 90 | 19.78 | 455 | 15.60 | 4.18 |
|  | 0.400 | Commercial | 44 | 9.67 | 455 | 9.67 | 0 |
|  | 0.400 | Education | 68 | 14.95 | 455 | 14.95 | 0 |
|  | 0.400 | Mixed | 198 | 43.52 | 455 | 43.52 | 0 |
|  | 0.400 | Residential | 74 | 16.26 | 455 | 16.26 | 0 |
|  | 0.400 | Tourism | 71 | 15.60 | 455 | 15.60 | 0 |
|  | 0.450 | Commercial | 25 | 5.49 | 455 | 9.67 | -4.18 |
|  | 0.450 | Education | 47 | 10.33 | 455 | 14.95 | -4.62 |
|  | 0.450 | Mixed | 280 | 61.54 | 455 | 43.52 | 18.02 |
|  | 0.450 | Residential | 51 | 11.21 | 455 | 16.26 | -5.05 |
|  | 0.450 | Tourism | 52 | 11.43 | 455 | 15.60 | -4.18 |
| Qingdao | 0.350 | Commercial | 163 | 17.07 | 955 | 12.88 | 4.19 |
|  | 0.350 | Education | 203 | 21.26 | 955 | 13.30 | 7.96 |
|  | 0.350 | Mixed | 159 | 16.65 | 955 | 39.58 | -22.93 |
|  | 0.350 | Residential | 238 | 24.92 | 955 | 17.80 | 7.12 |
|  | 0.350 | Tourism | 192 | 20.10 | 955 | 16.44 | 3.66 |
|  | 0.400 | Commercial | 123 | 12.88 | 955 | 12.88 | 0 |
|  | 0.400 | Education | 127 | 13.30 | 955 | 13.30 | 0 |
|  | 0.400 | Mixed | 378 | 39.58 | 955 | 39.58 | 0 |
|  | 0.400 | Residential | 170 | 17.80 | 955 | 17.80 | 0 |
|  | 0.400 | Tourism | 157 | 16.44 | 955 | 16.44 | 0 |
|  | 0.450 | Commercial | 83 | 8.69 | 955 | 12.88 | -4.19 |
|  | 0.450 | Education | 79 | 8.27 | 955 | 13.30 | -5.03 |
|  | 0.450 | Mixed | 547 | 57.28 | 955 | 39.58 | 17.70 |
|  | 0.450 | Residential | 119 | 12.46 | 955 | 17.80 | -5.34 |
|  | 0.450 | Tourism | 127 | 13.30 | 955 | 16.44 | -3.14 |

Table S15b. Functional-zone classification stability under alternative FICI thresholds.

| **City** | **Alternative FICI threshold** | **Main FICI threshold** | **Comparable grids (n)** | **Stable classifications (n)** | **Changed classifications (n)** | **Classification stability (%)** | **Changed classification (%)** |
| --- | --- | --- | --- | --- | --- | --- | --- |
| Jinan | 0.350 | 0.400 | 455 | 352 | 103 | 77.36 | 22.64 |
| Jinan | 0.450 | 0.400 | 455 | 372 | 83 | 81.76 | 18.24 |
| Qingdao | 0.350 | 0.400 | 955 | 734 | 221 | 76.86 | 23.14 |
| Qingdao | 0.450 | 0.400 | 955 | 785 | 170 | 82.20 | 17.80 |

Table S15c. Functional zone by cultural zone under alternative FICI thresholds.

| **City** | **FICI threshold** | **Functional zone** | **Cultural zone** | **Grid count (n)** | **Functional-zone total (n)** | **Within-functional-zone percentage (%)** |
| --- | --- | --- | --- | --- | --- | --- |
| Jinan | 0.350 | Commercial | Coffee-dominant | 17 | 61 | 27.87 |
|  | 0.350 | Commercial | Hybrid | 16 | 61 | 26.23 |
|  | 0.350 | Commercial | Tea-dominant | 28 | 61 | 45.90 |
|  | 0.350 | Education | Coffee-dominant | 24 | 98 | 24.49 |
|  | 0.350 | Education | Hybrid | 27 | 98 | 27.55 |
|  | 0.350 | Education | Tea-dominant | 47 | 98 | 47.96 |
|  | 0.350 | Mixed | Coffee-dominant | 11 | 97 | 11.34 |
|  | 0.350 | Mixed | Hybrid | 43 | 97 | 44.33 |
|  | 0.350 | Mixed | Tea-dominant | 43 | 97 | 44.33 |
|  | 0.350 | Residential | Coffee-dominant | 17 | 109 | 15.60 |
|  | 0.350 | Residential | Hybrid | 31 | 109 | 28.44 |
|  | 0.350 | Residential | Tea-dominant | 61 | 109 | 55.96 |
|  | 0.350 | Tourism | Coffee-dominant | 30 | 90 | 33.33 |
|  | 0.350 | Tourism | Hybrid | 33 | 90 | 36.67 |
|  | 0.350 | Tourism | Tea-dominant | 27 | 90 | 30 |
|  | 0.400 | Commercial | Coffee-dominant | 14 | 44 | 31.82 |
|  | 0.400 | Commercial | Hybrid | 9 | 44 | 20.45 |
|  | 0.400 | Commercial | Tea-dominant | 21 | 44 | 47.73 |
|  | 0.400 | Education | Coffee-dominant | 17 | 68 | 25 |
|  | 0.400 | Education | Hybrid | 13 | 68 | 19.12 |
|  | 0.400 | Education | Tea-dominant | 38 | 68 | 55.88 |
|  | 0.400 | Mixed | Coffee-dominant | 31 | 198 | 15.66 |
|  | 0.400 | Mixed | Hybrid | 82 | 198 | 41.41 |
|  | 0.400 | Mixed | Tea-dominant | 85 | 198 | 42.93 |
|  | 0.400 | Residential | Coffee-dominant | 12 | 74 | 16.22 |
|  | 0.400 | Residential | Hybrid | 20 | 74 | 27.03 |
|  | 0.400 | Residential | Tea-dominant | 42 | 74 | 56.76 |
|  | 0.400 | Tourism | Coffee-dominant | 25 | 71 | 35.21 |
|  | 0.400 | Tourism | Hybrid | 26 | 71 | 36.62 |
|  | 0.400 | Tourism | Tea-dominant | 20 | 71 | 28.17 |
|  | 0.450 | Commercial | Coffee-dominant | 8 | 25 | 32 |
|  | 0.450 | Commercial | Hybrid | 5 | 25 | 20 |
|  | 0.450 | Commercial | Tea-dominant | 12 | 25 | 48 |
|  | 0.450 | Education | Coffee-dominant | 12 | 47 | 25.53 |
|  | 0.450 | Education | Hybrid | 8 | 47 | 17.02 |
|  | 0.450 | Education | Tea-dominant | 27 | 47 | 57.45 |
|  | 0.450 | Mixed | Coffee-dominant | 54 | 280 | 19.29 |
|  | 0.450 | Mixed | Hybrid | 105 | 280 | 37.50 |
|  | 0.450 | Mixed | Tea-dominant | 121 | 280 | 43.21 |
|  | 0.450 | Residential | Coffee-dominant | 7 | 51 | 13.73 |
|  | 0.450 | Residential | Hybrid | 14 | 51 | 27.45 |
|  | 0.450 | Residential | Tea-dominant | 30 | 51 | 58.82 |
|  | 0.450 | Tourism | Coffee-dominant | 18 | 52 | 34.62 |
|  | 0.450 | Tourism | Hybrid | 18 | 52 | 34.62 |
|  | 0.450 | Tourism | Tea-dominant | 16 | 52 | 30.77 |
| Qingdao | 0.350 | Commercial | Coffee-dominant | 33 | 163 | 20.25 |
|  | 0.350 | Commercial | Hybrid | 29 | 163 | 17.79 |
|  | 0.350 | Commercial | Tea-dominant | 101 | 163 | 61.96 |
|  | 0.350 | Education | Coffee-dominant | 61 | 203 | 30.05 |
|  | 0.350 | Education | Hybrid | 56 | 203 | 27.59 |
|  | 0.350 | Education | Tea-dominant | 86 | 203 | 42.36 |
|  | 0.350 | Mixed | Coffee-dominant | 41 | 159 | 25.79 |
|  | 0.350 | Mixed | Hybrid | 59 | 159 | 37.11 |
|  | 0.350 | Mixed | Tea-dominant | 59 | 159 | 37.11 |
|  | 0.350 | Residential | Coffee-dominant | 67 | 238 | 28.15 |
|  | 0.350 | Residential | Hybrid | 52 | 238 | 21.85 |
|  | 0.350 | Residential | Tea-dominant | 119 | 238 | 50 |
|  | 0.350 | Tourism | Coffee-dominant | 108 | 192 | 56.25 |
|  | 0.350 | Tourism | Hybrid | 56 | 192 | 29.17 |
|  | 0.350 | Tourism | Tea-dominant | 28 | 192 | 14.58 |
|  | 0.400 | Commercial | Coffee-dominant | 19 | 123 | 15.45 |
|  | 0.400 | Commercial | Hybrid | 21 | 123 | 17.07 |
|  | 0.400 | Commercial | Tea-dominant | 83 | 123 | 67.48 |
|  | 0.400 | Education | Coffee-dominant | 41 | 127 | 32.28 |
|  | 0.400 | Education | Hybrid | 33 | 127 | 25.98 |
|  | 0.400 | Education | Tea-dominant | 53 | 127 | 41.73 |
|  | 0.400 | Mixed | Coffee-dominant | 104 | 378 | 27.51 |
|  | 0.400 | Mixed | Hybrid | 116 | 378 | 30.69 |
|  | 0.400 | Mixed | Tea-dominant | 158 | 378 | 41.80 |
|  | 0.400 | Residential | Coffee-dominant | 52 | 170 | 30.59 |
|  | 0.400 | Residential | Hybrid | 40 | 170 | 23.53 |
|  | 0.400 | Residential | Tea-dominant | 78 | 170 | 45.88 |
|  | 0.400 | Tourism | Coffee-dominant | 94 | 157 | 59.87 |
|  | 0.400 | Tourism | Hybrid | 42 | 157 | 26.75 |
|  | 0.400 | Tourism | Tea-dominant | 21 | 157 | 13.38 |
|  | 0.450 | Commercial | Coffee-dominant | 13 | 83 | 15.66 |
|  | 0.450 | Commercial | Hybrid | 14 | 83 | 16.87 |
|  | 0.450 | Commercial | Tea-dominant | 56 | 83 | 67.47 |
|  | 0.450 | Education | Coffee-dominant | 24 | 79 | 30.38 |
|  | 0.450 | Education | Hybrid | 17 | 79 | 21.52 |
|  | 0.450 | Education | Tea-dominant | 38 | 79 | 48.10 |
|  | 0.450 | Mixed | Coffee-dominant | 151 | 547 | 27.61 |
|  | 0.450 | Mixed | Hybrid | 160 | 547 | 29.25 |
|  | 0.450 | Mixed | Tea-dominant | 236 | 547 | 43.14 |
|  | 0.450 | Residential | Coffee-dominant | 43 | 119 | 36.13 |
|  | 0.450 | Residential | Hybrid | 27 | 119 | 22.69 |
|  | 0.450 | Residential | Tea-dominant | 49 | 119 | 41.18 |
|  | 0.450 | Tourism | Coffee-dominant | 79 | 127 | 62.20 |
|  | 0.450 | Tourism | Hybrid | 34 | 127 | 26.77 |
|  | 0.450 | Tourism | Tea-dominant | 14 | 127 | 11.02 |

Table S15d. Functional zone by trajectory type under alternative FICI thresholds.

| **City** | **FICI threshold** | **Functional zone** | **Trajectory type** | **Grid count (n)** | **Functional-zone total (n)** | **Within-functional-zone percentage (%)** |
| --- | --- | --- | --- | --- | --- | --- |
| Jinan | 0.350 | Commercial | Coffee-rising | 8 | 61 | 13.11 |
|  | 0.350 | Commercial | Stable coffee | 14 | 61 | 22.95 |
|  | 0.350 | Commercial | Stable hybrid | 6 | 61 | 9.84 |
|  | 0.350 | Commercial | Stable tea | 25 | 61 | 40.98 |
|  | 0.350 | Commercial | Tea-rising | 8 | 61 | 13.11 |
|  | 0.350 | Education | Coffee-rising | 18 | 98 | 18.37 |
|  | 0.350 | Education | Stable coffee | 20 | 98 | 20.41 |
|  | 0.350 | Education | Stable hybrid | 7 | 98 | 7.14 |
|  | 0.350 | Education | Stable tea | 37 | 98 | 37.76 |
|  | 0.350 | Education | Tea-rising | 16 | 98 | 16.33 |
|  | 0.350 | Mixed | Coffee-rising | 28 | 97 | 28.87 |
|  | 0.350 | Mixed | Stable coffee | 7 | 97 | 7.22 |
|  | 0.350 | Mixed | Stable hybrid | 14 | 97 | 14.43 |
|  | 0.350 | Mixed | Stable tea | 30 | 97 | 30.93 |
|  | 0.350 | Mixed | Tea-rising | 18 | 97 | 18.56 |
|  | 0.350 | Residential | Coffee-rising | 23 | 109 | 21.10 |
|  | 0.350 | Residential | Stable coffee | 9 | 109 | 8.26 |
|  | 0.350 | Residential | Stable hybrid | 4 | 109 | 3.67 |
|  | 0.350 | Residential | Stable tea | 45 | 109 | 41.28 |
|  | 0.350 | Residential | Tea-rising | 28 | 109 | 25.69 |
|  | 0.350 | Tourism | Coffee-rising | 17 | 90 | 18.89 |
|  | 0.350 | Tourism | Stable coffee | 18 | 90 | 20 |
|  | 0.350 | Tourism | Stable hybrid | 11 | 90 | 12.22 |
|  | 0.350 | Tourism | Stable tea | 18 | 90 | 20 |
|  | 0.350 | Tourism | Tea-rising | 26 | 90 | 28.89 |
|  | 0.400 | Commercial | Coffee-rising | 6 | 44 | 13.64 |
|  | 0.400 | Commercial | Stable coffee | 11 | 44 | 25 |
|  | 0.400 | Commercial | Stable hybrid | 3 | 44 | 6.82 |
|  | 0.400 | Commercial | Stable tea | 19 | 44 | 43.18 |
|  | 0.400 | Commercial | Tea-rising | 5 | 44 | 11.36 |
|  | 0.400 | Education | Coffee-rising | 14 | 68 | 20.59 |
|  | 0.400 | Education | Stable coffee | 14 | 68 | 20.59 |
|  | 0.400 | Education | Stable hybrid | 2 | 68 | 2.94 |
|  | 0.400 | Education | Stable tea | 29 | 68 | 42.65 |
|  | 0.400 | Education | Tea-rising | 9 | 68 | 13.24 |
|  | 0.400 | Mixed | Coffee-rising | 44 | 198 | 22.22 |
|  | 0.400 | Mixed | Stable coffee | 21 | 198 | 10.61 |
|  | 0.400 | Mixed | Stable hybrid | 28 | 198 | 14.14 |
|  | 0.400 | Mixed | Stable tea | 61 | 198 | 30.81 |
|  | 0.400 | Mixed | Tea-rising | 44 | 198 | 22.22 |
|  | 0.400 | Residential | Coffee-rising | 16 | 74 | 21.62 |
|  | 0.400 | Residential | Stable coffee | 7 | 74 | 9.46 |
|  | 0.400 | Residential | Stable hybrid | 2 | 74 | 2.70 |
|  | 0.400 | Residential | Stable tea | 31 | 74 | 41.89 |
|  | 0.400 | Residential | Tea-rising | 18 | 74 | 24.32 |
|  | 0.400 | Tourism | Coffee-rising | 14 | 71 | 19.72 |
|  | 0.400 | Tourism | Stable coffee | 15 | 71 | 21.13 |
|  | 0.400 | Tourism | Stable hybrid | 7 | 71 | 9.86 |
|  | 0.400 | Tourism | Stable tea | 15 | 71 | 21.13 |
|  | 0.400 | Tourism | Tea-rising | 20 | 71 | 28.17 |
|  | 0.450 | Commercial | Coffee-rising | 2 | 25 | 8 |
|  | 0.450 | Commercial | Stable coffee | 6 | 25 | 24 |
|  | 0.450 | Commercial | Stable hybrid | 2 | 25 | 8 |
|  | 0.450 | Commercial | Stable tea | 11 | 25 | 44 |
|  | 0.450 | Commercial | Tea-rising | 4 | 25 | 16 |
|  | 0.450 | Education | Coffee-rising | 12 | 47 | 25.53 |
|  | 0.450 | Education | Stable coffee | 9 | 47 | 19.15 |
|  | 0.450 | Education | Stable hybrid | 0 | 47 | 0 |
|  | 0.450 | Education | Stable tea | 22 | 47 | 46.81 |
|  | 0.450 | Education | Tea-rising | 4 | 47 | 8.51 |
|  | 0.450 | Mixed | Coffee-rising | 60 | 280 | 21.43 |
|  | 0.450 | Mixed | Stable coffee | 36 | 280 | 12.86 |
|  | 0.450 | Mixed | Stable hybrid | 33 | 280 | 11.79 |
|  | 0.450 | Mixed | Stable tea | 87 | 280 | 31.07 |
|  | 0.450 | Mixed | Tea-rising | 64 | 280 | 22.86 |
|  | 0.450 | Residential | Coffee-rising | 10 | 51 | 19.61 |
|  | 0.450 | Residential | Stable coffee | 5 | 51 | 9.80 |
|  | 0.450 | Residential | Stable hybrid | 1 | 51 | 1.96 |
|  | 0.450 | Residential | Stable tea | 24 | 51 | 47.06 |
|  | 0.450 | Residential | Tea-rising | 11 | 51 | 21.57 |
|  | 0.450 | Tourism | Coffee-rising | 10 | 52 | 19.23 |
|  | 0.450 | Tourism | Stable coffee | 12 | 52 | 23.08 |
|  | 0.450 | Tourism | Stable hybrid | 6 | 52 | 11.54 |
|  | 0.450 | Tourism | Stable tea | 11 | 52 | 21.15 |
|  | 0.450 | Tourism | Tea-rising | 13 | 52 | 25 |
| Qingdao | 0.350 | Commercial | Coffee-rising | 25 | 163 | 15.34 |
|  | 0.350 | Commercial | Stable coffee | 21 | 163 | 12.88 |
|  | 0.350 | Commercial | Stable hybrid | 7 | 163 | 4.29 |
|  | 0.350 | Commercial | Stable tea | 91 | 163 | 55.83 |
|  | 0.350 | Commercial | Tea-rising | 19 | 163 | 11.66 |
|  | 0.350 | Education | Coffee-rising | 41 | 203 | 20.20 |
|  | 0.350 | Education | Stable coffee | 45 | 203 | 22.17 |
|  | 0.350 | Education | Stable hybrid | 24 | 203 | 11.82 |
|  | 0.350 | Education | Stable tea | 61 | 203 | 30.05 |
|  | 0.350 | Education | Tea-rising | 32 | 203 | 15.76 |
|  | 0.350 | Mixed | Coffee-rising | 31 | 159 | 19.50 |
|  | 0.350 | Mixed | Stable coffee | 32 | 159 | 20.13 |
|  | 0.350 | Mixed | Stable hybrid | 24 | 159 | 15.09 |
|  | 0.350 | Mixed | Stable tea | 46 | 159 | 28.93 |
|  | 0.350 | Mixed | Tea-rising | 26 | 159 | 16.35 |
|  | 0.350 | Residential | Coffee-rising | 53 | 238 | 22.27 |
|  | 0.350 | Residential | Stable coffee | 49 | 238 | 20.59 |
|  | 0.350 | Residential | Stable hybrid | 12 | 238 | 5.04 |
|  | 0.350 | Residential | Stable tea | 90 | 238 | 37.82 |
|  | 0.350 | Residential | Tea-rising | 34 | 238 | 14.29 |
|  | 0.350 | Tourism | Coffee-rising | 40 | 192 | 20.83 |
|  | 0.350 | Tourism | Stable coffee | 81 | 192 | 42.19 |
|  | 0.350 | Tourism | Stable hybrid | 19 | 192 | 9.90 |
|  | 0.350 | Tourism | Stable tea | 18 | 192 | 9.38 |
|  | 0.350 | Tourism | Tea-rising | 34 | 192 | 17.71 |
|  | 0.400 | Commercial | Coffee-rising | 20 | 123 | 16.26 |
|  | 0.400 | Commercial | Stable coffee | 11 | 123 | 8.94 |
|  | 0.400 | Commercial | Stable hybrid | 5 | 123 | 4.07 |
|  | 0.400 | Commercial | Stable tea | 73 | 123 | 59.35 |
|  | 0.400 | Commercial | Tea-rising | 14 | 123 | 11.38 |
|  | 0.400 | Education | Coffee-rising | 24 | 127 | 18.90 |
|  | 0.400 | Education | Stable coffee | 31 | 127 | 24.41 |
|  | 0.400 | Education | Stable hybrid | 12 | 127 | 9.45 |
|  | 0.400 | Education | Stable tea | 39 | 127 | 30.71 |
|  | 0.400 | Education | Tea-rising | 21 | 127 | 16.54 |
|  | 0.400 | Mixed | Coffee-rising | 74 | 378 | 19.58 |
|  | 0.400 | Mixed | Stable coffee | 81 | 378 | 21.43 |
|  | 0.400 | Mixed | Stable hybrid | 47 | 378 | 12.43 |
|  | 0.400 | Mixed | Stable tea | 123 | 378 | 32.54 |
|  | 0.400 | Mixed | Tea-rising | 53 | 378 | 14.02 |
|  | 0.400 | Residential | Coffee-rising | 40 | 170 | 23.53 |
|  | 0.400 | Residential | Stable coffee | 35 | 170 | 20.59 |
|  | 0.400 | Residential | Stable hybrid | 8 | 170 | 4.71 |
|  | 0.400 | Residential | Stable tea | 60 | 170 | 35.29 |
|  | 0.400 | Residential | Tea-rising | 27 | 170 | 15.88 |
|  | 0.400 | Tourism | Coffee-rising | 32 | 157 | 20.38 |
|  | 0.400 | Tourism | Stable coffee | 70 | 157 | 44.59 |
|  | 0.400 | Tourism | Stable hybrid | 14 | 157 | 8.92 |
|  | 0.400 | Tourism | Stable tea | 11 | 157 | 7.01 |
|  | 0.400 | Tourism | Tea-rising | 30 | 157 | 19.11 |
|  | 0.450 | Commercial | Coffee-rising | 12 | 83 | 14.46 |
|  | 0.450 | Commercial | Stable coffee | 8 | 83 | 9.64 |
|  | 0.450 | Commercial | Stable hybrid | 4 | 83 | 4.82 |
|  | 0.450 | Commercial | Stable tea | 50 | 83 | 60.24 |
|  | 0.450 | Commercial | Tea-rising | 9 | 83 | 10.84 |
|  | 0.450 | Education | Coffee-rising | 14 | 79 | 17.72 |
|  | 0.450 | Education | Stable coffee | 19 | 79 | 24.05 |
|  | 0.450 | Education | Stable hybrid | 7 | 79 | 8.86 |
|  | 0.450 | Education | Stable tea | 28 | 79 | 35.44 |
|  | 0.450 | Education | Tea-rising | 11 | 79 | 13.92 |
|  | 0.450 | Mixed | Coffee-rising | 113 | 547 | 20.66 |
|  | 0.450 | Mixed | Stable coffee | 111 | 547 | 20.29 |
|  | 0.450 | Mixed | Stable hybrid | 59 | 547 | 10.79 |
|  | 0.450 | Mixed | Stable tea | 183 | 547 | 33.46 |
|  | 0.450 | Mixed | Tea-rising | 81 | 547 | 14.81 |
|  | 0.450 | Residential | Coffee-rising | 26 | 119 | 21.85 |
|  | 0.450 | Residential | Stable coffee | 31 | 119 | 26.05 |
|  | 0.450 | Residential | Stable hybrid | 3 | 119 | 2.52 |
|  | 0.450 | Residential | Stable tea | 38 | 119 | 31.93 |
|  | 0.450 | Residential | Tea-rising | 21 | 119 | 17.65 |
|  | 0.450 | Tourism | Coffee-rising | 25 | 127 | 19.69 |
|  | 0.450 | Tourism | Stable coffee | 59 | 127 | 46.46 |
|  | 0.450 | Tourism | Stable hybrid | 13 | 127 | 10.24 |
|  | 0.450 | Tourism | Stable tea | 7 | 127 | 5.51 |
|  | 0.450 | Tourism | Tea-rising | 23 | 127 | 18.11 |

Table S15e. Association tests under alternative FICI thresholds.

| **City** | **FICI threshold** | **Association tested** | **Sample size (n)** | **Chi-square statistic** | **Degrees of freedom** | **p-value** | **Cramér's V** |
| --- | --- | --- | --- | --- | --- | --- | --- |
| Jinan | 0.350 | Functional zone × cultural zone | 455 | 27.797 | 8 | <0.001 | 0.175 |
| Jinan | 0.350 | Functional zone × trajectory type | 455 | 41.643 | 16 | <0.001 | 0.151 |
| Jinan | 0.400 | Functional zone × cultural zone | 455 | 32.857 | 8 | <0.001 | 0.190 |
| Jinan | 0.400 | Functional zone × trajectory type | 455 | 37.499 | 16 | 0.002 | 0.144 |
| Jinan | 0.450 | Functional zone × cultural zone | 455 | 21.248 | 8 | 0.007 | 0.153 |
| Jinan | 0.450 | Functional zone × trajectory type | 455 | 31.567 | 16 | 0.011 | 0.132 |
| Qingdao | 0.350 | Functional zone × cultural zone | 955 | 114.215 | 8 | <0.001 | 0.245 |
| Qingdao | 0.350 | Functional zone × trajectory type | 955 | 121.867 | 16 | <0.001 | 0.179 |
| Qingdao | 0.400 | Functional zone × cultural zone | 955 | 108.707 | 8 | <0.001 | 0.239 |
| Qingdao | 0.400 | Functional zone × trajectory type | 955 | 118.565 | 16 | <0.001 | 0.176 |
| Qingdao | 0.450 | Functional zone × cultural zone | 955 | 95.723 | 8 | <0.001 | 0.224 |
| Qingdao | 0.450 | Functional zone × trajectory type | 955 | 99.952 | 16 | <0.001 | 0.162 |

Note. FICI refers to the functional-zone dominant-share threshold used to assign grid-level functional-zone labels. The main specification uses 0.40; alternative thresholds of 0.35 and 0.45 were used to evaluate threshold sensitivity. Percentages are calculated within the relevant functional-zone or comparable-grid sample.

# **S5.6 Summary of robustness findings**

Overall, the sensitivity and robustness analyses support the main conclusions of the study, while also clarifying the appropriate scope of interpretation for different indicators. Across alternative study-area masks, the main city-level patterns remained stable. The comparison between the main analytical mask, the expanded active mask, and the one-ring expanded mask showed that the overall differences between Jinan and Qingdao were not driven by a single mask definition. Although the inclusion of low-activity or peripheral grids changed the number of valid grids and affected some proportional indicators, the main contrast between the two cities remained consistent.

The robustness checks for CR thresholds and trajectory classification also supported the stability of the main interpretation. Alternative CR cutoffs produced limited changes in the broad classification of tea-dominant, hybrid, and coffee-dominant grids. More importantly, the direction of the city comparison was not reversed under alternative threshold settings. Trajectory classifications also remained highly stable, with at least 97.20% of grids retaining the same trajectory type under the tested threshold alternatives.

The annual dynamics and short-term fluctuation checks further confirmed that the endpoint-based CTI results should be interpreted together with year-by-year changes. These checks support the use of CTI as a concise summary of endpoint change, while also showing why CTI should not be interpreted as a complete substitute for annual dynamics. The additional response-evidence files were retained in the revised reproducibility package to document annual dynamics, adjacent-year cultural-zone transitions, short-term reversals, and endpoint-window sensitivity.

The spatial autocorrelation checks strengthened the interpretation of long-run spatial structure. Period-level CR and HI showed significant positive spatial autocorrelation in both cities, indicating that long-run coffee–tea composition and hybridization were spatially structured rather than randomly distributed. However, CTI showed weaker and more city-specific spatial autocorrelation: it was small but significant in Jinan and not significant in Qingdao. This result does not weaken the main conclusions; rather, it clarifies that long-run cultural structure and endpoint transition intensity capture different aspects of coffee–tea cultural change.

The FICI threshold-sensitivity analysis further showed that exact functional-zone labels were moderately sensitive to the dominant-share threshold, mainly through shifts into or out of the Mixed category. Classification stability ranged from 77.36% to 82.20% across alternative thresholds. However, the associations between functional zones and cultural zones or trajectory types remained statistically significant under all tested thresholds. Therefore, the functional-zone analysis should be interpreted as association-robust rather than label-invariant.

Taken together, these robustness checks show that the main findings are not dependent on a single study-area mask, a single CR threshold scheme, a single functional-zone threshold, or a purely endpoint-based interpretation of temporal change. The core conclusions remain stable: Jinan and Qingdao exhibited different long-run coffee–tea cultural compositions, different degrees of hybridization, and different spatial-functional expressions of coffee–tea cultural space. At the same time, the supplementary checks support a cautious interpretation of CTI and functional-zone associations. CTI is best understood as a descriptive measure of directional change from 2018 to 2024, while functional zones should be interpreted as contextual spatial settings rather than causal determinants of cultural transition.
