# Supplementary material for: Comparative dynamics of coffee–tea cultural spaces in two Chinese cities: Evidence from Qingdao and Jinan, 2018–2024
Source: PLoS One. 2026 Aug 3;21(8):e0355398. doi: 10.1371/journal.pone.0355398 (PMC13432132; doi:10.1371/journal.pone.0355398)
Supplement: Attachment — Submitted filename: Response to Reviewers20260606.docx [file pone.0355398.s026.docx]

# Response to Reviewers

# Title: Comparative dynamics of coffee–tea cultural spaces in two Chinese cities: Evidence from Qingdao and Jinan, 2018–2024

**Dear Editors and Reviewers,**

We sincerely appreciate your valuable time and insightful comments on our manuscript. We have carefully considered all the suggestions and made comprehensive revisions to the manuscript. Our responses are provided point by point below.

# Part 1. Journal Requirements

## 【Q1】 Response to Comment on PLOS ONE Style Requirements and File Naming

**Response:**

Thank you for this reminder. We have carefully checked the revised submission files against the PLOS ONE style and formatting requirements. The main manuscript has been revised and prepared as both a clean version and a marked-up version with tracked changes. We also checked the organization of the title page, author information, main text, tables, figures, supporting information legends, and file naming to ensure consistency with the journal requirements.

**Revision made:**

We prepared and renamed the revised submission files according to the PLOS ONE revision requirements. The revised manuscript is provided as a clean copy, and a separate marked-up copy is provided to show the changes made during revision. The main manuscript includes the main table within the manuscript file, while supporting information files are prepared separately.

**Location:**

Revised submission files; main manuscript; marked-up manuscript; supporting information files.

## 【Q2】Response to Comment on Copyrighted Map/Satellite Images in Figures

**Comment:**

We note that the map-based figures in your submission contain map/satellite images which may be copyrighted. Please either provide written permission from the copyright holder to publish these images under the CC BY 4.0 license, or remove these images from the submission.

**Response:**

Thank you for pointing this out. We understand the copyright concern regarding map or satellite images in the submitted figures. During revision, we added a new research-framework diagram as Fig. 1, which changed the numbering of the subsequent figures. The figures corresponding to the previously flagged map-based figures are now Fig. 2, Fig. 4, Fig. 5, and Fig. S1 in the revised manuscript and Supporting Information.

**Revision made:**

We chose the second option suggested by the journal and removed any potentially copyrighted map or satellite basemap images from the revised submission. The relevant figures were regenerated using author-produced vector/grid-based analytical outputs rather than third-party map or satellite imagery. The revised Fig. 2, Fig. 4, Fig. 5, and Fig. S1 show the study-area masks, analytical grids, and POI-derived spatial patterns without using copyrighted map or satellite basemaps.

**Location:**

Revised Fig. 2, Fig. 4, Fig. 5, and Fig. S1; revised figure files uploaded with the revised submission.

**Fig. 2. Final grid-based study-area masks for Jinan and Qingdao.**

**Fig. 4. Long-run spatial structure and cultural mixing of coffee–tea spaces in Jinan and Qingdao.**

**Fig. 5. Spatial patterns of coffee–tea transition direction, CTI classes, and long-term trajectory composition in Jinan and Qingdao, 2018–2024.**

**Fig. S1. Spatial distribution and activity-volume-stratified patterns of the hybridization index (HI).**

## 【Q3】Response to Comment on Supplementary Figure File Type and Supporting Information Legends

**Comment:**

We notice that your supplementary figures are uploaded with the file type “Figure”. Please amend the file type to “Supporting Information”. Please ensure that each Supporting Information file has a legend listed in the manuscript after the references list.

**Response:**

Thank you for this reminder. We have corrected the submission-file organization for the supplementary figures. In the revised submission, the supplementary figures will be uploaded as “Supporting Information” files rather than as main “Figure” files.

**Revision made:**

We checked the Supporting Information section and added the legends for all supplementary figures after the reference list in the revised manuscript. The legends for Fig. S1, Fig. S2, Fig. S3, and Fig. S4 are now listed in the Supporting Information section of the manuscript.

**Location:**

Supporting Information section after the References; Fig. S1–Fig. S4 legends; revised Supporting Information files uploaded with the revised submission.

## 【Q4】Response to Comment on Referring to Figure 1 in the Text

**Comment:**

Please ensure that you refer to Figure 1 in your text as, if accepted, production will need this reference to link the reader to the figure.

**Response:**

Thank you for this reminder. During revision, we added a new research-framework diagram as Fig. 1, so the figure referred to in this comment now corresponds to Fig. 2 in the revised manuscript. We have checked the revised manuscript and ensured that the relevant figure is explicitly cited in the main text.

**Revision made:**

We added an explicit reference to Fig. 2 in Methods Section 2.3, where the fixed grid framework and city-specific study-area masks are described. The revised text now states that the resulting final grid-based study-area masks for Jinan and Qingdao are shown in Fig. 2. We also ensured that the newly added Fig. 1 research-framework diagram is cited in Methods Section 2.1.

**Location:**

Methods, Section 2.3 “Fixed grid framework and construction of city-specific study-area masks”, lines 128–129; Fig. 2. The newly added research-framework diagram is also cited in Methods, Section 2.1; Fig. 1.

## 【Q5】Response to Comment on Referring to Table 1 in the Text

**Comment:**

We note you have included a table to which you do not refer in the text of your manuscript. Please ensure that you refer to Table 1 in your text; if accepted, production will need this reference to link the reader to the Table.

**Response:**

Thank you for this reminder. We have checked the revised manuscript and ensured that Table 1 is explicitly cited in the main text.

**Revision made:**

We added an explicit reference to Table 1 in Methods Section 2.1, where the comparative study design and analytical framework are introduced. Table 1 summarizes the key indicators and analytical concepts used in the study, including POI-observed coffee–tea cultural spaces, Coffee Ratio (CR), Hybridization Index (HI), Cultural Transition Intensity (CTI), cultural zones, trajectory types, and functional zones.

**Location:**

Methods, Section 2.1 “Comparative study design and analytical framework”, lines 97–98; Table 1.

## 【Q6】Response to Comment on Including Main Tables in the Manuscript

**Comment:**

Please include your tables as part of your main manuscript and remove the individual files. Please note that supplementary tables should remain uploaded as separate “Supporting Information” files.

**Response:**

Thank you for this reminder. We have revised the submission-file organization accordingly. The main table is now included directly in the main manuscript file, rather than being uploaded as an individual table file.

**Revision made:**

Table 1 has been embedded in the main manuscript as part of the revised manuscript file. The supplementary tables are retained as separate Supporting Information files, as requested by the journal.

**Location:**

Main manuscript, Table 1, lines 100–101; Supporting Information table files.

## 【Q7】Response to Comment on Reviewer-Recommended References

**Comment:**

If the reviewer comments include a recommendation to cite specific previously published works, please review and evaluate these publications to determine whether they are relevant and should be cited. There is no requirement to cite these works unless the editor has indicated otherwise.

**Response:**

Thank you for this guidance. We understand that there is no requirement to cite specific works unless they are directly relevant to the manuscript or specifically required by the editor. We reviewed the reviewer comments and evaluated whether any suggested or thematically relevant publications were necessary for strengthening the revised manuscript.

**Revision made:**

Where additional literature was directly relevant to the study background, methodological framing, or interpretation of the findings, we incorporated appropriate references. We did not add references solely for citation purposes, and no citation was added unless it was relevant to the revised analysis and discussion.

**Location:**

Introduction; Methods, Section 2.7; Discussion; References.

# Part 2. Response to Editor Comments

## 【Q1】POI counts reflect venue presence, not lived cultural practice. Claims about “cultural transformation” should clarify this limitation.

**Response:**

We thank the editor for this important comment. We fully agree that POI counts reflect the observed presence and spatial composition of venues rather than lived cultural practices, cultural meanings, interaction intensity, or actual consumer behavior. To address this issue, we revised the manuscript to clarify the interpretive boundary of the study and to avoid overextending POI-derived results into claims about lived cultural practice.

**Revision made:**

First, we revised the Abstract to define the analytical object more cautiously. The revised Abstract now states that the study uses CR, HI, CTI, long-run cultural zones, temporal trajectory types, functional-zone context, and supplementary robustness checks “to examine POI-observed coffee–tea venue patterns.” The concluding sentence of the Abstract further states that “POI-observed coffee–tea venue restructuring is better understood as differentiated reorganization characterized by coexistence, selective concentration, and uneven directional change rather than as a single linear process of substitution.” These changes replace broader claims about cultural transformation with a more bounded focus on POI-observed venue patterns.

Second, we revised the Discussion to use bounded terminology when interpreting the results. For example, Section 4.1 now states that “the present study does not support understanding changes in POI-observed coffee–tea venue patterns as a simple linear substitution between coffee and tea.” This wording clarifies that the study concerns observed venue patterns, not lived cultural practice itself.

Third, we added an explicit limitation statement in Section 4.5. The revised manuscript now states: “this study is based on platform-derived POI records and should therefore be interpreted as evidence of observed venue presence and spatial composition rather than as a complete representation of urban cultural practice itself.” The same paragraph further clarifies that “the results should therefore be understood as evidence of POI-observed cultural-space organization, not as a direct measure of lived cultural practice.” This directly addresses the editor’s concern about the interpretive limits of POI counts.

Finally, we revised the Conclusion to restate this boundary. The final paragraph now states that the findings should be interpreted “within the boundaries of POI-observed venue presence and spatial composition rather than as a complete representation of lived cultural practice or a causal account of underlying drivers.”

Together, these revisions clarify that the study provides evidence on POI-observed venue presence, spatial composition, coexistence, and restructuring, while avoiding claims that POI counts directly measure lived cultural practice.

**Location:**

Abstract, lines 12–30;

Discussion, Section 4.1 “Beyond replacement: differentiated restructuring of POI-observed coffee–tea venue patterns”, lines 389–391;

Limitations, Section 4.5, first paragraph, lines 520–526;

Conclusion, final paragraph, lines 561–564.

## 【Q2】Provide full details on POI cleaning, category harmonization, and validation; include duplicates removed, thresholds, and manual checks

**Response:**

Thank you for this important suggestion. We agree that the POI cleaning, category harmonization, and validation procedures should be described more transparently, because the reliability of the grid-based analysis depends on how platform-derived POI records were classified, cleaned, and linked across years. To address this concern, we expanded the Methods section, substantially revised S1 Appendix, added cleaning-log summaries and anonymized POI sample records to the public reproducibility package, and updated the Data Availability Statement.

**Revision made:**

First, we clarified the roles of the two POI sources in the revised Methods and S1 Appendix. The revised manuscript now states that “Dianping POIs were used to identify coffee-shop and teahouse venues, whereas Amap (Gaode Map) POIs were used to construct the grid-level functional-zone context.” We also clarified that “the two sources were not treated as equivalent POI systems for direct comparison; rather, they served separate analytical roles within the same 500 m × 500 m grid framework.” This distinction was added to prevent confusion between the POI source used for coffee–tea venue identification and the POI source used for functional-zone context.

Second, we added detailed ID-based cleaning and duplicate-screening rules in S1 Appendix. Section S1.2.1 now states that “Dianping POI records were deduplicated using the stable platform venue identifier as the primary key rather than relying on establishment names alone.” It further specifies the distance thresholds used for duplicate and positional-jitter screening: “Records located within 50 m of one another and sharing the same Dianping venue ID were treated as likely positional jitter or near-duplicate entries and were flagged for consolidation review. Records located within 50–100 m were not automatically treated as duplicates but were flagged for targeted manual checking.” This directly responds to the request to report the thresholds used in duplicate screening.

Third, we added the manual-verification procedure for inconsistent records. Section S1.2.2 now states that “targeted manual checks were conducted for cases in which the same Dianping venue ID appeared with multiple addresses within the same year.” It further clarifies that these cases were reviewed to determine whether they represented the same establishment with relocation or renaming, or distinct establishments incorrectly sharing an identifier. Records with unresolved ID–address inconsistency were excluded from ID-based longitudinal linkage to reduce downstream measurement uncertainty. This revision clarifies the manual-check procedure and the exclusion rule for unresolved cases.

Fourth, we expanded the category-harmonization rules for coffee shops and teahouses. The revised Methods and S1 Appendix define coffee shops as venues “whose primary business identity was coffee consumption,” including coffee shops, cafés, coffee houses, and specialty coffee stores. Teahouses are defined as venues “whose primary business identity was on-site tea drinking, tea-room consumption, or traditional tea-related social consumption,” including tea houses, tea rooms, traditional teahouses, tea clubs, tea ceremony spaces, and comparable Chinese categories such as 茶馆, 茶楼, 茶室, and 茶社 when the venue primarily functioned as an on-site tea consumption space.

Fifth, we clarified the exclusion rules for ambiguous or non-target beverage venues. The revised Methods and S1 Appendix now state that “milk-tea shops, fruit-tea shops, general beverage shops, dessert drink shops, restaurants with incidental tea service, non-coffee-oriented cafés, and ambiguous mixed beverage venues were excluded unless their primary business identity clearly matched the operational definition of coffee shop or teahouse.” This directly addresses the need to distinguish teahouses from milk-tea shops, beverage shops, cafés, and restaurants with incidental tea service.

Sixth, we added the year-specific harmonization and validation procedure. Section S1.2.3 now explains that raw Dianping category labels were mapped to the target analytical classes using a fixed rule-based procedure: direct category mapping, cross-year reconciliation, keyword-assisted validation, and manual review of ambiguous cases. The S1 Appendix states that “for coffee and tea identification, category mapping was cross-checked using name or label keywords to reduce misclassification when platform categories were ambiguous,” and that “ambiguous cases were reviewed based on venue name, platform category label, and available descriptive information.” Records that could not be confidently assigned to either coffee shop or teahouse were excluded from the analytical coffee–tea dataset.

Seventh, we added reporting and reproducibility materials outside the main manuscript tables. The public reproducibility package includes POI category-harmonization documentation, cleaning-log summaries, and anonymized POI sample records. These materials report and document the preprocessing workflow, including category harmonization, duplicate screening, coordinate screening, and final cleaned analytical records, while avoiding redistribution of restricted raw shop-level POI data. The anonymized POI sample records exclude identifying information such as shop names, full addresses, platform IDs, precise coordinates, and geometries, while retaining non-identifying fields needed to inspect the cleaned data structure.

Together, these revisions provide the requested details on POI source roles, category harmonization, duplicate screening, threshold settings, manual checks, exclusion criteria, cleaning-log summaries, anonymized POI sample records, and reproducibility materials.

**Location:**

Methods, Section 2.2 “Study areas, POI data, and preprocessing”, lines 103–117;

S1 Appendix, Section S1.1 “POI data sources”, Section S1.2.1 “ID-based deduplication and proximity screening”, Section S1.2.2 “Targeted manual verification for inconsistent ID–address pairs”, and Section S1.2.3 “Year-specific category harmonization for coffee-shop and teahouse venues”;

Data Availability Statement, lines 567-580;

public Zenodo reproducibility package, <https://doi.org/10.5281/zenodo.20565270>.

## 【Q3】Study-area masks are constructed from POI counts; may bias results by excluding low-activity or emerging grids. Include sensitivity tests with alternative masks.

**Response:**

Thank you for this important suggestion. We agree that study-area masks derived from observed coffee–tea POI activity may potentially affect the results by excluding low-activity, peripheral, or newly emerging grids. To address this concern, we revised the manuscript in two ways. First, we clarified the construction rules for the main study-area masks. Second, we added a study-area mask sensitivity analysis using alternative mask definitions to test whether the main findings were sensitive to the mask delineation procedure.

**Revision made:**

First, we clarified the main mask-construction procedure in the Methods and S1 Appendix. The revised Methods now states that “a grid was retained as a core activity grid if it had a cumulative coffee + tea count of at least 4 during 2018–2024 and observed activity in at least 4 distinct years.” It further explains that core grids were expanded “by two rounds of queen adjacency,” grouped into contiguous clusters, refined by excluding small clusters, and filled where enclosed interior holes were 10 km² or less. S1 Appendix provides the stepwise mask-construction procedure, including activity presence, minimum cumulative activity intensity, temporal persistence, queen-adjacency expansion, cluster-level refinement, and enclosed-hole filling.

Second, we explicitly acknowledged the potential bias of a POI-derived mask and added alternative mask definitions. The revised Methods now states: “Because the main masks were derived from observed coffee–tea venue activity, alternative mask definitions were evaluated in the sensitivity analysis described in Section 2.8 and S5 Appendix.” In Section 2.8, we added the following sensitivity-test design: “we compared the main analytical mask with two alternative mask definitions. The expanded active mask included grids with coffee-shop or teahouse activity in any year during 2018–2024, whereas the one-ring expanded mask was constructed by expanding the main analytical mask outward by one Queen-contiguity grid ring.” This directly addresses the concern that the main mask might exclude low-frequency, peripheral, or emerging coffee–tea grids.

Third, we reported the mask-sensitivity results in the revised Results section and Supporting Information. Section 3.5 now states that “the study-area mask sensitivity analysis showed that the main differences between Jinan and Qingdao remained broadly consistent under alternative mask definitions, including the main analytical mask, the expanded active mask, and the one-ring expanded mask.” The detailed results are reported in Table S10, “Study-area mask sensitivity analysis.” Overall, the additional analysis shows that although some absolute composition estimates may vary under broader mask definitions, the main comparative conclusions remain stable.

**Location:**

Methods, Section 2.3 “Fixed grid framework and construction of city-specific study-area masks”, lines 123–131;

Methods, Section 2.8 “Sensitivity and supplementary robustness analyses”, lines 183–187;

Results, Section 3.5 “Robustness checks”, lines 370–372;

S1 Appendix, Section S1.4 “Construction of the study-area mask”;

S5 Appendix, Section S5.1 “Study-area mask sensitivity”;

Table S10. Study-area mask sensitivity analysis.

## 【Q4】Coffee Ratio (CR) thresholds (≤0.3, 0.3–0.7, ≥0.7) and trajectory classifications are arbitrary. Test robustness with alternative cutoffs

**Response:**

Thank you for this important comment. We agree that both the CR thresholds used for cultural-zone classification and the thresholds used in trajectory classification may involve a degree of arbitrariness. To address this concern, we revised the Methods to clarify the main classification rules and added sensitivity analyses using alternative CR cutoff settings.

**Revision made:**

First, we clarified the main CR-based cultural-zone classification in the Methods. The revised manuscript now states: “Based on period-aggregated CR, each grid was classified as tea-dominant (CR ≤ 0.30), hybrid (0.30 < CR < 0.70), or coffee-dominant (CR ≥ 0.70).” This makes the main threshold specification explicit.

Second, we clarified the trajectory-classification procedure. The revised manuscript now states that “a simple linear slope of annual CR against year was calculated using valid observed annual CR values.” It further specifies that “a grid was classified as coffee rising when the slope was greater than 0.05 and as tea rising when the slope was less than −0.05.” For grids within the stable slope band, the manuscript now states: “Grids with slopes within the stable band from −0.05 to 0.05 were then classified using mean annual CR. Within this stable band, grids with mean annual CR at or below 0.30 were classified as stable tea, grids with mean annual CR at or above 0.70 were classified as stable coffee, and the remaining grids were classified as stable hybrid.” These revisions clarify that trajectory classification uses both annual CR slope and mean annual CR, rather than relying only on endpoint states.

Third, we added robustness checks using alternative CR threshold settings. In the revised Methods, Section 2.8 now states: “because the classification of cultural zones and stable trajectory categories depends on CR thresholds, we tested alternative threshold pairs of 0.25/0.75 and 0.35/0.65 against the main 0.30/0.70 specification.” The purpose of these checks was to evaluate whether the cultural-zone composition and five-category trajectory classifications were robust to reasonable changes in threshold settings.

Fourth, we reported the robustness results in the revised Results section and Supporting Information. Section 3.5 now states that “the CR-threshold and trajectory-classification sensitivity analyses also showed that the relative contrast between Jinan’s stronger tea-dominant and mixed structure and Qingdao’s stronger coffee-dominant tendency remained similar under alternative cutoff settings.” The detailed results are reported in Table S11, “CR-threshold sensitivity analysis,” and Table S12, “Trajectory-classification sensitivity analysis.”

Together, these revisions directly address the concern about arbitrary CR thresholds and trajectory-classification rules. Although the absolute proportions of some categories may vary under alternative cutoffs, the main comparative interpretation remains stable: Jinan retains a stronger tea-dominant and mixed structure, whereas Qingdao retains a stronger coffee-dominant tendency.

**Location:**

Methods, Section 2.5 “Cultural indicators and spatial cultural zoning”, lines 153–154;

Methods, Section 2.6 “Cultural trajectory identification”, lines 161–165;

Methods, Section 2.8 “Sensitivity and supplementary robustness analyses”, lines 188–191;

Results, Section 3.5 “Robustness checks”, lines 372–375;

S5 Appendix, Sections S5.2–S5.3;

Table S11 “CR-threshold sensitivity analysis”; Table S12 “Trajectory-classification sensitivity analysis.”

## 【Q5】CTI measures endpoint change only (2018→2024); annual dynamics, COVID-era fluctuations, and short-term reversals should be shown

**Response:**

Thank you for this important comment. We agree that CTI is an endpoint-based indicator and, by itself, does not describe the full annual pathway of coffee–tea venue change. In the original version, the CTI results may have given the impression that the 2018→2024 endpoint difference fully represented the temporal process. To address this concern, we revised the manuscript to clarify the scope of CTI and added supplementary annual-dynamics, short-term fluctuation, and endpoint-sensitivity checks.

**Revision made:**

First, we clarified the definition and interpretation of CTI in the Methods. The revised manuscript now states that, “for endpoint-based change analysis, signed endpoint change was first calculated as ΔCR = CR2024 − CR2018 for grids with valid annual CR values in both endpoint years.” It further clarifies that “positive ΔCR indicates coffee-ward change, whereas negative ΔCR indicates tea-ward change,” and that “CTI was then defined as |ΔCR| and used to represent the magnitude of endpoint-based net change.” This revision makes clear that CTI measures endpoint-based net change rather than continuous annual dynamics.

Second, we added supplementary checks to show annual dynamics and short-term fluctuations. In Section 2.8, the revised manuscript now states that “because CTI is an endpoint-based magnitude indicator derived from signed endpoint change, we supplemented the main 2018–2024 endpoint comparison with annual dynamics, adjacent-year cultural-zone transition matrices, short-term reversal checks, and alternative endpoint-window diagnostics.” The same paragraph clarifies that these analyses were used “to clarify whether endpoint-based CTI patterns were consistent with broader year-by-year temporal evidence.”

Third, we added endpoint-sensitivity evidence in the Results and Supporting Information. The revised Results section now states that “the supplementary endpoint-sensitivity analysis showed broad robustness of the main signed endpoint-change patterns.” Instead of using only the 2018→2024 endpoint comparison, we compared the main endpoint definition with alternative 2018→2022 and 2018→2023 endpoint comparisons. The revised Results report that, using 2023 as the alternative endpoint, Pearson correlations between signed endpoint changes for 2018→2024 and 2018→2023 were 0.947 in Jinan and 0.891 in Qingdao, with sign agreement of 90.2% and 86.7%, respectively. Using 2022 as the alternative endpoint, the corresponding correlations were 0.842 and 0.757, with sign agreement of 78.0% and 78.6%. The manuscript also reports that direction-reversal rates ranged from 3.5% to 7.8%, and that hotspot overlap was higher for 2023 than for 2022.

Fourth, we added the corresponding supplementary outputs. Fig. S3 presents the endpoint-sensitivity analysis of signed coffee–tea endpoint change, and Table S9 reports the sensitivity summary for alternative CTI endpoint definitions. Additional annual-dynamics, adjacent-year transition, short-term reversal, and endpoint-window response-evidence files are included in the public reproducibility package.

**Fig. S3. Endpoint-sensitivity analysis of signed coffee–tea endpoint change.**

Together, these revisions address the concern that CTI is an endpoint-only measure. The revised manuscript now treats CTI as a bounded endpoint-change indicator and uses supplementary annual and endpoint-sensitivity evidence to show whether the endpoint-based patterns are consistent with broader year-by-year dynamics.

**Location:**

Methods, Section 2.5 “Cultural indicators and spatial cultural zoning”, lines 150–154;

Methods, Section 2.8 “Sensitivity and supplementary robustness analyses”, lines 192–195;

Results, Section 3.3 “Spatial transition intensity and long-term cultural trajectories”, lines 303–308;

Results, Section 3.5 “Robustness checks”, lines 369–382;

S5 Appendix, Section S5.4;

public Zenodo reproducibility package, <https://doi.org/10.5281/zenodo.20565270>;

Fig. S3; Table S9.

## 【Q6】Functional zones derived from POI composition may be endogenous; address spatial autocorrelation or use external land-use data.

**Response:**

Thank you for this important comment. We agree that functional zones derived from POI composition should not be interpreted as exogenous causal variables, especially because both the functional-zone context and the coffee–tea venue indicators are based on POI-derived urban activity data. To address this concern, we revised the manuscript to clarify the interpretive role of functional zones, reduced causal wording, excluded coffee/tea-related categories from the functional-zone classification, added spatial autocorrelation checks, and added functional-zone threshold sensitivity analyses.

**Revision made:**

First, we clarified that functional zones are used as contextual descriptors rather than causal explanatory variables. In the revised Methods, Section 2.7 now states: “In this study, functional zones were used as contextual descriptors rather than causal explanatory variables.” This revision directly limits the interpretation of functional-zone results and makes clear that the analysis describes spatial-functional associations rather than causal effects.

Second, we reduced potential mechanical overlap between the coffee–tea indicators and the functional-zone classification. The revised Methods now states that “coffee-shop, teahouse, milk-tea, fruit-tea, general beverage, and dessert drink categories were excluded from the Amap-based functional-zone classification to reduce mechanical overlap with the Dianping-based coffee–tea indicators.” This exclusion rule was added to reduce the possibility that coffee–tea venues directly determined the functional-zone labels used in the contextual analysis.

Third, we added spatial autocorrelation checks. In Section 2.8, the revised manuscript now states that “because grid-level observations may be spatially structured, we conducted global Moran’s I tests for the main cultural indicators using Queen contiguity weights.” The same paragraph clarifies that these tests were used “to assess spatial autocorrelation in annual CR and HI, period-level CR and HI, and endpoint CTI.” The results are reported in Table S14. In the revised Results, Section 3.5 further states that “spatial autocorrelation checks showed that several indicators had significant spatial structure, supporting the need to interpret the results as spatially structured descriptive patterns rather than independent grid-level observations.”

Fourth, we added functional-zone threshold sensitivity checks. Section 2.8 now states that “because functional-zone classification depends on the dominant-share threshold, we tested alternative functional-zone thresholds of 0.35 and 0.45 against the main 0.40 specification.” The results are reported in Table S15. In Section 3.5, we summarize that “functional-zone threshold sensitivity checks further showed that the main functional-embedding patterns remained consistent, although the size of the Mixed category changed with the dominant-share threshold.”

Fifth, we revised the Discussion and Limitations to avoid causal interpretation. In Section 4.3, the revised manuscript now states that the functional-zone correspondence “should not be interpreted as evidence of a single deterministic land-use mechanism.” It further explains that functional zones were used as contextual descriptors and that the results should be interpreted as associations between functional settings and observed coffee–tea venue patterns. In Section 4.5, we also added that “because functional zones are empirically derived from POI composition and adjacent grids may exhibit spatial autocorrelation, the observed associations should be interpreted as contextual and descriptive rather than as strictly independent causal effects.”

Together, these revisions address the reviewer’s concern by clarifying the contextual role of POI-derived functional zones, reducing mechanical overlap in the functional-zone classification, testing spatial autocorrelation, and evaluating whether functional-zone associations remain stable under alternative threshold settings. We did not introduce external land-use data in this revision because the purpose of the added analyses was to strengthen the internal robustness and interpretive caution of the existing POI-based framework.

**Location:**

Methods, Section 2.7 “Functional zoning and contextual analyses”, lines 169–174;

Methods, Section 2.8 “Sensitivity and supplementary robustness analyses”, lines 196–203;

Results, Section 3.5 “Robustness checks”, lines 377–382;

Discussion, Section 4.3 “Functional embedding as contextual association”, lines 493–498;

Limitations, Section 4.5, lines 537–540;

S4 Appendix; S5 Appendix, Sections S5.5–S5.6;

Table S14. Spatial autocorrelation results;

Table S15. Functional-zone threshold sensitivity analysis for functional-zone associations with cultural zones and trajectory types.

## 【Q7】Only two cities analyzed; claims about broader Chinese urban transformation must be softened.

**Response:**

Thank you for this important comment. We agree that the study is based on two cities only and therefore should not make broad claims about Chinese urban transformation as a whole. To address this concern, we revised the manuscript to soften general claims and to frame the results more explicitly as comparative evidence from two contrasting urban cases, Jinan and Qingdao. We also clarified in the Limitations section that the findings should not be interpreted as a complete typology of all Chinese cities.

**Revision made:**

First, we revised the Abstract to frame the study as a two-city comparison rather than as a general account of Chinese urban transformation. The revised Abstract now states that “this study compares Jinan and Qingdao, two major cities in Shandong Province with different historical and functional settings.” The conclusion of the Abstract also refers specifically to “POI-observed coffee–tea venue restructuring” rather than broader Chinese urban cultural transformation.

Second, we revised the Introduction to define the empirical scope more clearly. The revised manuscript now states: “Against this background, this study compares Qingdao and Jinan as two contrasting urban cases.” This wording clarifies that the study is designed as a comparative case analysis rather than a nationally representative study.

Third, we softened the Discussion by emphasizing city-specific pathways and place-specific interpretation. For example, Section 4.2 now states that “the two cities did not evolve along a single pathway of coffee–tea venue change, but rather exhibited different modes of restructuring under a shared analytical design.” Section 4.4 further states that “urban locality should be treated as an important context for interpreting POI-observed venue patterns, rather than merely as a background condition.” These revisions shift the interpretation from broad generalization to place-specific comparative evidence.

Fourth, we added a direct limitation statement in Section 4.5. The revised manuscript now states: “although the unified framework improves comparability, the analysis is based on two cities only, and the findings should therefore be read as comparative evidence rather than as a complete typology of all Chinese cities.” This explicitly addresses the reviewer’s concern about external validity and generalization.

Finally, we revised the Conclusion to avoid overgeneralization. The final sentence now states that the study provides “comparative empirical evidence for understanding how emerging and established everyday consumption venues are reorganized, sustained, and unevenly embedded in different urban contexts.” This wording presents the findings as comparative evidence from two urban contexts rather than as a broad claim about all Chinese cities.

Together, these revisions clarify that the manuscript does not claim to represent the full diversity of Chinese urban transformation. Instead, it uses Jinan and Qingdao as two contrasting cases to provide comparative evidence on POI-observed coffee–tea venue restructuring.

**Location:**

Abstract, lines 13–32;

Introduction, final paragraph, line 74;

Discussion, Section 4.2, lines 429–431;

Discussion, Section 4.4, lines 508–509;

Limitations, Section 4.5, lines 540–542;

Conclusion, final paragraph, lines 563–565.

## 【Q8】Improve reproducibility: share harmonization tables, cleaning logs, code, and anonymized POI samples

Response:

Thank you for this important suggestion. We agree that the reproducibility of the POI-based workflow should be strengthened, particularly because the analysis depends on POI category harmonization, record cleaning, spatial aggregation, and derived grid-level indicators. To address this concern, we revised the manuscript, expanded S1 Appendix, updated the Data Availability Statement, and deposited an updated public reproducibility package on Zenodo.

Revision made:

First, we expanded the documentation of POI data sources, category harmonization, and cleaning procedures in S1 Appendix. The revised appendix clarifies that “Dianping POIs were used to identify coffee-shop and teahouse venues, whereas Amap (Gaode Map) POIs were used to construct grid-level functional-zone context.” It also explains that the two sources “were not treated as equivalent POI systems for direct comparison,” but instead served separate analytical roles within the same 500 m × 500 m grid framework.

Second, we documented the coffee-shop and teahouse classification rules in detail. In S1 Appendix, coffee shops are defined as venues “whose primary business identity was coffee consumption,” including coffee shops, cafés, coffee houses, and specialty coffee stores. Teahouses are defined as venues “whose primary business identity was on-site tea drinking, tea-room consumption, or traditional tea-related social consumption,” including tea houses, tea rooms, traditional teahouses, tea clubs, tea ceremony spaces, and comparable Chinese categories such as 茶馆, 茶楼, 茶室, and 茶社 when the venue primarily functioned as an on-site tea consumption space. We also clarified the exclusion rules: “milk-tea shops, fruit-tea shops, general beverage shops, dessert drink shops, restaurants with incidental tea service, non-coffee-oriented cafés, and ambiguous mixed beverage venues were excluded unless their primary business identity clearly matched the operational definition of coffee shop or teahouse.”

Third, we added more detailed information on data cleaning and validation. S1 Appendix now states that Dianping POI records were deduplicated using the stable platform venue identifier as the primary key. Records located within 50 m of one another and sharing the same Dianping venue ID were treated as likely positional jitter or near-duplicate entries and were flagged for consolidation review, whereas records located within 50–100 m were flagged for targeted manual checking. The appendix also states that targeted manual checks were conducted for cases in which the same Dianping venue ID appeared with multiple addresses within the same year, and that records with unresolved ID–address inconsistency were excluded from ID-based longitudinal linkage.

Fourth, we documented the year-specific harmonization procedure. S1 Appendix now explains that raw Dianping category labels were mapped to the target analytical classes through a fixed rule-based procedure, including direct category mapping, cross-year reconciliation, keyword-assisted validation, and manual review of ambiguous cases. Ambiguous records that could not be confidently assigned to either coffee shop or teahouse were excluded from the analytical coffee–tea dataset.

Fifth, we updated the public reproducibility package. The revised public package has been deposited on Zenodo at <https://doi.org/10.5281/zenodo.20565270>. The package includes aggregated 500 m grid-level public analytical datasets, original main-analysis notebooks, newly added revision robustness-analysis notebooks, utility code, original output files, formal revision tables, response-evidence files, POI category-harmonization documentation, cleaning-log summaries, and anonymized POI sample records. The anonymized sample records exclude raw identifying information such as shop names, full addresses, platform IDs, precise coordinates, and geometries, while retaining non-identifying fields needed to inspect the cleaned data structure.

Sixth, we revised the Data Availability Statement to clarify what is publicly shared and what cannot be redistributed. The revised statement now says that the package includes “POI category-harmonization documentation, cleaning-log summaries, and anonymized POI sample records to support reproducibility of the data preprocessing workflow.” It also clarifies that raw shop-level POI records are not redistributed because they were obtained from third-party platforms and are subject to licensing and redistribution restrictions. This restriction applies only to the raw shop-level records; the aggregated analytical datasets and derived data necessary to reproduce the results reported in the manuscript are provided in the public reproducibility package.

Finally, we added the revision-analysis materials needed to reproduce the robustness checks. The updated reproducibility package includes notebooks and outputs for Tables S10–S15, covering study-area mask sensitivity, CR-threshold sensitivity, trajectory-classification sensitivity, CTI annual dynamics and short-term fluctuation checks, spatial autocorrelation checks, and functional-zone threshold sensitivity analyses.

Together, these revisions improve reproducibility by providing documented category-harmonization rules, cleaning and validation procedures, cleaning-log summaries, anonymized POI sample records, public grid-level analytical datasets, code, formal output tables, and response-evidence files, while respecting licensing restrictions on the complete raw shop-level POI data.

Location:

S1 Appendix, Sections S1.1–S1.2.3;

Data Availability Statement;

public Zenodo reproducibility package: <https://doi.org/10.5281/zenodo.20565270>;

revision outputs for Tables S10–S15.

## 【Q9】Abstract is dense; emphasize main findings and simplify methodology.

**Comment:**

Abstract is dense; emphasize main findings and simplify methodology.

**Response:**

Thank you for this helpful suggestion. We agree that the original Abstract contained too much methodological detail and that the main comparative findings needed to be presented more clearly. We therefore revised the Abstract to make it more concise, results-oriented, and consistent with the interpretive scope of the study.

**Revision made:**

First, we shortened the methodological description by retaining only the core analytical elements. The revised Abstract now states that “Coffee Ratio (CR), Hybridization Index (HI), and Cultural Transition Intensity (CTI) were combined with long-run cultural zones, temporal trajectory types, functional-zone context, and supplementary robustness checks to examine POI-observed coffee–tea venue patterns.” This replaces the longer previous description of multiple analytical dimensions.

Second, we reorganized the Abstract to emphasize the main findings more directly. The revised Abstract now highlights the key city-level contrast: “Jinan showed substantial overall expansion in coffee-shop and teahouse POIs, with total counts increasing by 64.6% from 2018 to 2024, while coffee share changed only slightly from 42.6% to 43.1%.” It then contrasts this with Qingdao, where “total counts” changed only slightly, but “coffee share” increased from 49.3% to 59.1%.

Third, we summarized the grid-level and functional-zone findings in a more compact form. The revised Abstract states that Qingdao had “clearer coffee-oriented restructuring, a higher coffee-dominant share than Jinan, and more stable coffee trajectories,” whereas Jinan retained “a stronger tea-oriented base, higher coexistence balance, and more bidirectional adjustment.” It also summarizes the functional-zone results by noting clearer functional differentiation in Qingdao and stronger links between mixed-use settings and cultural hybridity in Jinan.

Finally, we revised the concluding sentence to provide a clearer takeaway while avoiding overly broad claims. The revised Abstract now concludes that “POI-observed coffee–tea venue restructuring is better understood as differentiated reorganization characterized by coexistence, selective concentration, and uneven directional change rather than as a single linear process of substitution.”

Together, these revisions make the Abstract shorter, clearer, and more focused on the main comparative findings, while retaining the essential methodological information needed to understand the study.

**Location:**

Abstract, lines 13–32.

## 【Q10】Avoid broad “cultural transformation” claims; use POI-observed venue patterns

**Comment:**

Avoid broad “cultural transformation” claims; use POI-observed venue patterns.

**Response:**

Thank you for this important comment. We agree that broad claims about “cultural transformation” should be avoided because the present study is based on platform-derived POI records rather than direct observations of cultural practices, meanings, or social interactions. To address this concern, we revised the manuscript throughout to use more cautious and data-consistent terminology, especially “POI-observed coffee–tea venue patterns” and “POI-observed coffee–tea venue restructuring.”

**Revision made:**

First, we revised the Abstract to define the analytical object more precisely. The revised Abstract now states that CR, HI, CTI, long-run cultural zones, temporal trajectory types, functional-zone context, and supplementary robustness checks were used “to examine POI-observed coffee–tea venue patterns.” This wording replaces broader claims about cultural transformation with a direct reference to the type of evidence that the POI data can support.

Second, we revised the Abstract’s concluding statement. Instead of making a broad claim about “consumption-cultural transformation in contemporary Chinese cities,” the revised Abstract now states that “POI-observed coffee–tea venue restructuring is better understood as differentiated reorganization characterized by coexistence, selective concentration, and uneven directional change rather than as a single linear process of substitution.” This revision presents the conclusion as evidence about observed venue restructuring rather than as a direct claim about wider cultural transformation.

Third, we revised the Discussion headings and interpretation to use the same bounded terminology. For example, Section 4.1 is now titled “Beyond replacement: differentiated restructuring of POI-observed coffee–tea venue patterns.” In that section, the manuscript states that “the present study does not support understanding changes in POI-observed coffee–tea venue patterns as a simple linear substitution between coffee and tea.” This clarifies that the interpretation refers to observed venue patterns, not cultural transformation in a broad social or experiential sense.

Fourth, we revised the Conclusion to avoid overgeneralization. The revised Conclusion now states that “this study examined changes in POI-observed coffee–tea venue patterns in Jinan and Qingdao from 2018 to 2024.” It further concludes that “POI-observed coffee–tea venue restructuring is better understood as a differentiated process rather than as a single linear modernization pathway.” The final paragraph also clarifies that the findings should be interpreted “within the boundaries of POI-observed venue presence and spatial composition rather than as a complete representation of lived cultural practice or a causal account of underlying drivers.”

Together, these revisions directly address the concern by replacing broad “cultural transformation” claims with more precise language grounded in the POI-based evidence used in the study.

**Location:**

Abstract, lines 13–32;

Discussion, Section 4.1 “Beyond replacement: differentiated restructuring of POI-observed coffee–tea venue patterns”, lines 384–391;

Conclusion, especially the first and final paragraphs, lines 544–565.

## 【Q11】Define “teahouse” clearly; distinguish from milk-tea, beverage shops, and cafés.

**Comment:**

Define “teahouse” clearly; distinguish from milk-tea, beverage shops, and cafés.

**Response:**

Thank you for this important suggestion. We agree that the term “teahouse” should be clearly defined and distinguished from milk-tea shops, general beverage shops, cafés, and restaurants with incidental tea service. To address this issue, we revised the Methods section and S1 Appendix to provide an operational definition of teahouses and to clarify the exclusion rules for ambiguous or non-target beverage venues.

**Revision made:**

First, we clarified the definition of teahouses in the revised Methods. The manuscript now states that Dianping records were harmonized into two target venue classes, including “teahouses, defined as venues centered on on-site tea drinking, tea-room consumption, traditional teahouses, tea clubs, tea ceremony spaces, and comparable Chinese categories such as 茶馆, 茶楼, 茶室, and 茶社.” This definition makes clear that the study focuses on on-site tea-consumption spaces rather than all businesses selling tea-related products.

Second, we added a more detailed operational definition in S1 Appendix. Section S1.2.3 now states that “teahouses were defined as venues whose primary business identity was on-site tea drinking, tea-room consumption, or traditional tea-related social consumption.” The appendix further clarifies that this category includes “tea houses, tea rooms, traditional teahouses, tea clubs, tea ceremony spaces, and comparable Chinese categories such as 茶馆, 茶楼, 茶室, and 茶社 when the venue primarily functioned as an on-site tea consumption space.”

Third, we clarified the distinction between teahouses and non-target beverage venues. The revised Methods states that “milk-tea shops, fruit-tea shops, general beverage shops, dessert drink shops, restaurants with only incidental tea or coffee service, and ambiguous cafés without coffee-specific classification were excluded.” S1 Appendix further specifies that “milk-tea shops, fruit-tea shops, general beverage shops, dessert drink shops, restaurants with incidental tea service, non-coffee-oriented cafés, and ambiguous mixed beverage venues were excluded unless their primary business identity clearly matched the operational definition of coffee shop or teahouse.”

Fourth, we clarified how ambiguous cases were handled. S1 Appendix states that raw Dianping category labels were mapped to the target analytical classes through direct category mapping, cross-year reconciliation, keyword-assisted validation, and manual review of ambiguous cases. It further states that ambiguous cases were reviewed based on venue name, platform category label, and available descriptive information, and that records that could not be confidently assigned to either coffee shop or teahouse were excluded from the analytical coffee–tea dataset.

Together, these revisions define “teahouse” as an on-site tea-consumption venue and distinguish it from milk-tea shops, fruit-tea shops, general beverage shops, dessert drink shops, restaurants with incidental tea service, and ambiguous cafés or mixed beverage venues.

**Location:**

Methods, Section 2.2 “Study areas, POI data, and preprocessing”, lines 113–117;

S1 Appendix, Section S1.2.3 “Year-specific category harmonization for coffee-shop and teahouse venues.”

## 【Q12】Funding statement: correct singular “funder” to plural “funders”.

**Comment:**

Funding statement: correct singular “funder” → plural “funders”.

**Response:**

Thank you for pointing this out. We have corrected the grammatical issue in the Funding statement. Because two funding sources are listed, the plural form “funders” is appropriate.

**Revision made:**

The Funding statement has been revised to use the plural form: “The funders had no role in study design, data collection and analysis, decision to publish, or preparation of the manuscript.”

**Location:**

Funding statement, lines 581–585.

## 【Q13】Discussion repeats points; reduce redundancy and deepen interpretation of mechanisms.

**Comment:**

Discussion repeats points; reduce redundancy and deepen interpretation of mechanisms.

**Response:**

Thank you for this helpful suggestion. We agree that the Discussion in the previous version repeated some results and did not sufficiently develop the interpretation of why the two cities showed different coffee–tea venue patterns. To address this issue, we reorganized the Discussion, reduced repetitive restatement of empirical results, and strengthened the contextual interpretation of the findings. At the same time, we avoided claiming causal mechanisms that cannot be identified from the POI-based comparative design.

**Revision made:**

First, we restructured the Discussion into more focused subsections. The revised Discussion is organized around four interpretive themes: “Beyond replacement,” “Divergent city-specific pathways,” “Functional embedding as contextual association,” and “Implications for interpreting everyday consumption-venue restructuring.” This structure reduces repetition and allows each subsection to address a different interpretive issue rather than restating the Results section.

Second, we deepened the interpretation of the main city-level contrast. Section 4.1 now states that “the present study does not support understanding changes in POI-observed coffee–tea venue patterns as a simple linear substitution between coffee and tea.” It further interprets the observed process as “a form of restructuring characterized by local variation and spatial unevenness, rather than simple replacement.” This revision shifts the Discussion from repeating numerical results to interpreting the broader meaning of the comparison.

Third, we strengthened the city-specific interpretation in Section 4.2. The revised manuscript now states that “Jinan was characterized more by internal adjustment on the basis of overall expansion, whereas Qingdao was characterized more by directional compositional restructuring under conditions of relative stability in total scale.” For Jinan, the Discussion interprets the pattern as “bidirectional adjustment and rebalancing” within a more continuous everyday urban setting. For Qingdao, it interprets the pattern as “clearer coffee-oriented restructuring” under relatively stable total scale and within a coastal, tourism-oriented urban context. These revisions deepen the explanation of why the two cities followed different pathways without overstating causality.

Fourth, we revised the functional-zone discussion to emphasize contextual association rather than deterministic explanation. Section 4.3 now states that “different functional settings correspond to different activity flows, rhythms of stay, and consumption scenarios, which may help explain the observed differences in coffee–tea venue composition.” It also adds that the functional-zone correspondence “should not be interpreted as evidence of a single deterministic land-use mechanism.” This revision provides a more careful interpretation of the functional-zone results while addressing potential endogeneity.

Fifth, we revised Section 4.4 to synthesize the broader implications rather than repeat the empirical results. The revised text states that “changes in everyday consumption venues should not be understood as a single linear pathway in which the ‘modern’ replaces the ‘traditional’.” It further states that “coffee–tea venue restructuring in the two study cities is better understood as a process of ongoing adjustment, periodic fluctuation, and context-dependent reshaping.” This provides a clearer theoretical and practical takeaway from the comparative analysis.

Finally, we strengthened the Limitations section to clarify the interpretive boundary of the discussion. The revised manuscript states that the study remains “comparative rather than causal” and that trajectory types and functional zones “do not by themselves identify the independent effects of tourism, historical context, the COVID-19 period, or other background factors.” This ensures that the deeper interpretation remains appropriately bounded by the study design.

**Location:**

Discussion, Sections 4.1–4.5, lines 383–542.

## 【Q14】Include conceptual diagram linking CR, HI, CTI, trajectories, and functional zones for clarity.

**Comment:**

Include conceptual diagram linking CR, HI, CTI, trajectories, and functional zones for clarity.

**Response:**

Thank you for this helpful suggestion. We agree that a conceptual diagram can improve clarity by showing how the main indicators and analytical components are connected within the overall research design. To address this issue, we added a research-framework diagram to the revised manuscript and clarified the relationship among the fixed grid framework, the main cultural indicators, trajectory identification, and the functional-zone context.

**Revision made:**

First, we added a new Fig. 1 as a research-framework diagram. This figure summarizes the overall analytical workflow of the study, beginning with annual POI data for coffee shops and teahouses, the unified 500 m × 500 m grid framework, and the construction of the grid-year panel. It then shows how the main indicators were derived and used in the analysis.

**Fig. 1. Research framework for the grid-based analysis of coffee–tea cultural spaces**

Second, the revised Fig. 1 clarifies the role of each analytical component. It shows that Coffee Ratio (CR) and Hybridization Index (HI) were used to capture annual and long-run coffee–tea composition and coexistence balance; Cultural Transition Intensity (CTI) was used to measure endpoint-based directional change between 2018 and 2024; trajectory types were identified from annual CR sequences; and functional zones were used as contextual descriptors for interpreting the spatial embedding of coffee–tea venue patterns.

Third, we added an explicit reference to Fig. 1 in the Methods section and linked it to Table 1. The revised manuscript now states that “the overall analytical workflow is summarized in Fig. 1.” and that “key indicators and analytical concepts used in the comparative analysis are summarized in Table 1” Together, Fig. 1 and Table 1 clarify how CR, HI, CTI, trajectories, and functional zones are related within the comparative analytical framework.

These revisions directly address the reviewer’s suggestion by making the conceptual and analytical relationships among the main indicators more transparent.

**Location:**

Methods, Section 2.1 “Comparative study design and analytical framework”, lines 93–98; Fig. 1; Table 1.
